# Supplementary material for: Evolutionary dynamics of any multiplayer game on regular graphs
Source: Nat Commun. 2024 Jun 24;15:5349. doi: 10.1038/s41467-024-49505-5 (PMC11196707; doi:10.1038/s41467-024-49505-5)
Supplement: Supplementary file 1 — Supplementary Information [file 41467_2024_49505_MOESM1_ESM.pdf]

Supplementary Information for

Evolutionary dynamics of any multiplayer game on regular graphs

Chaoqian Wang, Matjaž Perc, & Attila Szolnoki

e-mail: CqWang814921147@outlook.com (C. Wang)

Contents

Supplementary Note 1: System construction and payoff calculation **3**

1.1 Expressing the system . . . 3

1.2 Payoff calculation of a j-player . . . 3

1.3 Payoff calculation of an i-player neighboring a j-player . . . 3

Supplementary Note 2: Pairwise comparison **4**

2.1 The increase of i-players . . . 4

2.2 The decrease of i-players . . . 5

2.3 The replicator equation . . . 5

2.4 Simplification and discussion . . . 6

2.4.1 Decomposition to accumulated payoff . . . 6

2.4.2 Decomposition to single-game payoff . . . 7

2.4.3 Special linear system . . . 9

2.5 Comparison with two-strategy dynamics . . . 10

Supplementary Note 3: Applications **11**

3.1 The traditional public goods game ($n = 2$) . . . 11

3.1.1 The well-mixed population . . . 12

3.1.2 The structured population . . . 12

3.1.3 When does pairwise comparison have no effect on evolution? . . . 12

3.2 Public goods games with peer punishment ($n = 3$) . . . 13

3.2.1 The well-mixed population . . . 13

3.2.2 The structured population . . . 15

3.2.3 Discussion . . . 17

3.3 Public goods games with pool punishment ($n = 3$) . . . 18

3.3.1 The well-mixed population . . . 19

3.3.2 The structured population . . . 20

3.3.3 Discussion . . . 24

3.4 Public goods games with the reward mechanism ($n = 3$) . . . 25

3.4.1 The well-mixed population . . . 25

3.4.2 The structured population . . . 27

3.4.3 Discussion . . . 30

3.5 The multi-stage public goods game ($n = 4$) . . . 31

3.5.1 The structured population . . . 33

3.5.2 Discussion . . . 36

Supplementary Note 4: Death-birth **36**

4.1 The increase of i-players . . . 36

4.2 The decrease of i-players . . . 37

4.3 The replicator equation . . . 38

4.4 Simplification and discussion . . . 38

4.4.1 Decomposition to accumulated payoff . . . 38

4.4.2 Decomposition to single-game payoff . . . 39
4.4.3 Special linear system . . . 40
4.5 Comparison with two-strategy dynamics . . . 41

Supplementary Note 5: Edge dynamics **42**
5.1 Pairwise comparison . . . 42
5.1.1 The increase of *il*-edges . . . 42
5.1.2 The decrease of *il*-edges . . . 43
5.1.3 Separation of different time scales . . . 43
5.2 Death-birth . . . 44
5.2.1 The increase of *il*-edges . . . 44
5.2.2 The decrease of *il*-edges . . . 45
5.2.3 Separation of different time scales . . . 45

Supplementary Note 6: Theorem on computation and operators **46**

Supplementary Note 1: System construction and payoff calculation

We employ the pair approximation method[1–5] to deduce the system dynamics. First, we express the system and explain the payoff calculation in detail.

1.1 Expressing the system

On the regular graph of degree k, the number of nodes is N and the number of edges is $kN/2$, where the population size $N \to \infty$. In this infinite population, the proportion of individuals who choose strategy i is denoted by x_i. The probability of finding a j-player neighboring an i player is denoted by $q_{j|i}$. The proportion of indirected ij-edges connecting a pair of strategy i and j is denoted by p_{ij}.

To sum up, there are $n+n^2+n^2$ variables to describe the system: n for x_i, n^2 for $q_{j|i}$, and n^2 for p_{ij}. They yield the following constraints.

$$\begin{aligned}
&\sum_{i=1}^{n} x_i = 1,\\
&\sum_{j=1}^{n} q_{j|i} = 1, \quad \text{for } i = 1,2,\dots,n,\\
&p_{ij} = x_i q_{j|i}, \quad \text{for } i = 1,2,\dots,n,\ j = 1,2,\dots,n,\\
&p_{ij} = p_{ji}, \quad \text{for } i = 1,2,\dots,n,\ j = 1,2,\dots,n,\ i \neq j.
\end{aligned} \tag{S1}$$

According to the third line in Eq. (S1), the system can be described by x_i and $q_{j|i}$, eliminating the need to use p_{ij}.

1.2 Payoff calculation of a j-player

As mentioned in the main text, to express a j-player's accumulated payoff, received from the multiplayer games organized by itself and neighbors, we need to introduce a variant of neighbor configuration $\mathbf{k}$ as follows.

$$\mathbf{k}_{+j} = (k_1, k_2, \dots, k_j + 1, \dots, k_n), \tag{S2}$$

where $\sum_{l=1}^{n} k_l = k-1$. This notation describes a configuration which has two components: (i) one individual chooses strategy j; (ii) among $k-1$ remaining individuals, $k_1, k_2, \dots, k_j, \dots, k_n$ individuals choose strategy $1,2,\dots,j,\dots,n$. Here, j could be an integer satisfying $1 \le j \le n$.

Consider a focal j-player with neighbor configuration $\mathbf{k}$. Then, the accumulated payoff of this j-player, denoted by $\pi_j^{\mathbf{k}}$, can be expressed as

$$\pi_j^{\mathbf{k}} = a_{j|\mathbf{k}} + \sum_{l=1}^{n} k_l \sum_{\sum_{\ell=1}^{n} k'_\ell = k-1} \frac{(k-1)!}{\prod_{\ell=1}^{n} k'_\ell !} \left(\prod_{\ell=1}^{n} q_{\ell|l}{}^{k'_\ell} \right) a_{j|\mathbf{k}'_{+l}}. \tag{S3}$$

It is straightforward that $a_{j|\mathbf{k}}$ is the payoff received from the game organized by the j-player itself. Furthermore, in the j-player's neighbor configuration $\mathbf{k}$, there are k_l individuals with strategy l.

In the game organized by an l-player, there must be a j-player, the focal player. We denote the neighbor configuration of the l-player as $\mathbf{k}'_{+j}$, where the upper right prime ($'$) is only to distinguish from $\mathbf{k}$. Here, $\mathbf{k}'_{+j}$ contains the focal j-player and the remaining $k-1$ uncertain ℓ-players, determined by going through $\sum_{\ell=1}^{n} k'_\ell = k-1$. Given that the l-player's co-player configuration in its own group is $\mathbf{k}'_{+j}$, the focal j-player's co-player configuration in the same group is then expressed as $\mathbf{k}'_{+l}$: removing itself and adding the l-player. As a result, $\mathbf{k}'_{+l}$ contains the l-player and the remaining $k-1$ uncertain ℓ-players. Therefore, the j-player receives payoff $a_{j|\mathbf{k}'_{+l}}$ from the games organized by each l-player neighbor by determining the remaining $k-1$ uncertain ℓ-co-players in $\mathbf{k}'_{+l}$.

1.3 Payoff calculation of an i-player neighboring a j-player

Now we consider a more complex case, the accumulated payoff of an i-player neighboring a focal j-player. To express the accumulated payoff of the i-player, we need to further introduce a variant of $\mathbf{k}$ as follows.

$$\mathbf{k}_{-i,+j} = (k_1, k_2, \dots, k_i - 1, \dots, k_j + 1, \dots, k_n), \tag{S4}$$

where $\sum_{l=1}^{n} k_l = k$. This notation describes a variant to the configuration $\mathbf{k}$, with one less individual choosing strategy i, and one more individual choosing strategy j. Here, i and j could be integers, satisfying $1 \le i \le n$ and $1 \le j \le n$. Moreover, the order of i and j ($i < j$ or $i > j$) does not matter in $\mathbf{k}_{-i,+j}$.

Again, we consider a focal j-player with neighbor configuration $\mathbf{k}$. The payoff of an i-player neighboring this focal j-player can be expressed as

$$\pi_{i|j}^{\mathbf{k}} = a_{i|\mathbf{k}_{-i,+j}} + \sum_{\sum_{l=1}^{n} k'_l = k-1} \frac{(k-1)!}{\prod_{l=1}^{n} k'_l!} \left(\prod_{l=1}^{n} q_{l|i}{}^{k'_l} \right) \left(a_{i|\mathbf{k}'_{+j}} + \sum_{l=1}^{n} k'_l \sum_{\sum_{\ell=1}^{n} k''_\ell = k-1} \frac{(k-1)!}{\prod_{\ell=1}^{n} k''_\ell!} \left(\prod_{\ell=1}^{n} q_{\ell|l}{}^{k''_\ell} \right) a_{i|\mathbf{k}''_{+l}} \right). \tag{S5}$$

In Eq. (S5), $a_{i|\mathbf{k}_{-i,+j}}$ is the payoff received from the game organized by the focal j-player. Note that $\mathbf{k}$ describes the neighbor configuration of the j-player. Therefore, i-player joining the game organized by the focal j-player, the remaining participants' configuration for this i-player becomes $\mathbf{k}_{-i,+j}$.

Let $\mathbf{k}'_{+j}$ denote the i-player's neighbor configuration. There is one neighbor employing strategy j, the focal j-player. The remaining $k-1$ neighbors are undetermined. Therefore, we go through $\sum_{l=1}^{n} k'_l = k-1$ for all possibilities of $\mathbf{k}'_{+j}$. In the game organized by the i-player itself, the payoff can be expressed as $a_{i|\mathbf{k}'_{+j}}$.

Among the i-player's remaining $k-1$ neighbors, the number of l-players is k'_l. We denote an l-player's neighbor configuration by $\mathbf{k}''_{+i}$: the i-player's strategy i is determined, and the remaining $k-1$ players are undetermined. We go through $\sum_{\ell=1}^{n} k''_\ell = k-1$ for all possibilities. While the configuration of other players around the l-player is $\mathbf{k}''_{+i}$, the configuration around the i-player should be rewritten by $\mathbf{k}''_{+l}$: removing the i-player and adding the l-player. Therefore, in the game organized by an l-player neighboring the i-player, the payoff of the i-player is $a_{i|\mathbf{k}''_{+l}}$.

Knowing the payoff calculation, we can study the evolutionary dynamics. Here, we investigate two update rules for the evolution of strategies, pairwise comparison and death-birth, presented in Supplementary Note 2 and Supplementary Note 4, respectively.

Supplementary Note 2: Pairwise comparison

In a unit time, a random focal individual is selected to update the strategy by comparing the fitness with a random neighbor. Suppose that the focal individual A compares its fitness with the neighbor B. The individual A adopts the strategy of B with the following probability:

$$W = \frac{F_B}{F_A + F_B}. \tag{S6}$$

Otherwise, individual A keeps its own strategy with the remaining probability $F_A/(F_A+F_B)$. Here, F_A and F_B are the fitness of individuals A and B. As mentioned in the main text, the transformation from payoff to fitness is $F = \exp(\delta\pi)$[6,7]. In this way, the adopting probability W has the following well-known form[8]:

$$W = \frac{1}{1+\exp\left[-\delta(\pi_B - \pi_A)\right]}, \tag{S7}$$

where π_A and π_B are the payoff of individuals A and B, and $\delta \to 0^+$ is the weak selection limit. According to Eq. (S7), if individual B has a higher payoff, then its strategy has a slightly higher probability to be adopted by individual A.

Below, we analyze the strategy evolution under pairwise comparison.

2.1 The increase of i-players

The increase of i-players happens when a focal j-player ($j \neq i$) is selected to update its strategy and an i-player takes the position. Given the focal j-player's neighbor configuration $\mathbf{k}$, the probability that an i-player takes the j-player's position is

$$\mathscr{P}(j \leftarrow i) = \frac{k_i}{k} \frac{F_{i|j}^{\mathbf{k}}}{F_j^{\mathbf{k}} + F_{i|j}^{\mathbf{k}}} = \frac{k_i}{2k} + \frac{k_i}{4k}(\pi_{i|j}^{\mathbf{k}} - \pi_j^{\mathbf{k}})\delta + \mathscr{O}(\delta^2). \tag{S8}$$

The randomly selected neighbor from $\mathbf{k}$ is an i-player with probability k_i/k. Then, the focal j-player adopts the i-player's strategy with the probability of pairwise comparison. Taylor expansion at $\delta \to 0^+$ has been performed in Eq. (S8).

Then, we apply Eq. (S8) to all possibilities for $j \neq i$ and the neighbor configuration $\mathbf{k}$, obtaining the probability that the number of i-players increases by 1 (i.e., the frequency of i-players increases by $1/N$) during a unit time step,

$$\mathscr{P}\left(\Delta x_i = \frac{1}{N}\right) = \sum_{j=1, j\neq i}^{n} x_j \sum_{\sum_{i'=1}^{n} k_{i'} = k} \frac{k!}{\prod_{i'=1}^{n} k_{i'}!} \left(\prod_{i'=1}^{n} q_{i'|j}{}^{k_{i'}} \right) \mathscr{P}(j \leftarrow i)$$

$$= \frac{1}{2}\sum_{j=1,j\neq i}^{n} x_j q_{i|j} + \frac{1}{4}\sum_{j=1,j\neq i}^{n} x_j \sum_{\sum_{i'=1}^{n} k_{i'}=k} \frac{k!}{\prod_{i'=1}^{n} k_{i'}!}\left(\prod_{i'=1}^{n} q_{i'|j}{}^{k_{i'}}\right)\frac{k_i}{k}\left(\pi_{i|j}^{\mathbf{k}} - \pi_j^{\mathbf{k}}\right)\delta + \mathscr{O}(\delta^2), \tag{S9}$$

where the notation i' is independent, only to distinguish from i.

2.2 The decrease of i-players

The decrease of i-players happens when a focal i-player is selected to update its strategy and the player who takes the position is not an i-player. Given the focal i-player's neighbor configuration $\mathbf{k}$, the probability that the player who takes the position is not an i-player is

$$\sum_{j=1,j\neq i}^{n} \mathscr{P}(i \leftarrow j) = \sum_{j=1,j\neq i}^{n} \frac{k_j}{k}\frac{F_{j|i}^{\mathbf{k}}}{F_i^{\mathbf{k}} + F_{j|i}^{\mathbf{k}}} = \frac{k-k_i}{2k} + \sum_{j=1,j\neq i}^{n} \frac{k_j}{4k}(\pi_{j|i}^{\mathbf{k}} - \pi_i^{\mathbf{k}})\delta + \mathscr{O}(\delta^2). \tag{S10}$$

Applying it to all possibilities for the neighbor configuration $\mathbf{k}$ after selecting a focal i-player with probability x_i, we obtain the probability that the number of i-players decreases by 1 during a unit time step,

$$\begin{aligned}\mathscr{P}\left(\Delta x_i = -\frac{1}{N}\right) &= x_i \sum_{\sum_{i'=1}^{n} k_{i'}=k} \frac{k!}{\prod_{i'=1}^{n} k_{i'}!}\left(\prod_{i'=1}^{n} q_{i'|i}{}^{k_{i'}}\right)\sum_{j=1,j\neq i}^{n}\mathscr{P}(i\leftarrow j)\\ &= \frac{x_i(1-q_{i|i})}{2} + \frac{1}{4}x_i \sum_{\sum_{i'=1}^{n} k_{i'}=k} \frac{k!}{\prod_{i'=1}^{n} k_{i'}!}\left(\prod_{i'=1}^{n} q_{i'|i}{}^{k_{i'}}\right)\sum_{j=1,j\neq i}^{n}\frac{k_j}{k}\left(\pi_{j|i}^{\mathbf{k}} - \pi_i^{\mathbf{k}}\right)\delta + \mathscr{O}(\delta^2).\end{aligned} \tag{S11}$$

2.3 The replicator equation

The instant change in the frequency x_i of i-players consists of the increase and decrease of i-players. Applying Eqs. (S9) and (S11), and considering that a full Monte Carlo step contains N elementary steps, we have

$$\begin{aligned}\dot{x}_i &= N \times \left\{\frac{1}{N}\mathscr{P}\left(\Delta x_i = \frac{1}{N}\right) + \left(-\frac{1}{N}\right)\mathscr{P}\left(\Delta x_i = -\frac{1}{N}\right)\right\}\\ &= \frac{1}{4}\sum_{j=1,j\neq i}^{n} x_j \sum_{\sum_{i'=1}^{n} k_{i'}=k} \frac{k!}{\prod_{i'=1}^{n} k_{i'}!}\left(\prod_{i'=1}^{n} q_{i'|j}{}^{k_{i'}}\right)\frac{k_i}{k}\left(\pi_{i|j}^{\mathbf{k}} - \pi_j^{\mathbf{k}}\right)\delta\\ &\quad - \frac{1}{4}x_i \sum_{\sum_{i'=1}^{n} k_{i'}=k} \frac{k!}{\prod_{i'=1}^{n} k_{i'}!}\left(\prod_{i'=1}^{n} q_{i'|i}{}^{k_{i'}}\right)\sum_{j=1,j\neq i}^{n}\frac{k_j}{k}\left(\pi_{j|i}^{\mathbf{k}} - \pi_i^{\mathbf{k}}\right)\delta + \mathscr{O}(\delta^2).\end{aligned} \tag{S12}$$

In Eq. (S12), the δ^0 term has been eliminated: applying Eq. (S1), we know $x_j q_{i|j} = p_{ij} = x_i q_{j|i}$ and $\sum_{j=1}^{n} q_{j|i} = 1$, such that the δ^0 term in Eq. (S9) can be expressed as $\frac{1}{2}\sum_{j=1,j\neq i}^{n} x_j q_{i|j} = \frac{1}{2}x_i\sum_{j=1,j\neq i}^{n} q_{j|i} = \frac{x_i(1-q_{i|i})}{2}$, which is equal to the one in Eq. (S9).

The δ^0 term being eliminated, the instant change in x_i happens on the order of δ^1 (which is the reason we must perform the Taylor expansion to δ^1 in Supplementary Notes 2.1 and 2.2). Meanwhile, the instant change in $q_{i|j}$ for $i,j = 1,2,\ldots,n$ happens on the order of δ^0 since the δ^0 term is non-zero (see Supplementary Note 5.1). That is, the change in $q_{i|j}$ is much faster than x_i, so that x_i changes on the basis of $q_{i|j}$ achieving equilibrium. According to Supplementary Note 5.1, we have the following solution when $q_{i|j}$ achieves stability.

$$q_{i|j} = \begin{cases}\dfrac{k-2}{k-1}x_i, & j\neq i,\\[2ex] \dfrac{k-2}{k-1}x_i + \dfrac{1}{k-1}, & j = i.\end{cases} \tag{S13}$$

The primitive replicator equation of x_i is Eq. (S12), where $\mathscr{O}(\delta^2) = 0$, $\pi_j^{\mathbf{k}}$ and $\pi_i^{\mathbf{k}}$ is given by Eq. (S3), $\pi_{i|j}^{\mathbf{k}}$ and $\pi_{j|i}^{\mathbf{k}}$ is given by Eq. (S5), and $q_{i'|j}$ is given by Eq. (S13). The degrees of freedom of the replicator dynamics system are $n-1$, represented by independent variables x_i ($i = 1,2,\ldots,n$, cancel one of them by $\sum_i^n x_i = 1$).

2.4 Simplification and discussion

The replicator equation given by Eq. (S12) can be further simplified and discussed. To do this, we need to introduce a useful equation, as given by Theorem 1 in Supplementary Note 6. For frequency quantities $0 \le z_j \le 1$ and an arbitrary function $g(\mathbf{k})$ of vector $\mathbf{k} = (k_1, k_2, \ldots, k_n)$, we have:

$$\sum_{\sum_{j=1}^n k_j = k} \frac{k!}{\prod_{j=1}^n k_j!} \left(\prod_{j=1}^n {z_j}^{k_j} \right) k_i g(\mathbf{k}) = k z_i \sum_{\sum_{j=1}^n k_j = k-1} \frac{(k-1)!}{\prod_{j=1}^n k_j!} \left(\prod_{j=1}^n {z_j}^{k_j} \right) g(\mathbf{k}_{+i}), \tag{S14}$$

which simplifies the expression by canceling the quantity k_i in the summation at the cost of changing $f(\mathbf{k})$ to $f(\mathbf{k}_{+i})$.

2.4.1 Decomposition to accumulated payoff

Using Eq. (S14), we can simplify the replicator equation (S12) to expected accumulated payoffs. That is, we can obtain an equation to avoid specific calculations of k_i in front of $\pi_{i|j}^{\mathbf{k}}$ and $\pi_j^{\mathbf{k}}$. The simplified replicator equation is

$$\begin{aligned}
\dot{x}_i = {} & \frac{\delta}{4} \sum_{j=1, j\neq i}^n x_j q_{i|j} \sum_{\sum_{i'=1}^n k_{i'} = k-1} \frac{(k-1)!}{\prod_{i'=1}^n k_{i'}!} \left(\prod_{i'=1}^n {q_{i'|j}}^{k_{i'}} \right) \left(\pi_{i|j}^{\mathbf{k}_{+i}} - \pi_j^{\mathbf{k}_{+i}} \right) \\
& - \frac{\delta}{4} x_i \sum_{\sum_{i'=1}^n k_{i'} = k-1} \frac{(k-1)!}{\prod_{i'=1}^n k_{i'}!} \left(\prod_{i'=1}^n {q_{i'|i}}^{k_{i'}} \right) \sum_{j=1, j\neq i}^n q_{j|i} \left(\pi_{j|i}^{\mathbf{k}_{+j}} - \pi_i^{\mathbf{k}_{+j}} \right) \\
= {} & \frac{\delta}{4} x_i \sum_{j=1, j\neq i}^n q_{j|i} \sum_{\sum_{i'=1}^n k_{i'} = k-1} \frac{(k-1)!}{\prod_{i'=1}^n k_{i'}!} \left(\prod_{i'=1}^n {q_{i'|j}}^{k_{i'}} \right) \left(\pi_{i|j}^{\mathbf{k}_{+i}} - \pi_j^{\mathbf{k}_{+i}} \right) \\
& - \frac{\delta}{4} x_i \sum_{j=1, j\neq i}^n q_{j|i} \sum_{\sum_{i'=1}^n k_{i'} = k-1} \frac{(k-1)!}{\prod_{i'=1}^n k_{i'}!} \left(\prod_{i'=1}^n {q_{i'|i}}^{k_{i'}} \right) \left(\pi_{j|i}^{\mathbf{k}_{+j}} - \pi_i^{\mathbf{k}_{+j}} \right).
\end{aligned} \tag{S15}$$

In this way, we only need to consider the calculation of $\pi_{i|j}^{\mathbf{k}_{+i}}$, $\pi_j^{\mathbf{k}_{+i}}$, $\pi_{j|i}^{\mathbf{k}_{+j}}$, $\pi_i^{\mathbf{k}_{+j}}$ summations.

As mentioned in the main text, let us denote that

$$\langle \pi_{i|X}^{\mathbf{k}_{+i}} \rangle = \sum_{\sum_{i'=1}^n k_{i'} = k-1} \frac{(k-1)!}{\prod_{i'=1}^n k_{i'}!} \left(\prod_{i'=1}^n {q_{i'|X}}^{k_{i'}} \right) \pi_{i|X}^{\mathbf{k}_{+i}}. \tag{S16}$$

The intuition of $\langle \pi_{i|X}^{\mathbf{k}_{+i}} \rangle$ is the expected accumulated payoff of an i-player neighboring an X-player. In this case, there must be an i-player in the X-player's neighbor configuration, which is the intuition of '$_{+i}$' in $\mathbf{k}_{+i}$.

We also denote that

$$\langle \pi_X^{\mathbf{k}_{+i}} \rangle = \sum_{\sum_{i'=1}^n k_{i'} = k-1} \frac{(k-1)!}{\prod_{i'=1}^n k_{i'}!} \left(\prod_{i'=1}^n {q_{i'|X}}^{k_{i'}} \right) \pi_X^{\mathbf{k}_{+i}}, \tag{S17}$$

which intuitively means the expected accumulated payoff of an X-player whose neighbor configuration contains at least one i-player.

Using the notations of Eqs. (S16) and (S17), the replicator equation of Eq. (S15) can be written as

$$\dot{x}_i = \frac{\delta}{4} x_i \sum_{j=1}^n q_{j|i} \left[\left(\langle \pi_{i|j}^{\mathbf{k}_{+i}} \rangle - \langle \pi_j^{\mathbf{k}_{+i}} \rangle \right) - \left(\langle \pi_{j|i}^{\mathbf{k}_{+j}} \rangle - \langle \pi_i^{\mathbf{k}_{+j}} \rangle \right) \right]. \tag{S18}$$

According to Theorem 2 in Supplementary Note 6, we have $\langle \pi_{i|j}^{\mathbf{k}_{+i}} \rangle = \langle \pi_i^{\mathbf{k}_{+j}} \rangle$, which can be equivalently written as $\langle \pi_{j|i}^{\mathbf{k}_{+j}} \rangle = \langle \pi_j^{\mathbf{k}_{+i}} \rangle$. Therefore, we have $\langle \pi_{j|i}^{\mathbf{k}_{+j}} \rangle - \langle \pi_i^{\mathbf{k}_{+j}} \rangle = \langle \pi_j^{\mathbf{k}_{+i}} \rangle - \langle \pi_{i|j}^{\mathbf{k}_{+i}} \rangle$, and Eq. (S18) can be simplified as

$$\dot{x}_i = \frac{\delta}{2} x_i \sum_{j=1}^n q_{j|i} \left(\langle \pi_{i|j}^{\mathbf{k}_{+i}} \rangle - \langle \pi_j^{\mathbf{k}_{+i}} \rangle \right). \tag{S19}$$

According to Theorem 3 in Supplementary Note 6, we have $\sum_{j=1}^{n} q_{j|i} \langle \pi_{i|j}^{\mathbf{k}_{+i}} \rangle = \langle \pi_i^{\mathbf{k}} \rangle$. In this way, we can further write Eq. (S19) as

$$\dot{x}_i = \frac{\delta}{2} x_i \left(\langle \pi_i^{\mathbf{k}} \rangle - \sum_{j=1}^{n} q_{j|i} \langle \pi_j^{\mathbf{k}_{+i}} \rangle \right), \tag{S20}$$

which bears an intuitive understanding if we introduce the following concepts:

- $\pi_i^{(0)} = \langle \pi_i^{\mathbf{k}} \rangle$, the expected accumulated payoff of the i-player itself (zero-step away on the graph).
- $\pi_i^{(1)} = \sum_{j=1}^{n} q_{j|i} \langle \pi_j^{\mathbf{k}_{+i}} \rangle$, the expected accumulated payoff of the i-player's first-order neighbors (one-step away on the graph).

Using these concepts, we know that $\dot{x}_i \propto x_i(\pi_i^{(0)} - \pi_i^{(1)})$ as mentioned in the main text. Under pairwise comparison, the reproduction rate of i-players depends on how much their expected accumulated payoff higher than neighbors, and the essence of replicator dynamics $\dot{x}_i$ is the competition between oneself and its first-order neighbors. This is consistent with the result obtained by the identity-by-descent idea[9,10], but we further generalize it to n-strategy systems in replicator dynamics.

To sum up, this section simplifies and divides the replicator equation into expected accumulated payoffs, focusing on elucidating the intuitive insights of the equation.

2.4.2 Decomposition to single-game payoff

Next, we further divide the replicator equation of Eq. (S20) into expected single-game payoffs, stressing the convenience for actual calculation.

For convenience, we introduce a notation to the payoff in a single game as also mentioned in the main text,

$$\langle a_{i|\mathbf{k}} \rangle_j = \sum_{\sum_{i'=1}^{n} k_{i'} = k} \frac{k!}{\prod_{i'=1}^{n} k_{i'}!} \left(\prod_{i'=1}^{n} q_{i'|j}{}^{k_{i'}} \right) a_{i|\mathbf{k}}, \tag{S21}$$

which means the expected payoff of an i-player in a single game with co-player configuration $\mathbf{k}$ found near a j-player. Please note that $\langle a_{i|\mathbf{k}} \rangle_j$ is only for introducing the concept and does not have actual physical sense, because an i-player cannot have all of its k co-players found near a j-player on a graph if $j \neq i$. Only expressions such as $\langle a_{i|\mathbf{k}} \rangle_i$, $\langle a_{i|\mathbf{k}_{+j}} \rangle_i$, and $\langle a_{i|\mathbf{k}_{+j}} \rangle_j$ are meaningful, which will be applied.

Applying the notation of Eq. (S21) to $\langle \pi_j^{\mathbf{k}_{+i}} \rangle$ in Eq. (S17) and also consider Eq. (S3), we have

$$\begin{aligned}
\langle \pi_j^{\mathbf{k}_{+i}} \rangle &= \sum_{\sum_{i'=1}^{n} k_{i'} = k-1} \frac{(k-1)!}{\prod_{i'=1}^{n} k_{i'}!} \left(\prod_{i'=1}^{n} q_{i'|j}{}^{k_{i'}} \right) \left[a_{j|\mathbf{k}_{+i}} + \sum_{\sum_{\ell=1}^{n} k'_\ell = k-1} \frac{(k-1)!}{\prod_{\ell=1}^{n} k'_\ell!} \left(\prod_{\ell=1}^{n} q_{\ell|i}{}^{k'_\ell} \right) a_{j|\mathbf{k}'_{+i}} \right. \\
&\quad \left. + \sum_{l=1}^{n} k_l \sum_{\sum_{\ell=1}^{n} k'_\ell = k-1} \frac{(k-1)!}{\prod_{\ell=1}^{n} k'_\ell!} \left(\prod_{\ell=1}^{n} q_{\ell|l}{}^{k'_\ell} \right) a_{j|\mathbf{k}'_{+l}} \right] \\
&= \sum_{\sum_{i'=1}^{n} k_{i'} = k-1} \frac{(k-1)!}{\prod_{i'=1}^{n} k_{i'}!} \left(\prod_{i'=1}^{n} q_{i'|j}{}^{k_{i'}} \right) a_{j|\mathbf{k}_{+i}} + \sum_{\sum_{\ell=1}^{n} k'_\ell = k-1} \frac{(k-1)!}{\prod_{\ell=1}^{n} k'_\ell!} \left(\prod_{\ell=1}^{n} q_{\ell|i}{}^{k'_\ell} \right) a_{j|\mathbf{k}'_{+i}} \\
&\quad + (k-1) \sum_{l=1}^{n} q_{l|j} \sum_{\sum_{\ell=1}^{n} k'_\ell = k-1} \frac{(k-1)!}{\prod_{\ell=1}^{n} k'_\ell!} \left(\prod_{\ell=1}^{n} q_{\ell|l}{}^{k'_\ell} \right) a_{j|\mathbf{k}'_{+l}} \\
&= \langle a_{j|\mathbf{k}_{+i}} \rangle_j + \langle a_{j|\mathbf{k}_{+i}} \rangle_i + (k-1) \sum_{l=1}^{n} q_{l|j} \langle a_{j|\mathbf{k}_{+l}} \rangle_l.
\end{aligned} \tag{S22}$$

The last step writes $\mathbf{k}'$ as $\mathbf{k}$ because the configurations are calculated within each $\langle \cdot \rangle$ separately. Intuitively, the expected accumulated payoff of an i-player's neighboring j-player $\langle \pi_j^{\mathbf{k}_{+i}} \rangle$ consists of the following components: $\langle a_{j|\mathbf{k}_{+i}} \rangle_j$ is from the game organized by the j-player, $\langle a_{j|\mathbf{k}_{+i}} \rangle_i$ is from the game organized by the i-player, and $\langle a_{j|\mathbf{k}_{+l}} \rangle_l$ is from the game organized by the remaining $k-1$ possible l-players neighboring the j-player.

Similarly, we have

$$\langle \pi_i^{\mathbf{k}} \rangle = \sum_{\sum_{i'=1}^{n} k_{i'} = k} \frac{k!}{\prod_{i'=1}^{n} k_{i'}!} \left(\prod_{i'=1}^{n} q_{i'|i}{}^{k_{i'}} \right) \pi_i^{\mathbf{k}}$$

$$
\begin{aligned}
&= \sum_{\sum_{i'=1}^{n} k_{i'}=k} \frac{k!}{\prod_{i'=1}^{n} k_{i'}!} \left(\prod_{i'=1}^{n} q_{i'|i}{}^{k_{i'}} \right) \left[a_{i|\mathbf{k}} + \sum_{l=1}^{n} k_l \sum_{\sum_{\ell=1}^{n} k'_\ell=k-1} \frac{(k-1)!}{\prod_{\ell=1}^{n} k'_\ell!} \left(\prod_{\ell=1}^{n} q_{\ell|l}{}^{k'_\ell} \right) a_{i|\mathbf{k}'_{+l}} \right] \\
&= \sum_{\sum_{i'=1}^{n} k_{i'}=k} \frac{k!}{\prod_{i'=1}^{n} k_{i'}!} \left(\prod_{i'=1}^{n} q_{i'|i}{}^{k_{i'}} \right) a_{i|\mathbf{k}} + k \sum_{l=1}^{n} q_{l|i} \sum_{\sum_{\ell=1}^{n} k'_\ell=k-1} \frac{(k-1)!}{\prod_{\ell=1}^{n} k'_\ell!} \left(\prod_{\ell=1}^{n} q_{\ell|l}{}^{k'_\ell} \right) a_{i|\mathbf{k}'_{+l}} \\
&= \langle a_{i|\mathbf{k}} \rangle_i + k \sum_{l=1}^{n} q_{l|i} \langle a_{i|\mathbf{k}'_{+l}} \rangle_l \\
&= \langle a_{i|\mathbf{k}} \rangle_i + k \sum_{j=1}^{n} q_{j|i} \langle a_{i|\mathbf{k}_{+j}} \rangle_j . \qquad \text{(S23)}
\end{aligned}
$$

The expected accumulated payoff of an i-player $\langle \pi_i^{\mathbf{k}} \rangle$ consists of the following components: $\langle a_{i|\mathbf{k}} \rangle_i$ is from the game organized by the i-player itself, and $\langle a_{i|\mathbf{k}_{+j}} \rangle_j$ is from the game organized by a neighboring j-player.

Using Eqs. (S22) and (S23), we can simplify the replicator equation of Eq. (S20) into expected single-game payoffs:

$$
\dot{x}_i = \frac{\delta}{2} x_i \left[\left(\langle a_{i|\mathbf{k}} \rangle_i + k \sum_{j=1}^{n} q_{j|i} \langle a_{i|\mathbf{k}_{+j}} \rangle_j \right) - \sum_{j=1}^{n} q_{j|i} \left(\langle a_{j|\mathbf{k}_{+i}} \rangle_j + \langle a_{j|\mathbf{k}_{+i}} \rangle_i + (k-1) \sum_{l=1}^{n} q_{l|j} \langle a_{j|\mathbf{k}_{+l}} \rangle_l \right) \right] . \qquad \text{(S24)}
$$

To decrease the number of elements, we use the relation $\langle a_{i|\mathbf{k}} \rangle_i = \sum_{j=1}^{n} q_{j|i} \langle a_{i|\mathbf{k}_{+j}} \rangle_i$ according to Theorem 4 in Supplementary Note 6. In this way, Eq. (S24) can be written as

$$
\dot{x}_i = \frac{\delta}{2} x_i \sum_{j=1}^{n} q_{j|i} \left(\langle a_{i|\mathbf{k}_{+j}} \rangle_i + k \langle a_{i|\mathbf{k}_{+j}} \rangle_j - \langle a_{j|\mathbf{k}_{+i}} \rangle_j - \langle a_{j|\mathbf{k}_{+i}} \rangle_i - (k-1) \sum_{j=1}^{n} q_{l|j} \langle a_{j|\mathbf{k}_{+l}} \rangle_l \right) . \qquad \text{(S25)}
$$

Using the relation between x_j and $q_{j|i}$ according to Eq. (S13), we can write Eq. (S25) again as

$$
\dot{x}_i = \frac{\delta(k-2)}{2(k-1)} x_i \sum_{j=1}^{n} x_j \left(\langle a_{i|\mathbf{k}_{+j}} \rangle_i + (k-1) \langle a_{i|\mathbf{k}_{+j}} \rangle_j + \langle a_{i|\mathbf{k}_{+i}} \rangle_i - \langle a_{j|\mathbf{k}_{+i}} \rangle_j - \langle a_{j|\mathbf{k}_{+i}} \rangle_i - (k-2) \sum_{j=1}^{n} x_l \langle a_{j|\mathbf{k}_{+l}} \rangle_l - \langle a_{j|\mathbf{k}_{+j}} \rangle_j \right) . \qquad \text{(S26)}
$$

For specific applications, we can employ Eq. (S25) or Eq. (S26) according to the actual needs. On the one hand, adopting Eq. (S25) cannot avoid subsequently transforming $q_{j|i}$ to x_j manually. We always need to transform $q_{j|i}$ to x_j to obtain the final replicator equation, but at which stage we compute it depends on the actual type of complexity. Adopting Eq. (S26) may be a quick solution at the cost of substituting additional $\langle a_{i|\mathbf{k}_{+i}} \rangle_i$ and $\langle a_{j|\mathbf{k}_{+j}} \rangle_j$ manually. However, we should note that the calculation inside each $\langle \cdot \rangle$ contains $q_{j|i}$ and we still need to transform them manually.

As mentioned in the main text, the advantage of Eqs. (S25) and (S26) is that we have attributed everything about $\langle \cdot \rangle$ into two types, the '$\langle a_{i|\mathbf{k}_{+j}} \rangle_i$ type' and the '$\langle a_{i|\mathbf{k}_{+j}} \rangle_j$ type'. They can be expressed by matrices by going through i and j:

- The $\langle a_{i|\mathbf{k}_{+j}} \rangle_i$ type:

$$
\left[\langle a_{i|\mathbf{k}_{+j}} \rangle_i \right]_{ij} = \begin{pmatrix} \langle a_{1|\mathbf{k}_{+1}} \rangle_1 & \langle a_{1|\mathbf{k}_{+2}} \rangle_1 & \cdots & \langle a_{1|\mathbf{k}_{+n}} \rangle_1 \\ \langle a_{2|\mathbf{k}_{+1}} \rangle_2 & \langle a_{2|\mathbf{k}_{+2}} \rangle_2 & \cdots & \langle a_{2|\mathbf{k}_{+n}} \rangle_2 \\ \vdots & \vdots & \ddots & \vdots \\ \langle a_{n|\mathbf{k}_{+1}} \rangle_n & \langle a_{n|\mathbf{k}_{+2}} \rangle_n & \cdots & \langle a_{n|\mathbf{k}_{+n}} \rangle_n \end{pmatrix} . \qquad \text{(S27)}
$$

- The $\langle a_{i|\mathbf{k}_{+j}} \rangle_j$ type:

$$
\left[\langle a_{i|\mathbf{k}_{+j}} \rangle_j \right]_{ij} = \begin{pmatrix} \langle a_{1|\mathbf{k}_{+1}} \rangle_1 & \langle a_{1|\mathbf{k}_{+2}} \rangle_2 & \cdots & \langle a_{1|\mathbf{k}_{+n}} \rangle_n \\ \langle a_{2|\mathbf{k}_{+1}} \rangle_1 & \langle a_{2|\mathbf{k}_{+2}} \rangle_2 & \cdots & \langle a_{2|\mathbf{k}_{+n}} \rangle_n \\ \vdots & \vdots & \ddots & \vdots \\ \langle a_{n|\mathbf{k}_{+1}} \rangle_1 & \langle a_{n|\mathbf{k}_{+2}} \rangle_2 & \cdots & \langle a_{n|\mathbf{k}_{+n}} \rangle_n \end{pmatrix} . \qquad \text{(S28)}
$$

Therefore, we attribute the problem to computing each elements in the aforementioned two types before substituting them to Eq. (S25) or Eq. (S26). The result of each element should be a function of $x_1, x_2, \ldots, x_n$ (transformed from $q_{j|i}$ manually) and game parameters.

There are n^2 elements in each matrix. Their diagonals are equal, meaning we can compute n fewer elements. Therefore, the total computation is $n^2 + n^2 - n = (2n-1)n$ elements. The computational complexity is $O(n^2)$, which is feasible within polynomial time.

2.4.3 Special linear system

We can further simplify the calculation when faced with special payoff structures. Although the payoff function $a_{i|\mathbf{k}}$ in a multiplayer game can be arbitrary, one of the most common cases is the linear payoff function. For example, the public goods game is a 2-strategy game with linear payoff function.

Given a co-player configuration $\mathbf{k}$, the linear payoff function is defined as containing only primary terms for $k_1, k_2, \ldots, k_n$ and a constant term. Let us denote the coefficient matrix $\mathbf{b}$ and the constant vector $\mathbf{c}$,

$$\mathbf{b} = \begin{pmatrix} b_{11} & b_{12} & \cdots & b_{1n} \\ b_{21} & b_{22} & \cdots & b_{2n} \\ \vdots & \vdots & \ddots & \vdots \\ b_{n1} & b_{n2} & \cdots & b_{nn} \end{pmatrix}, \ \mathbf{c} = \begin{pmatrix} c_1 \\ c_2 \\ \vdots \\ c_n \end{pmatrix}. \tag{S29}$$

Then, the payoff for different strategies in a single game with co-player configuration $\mathbf{k}$ can be expressed as

$$\begin{pmatrix} a_{1|\mathbf{k}} \\ a_{2|\mathbf{k}} \\ \vdots \\ a_{n|\mathbf{k}} \end{pmatrix} = \mathbf{b} \cdot \mathbf{k}^\top + \mathbf{c} = \begin{pmatrix} b_{11} & b_{12} & \cdots & b_{1n} \\ b_{21} & b_{22} & \cdots & b_{2n} \\ \vdots & \vdots & \ddots & \vdots \\ b_{n1} & b_{n2} & \cdots & b_{nn} \end{pmatrix} \begin{pmatrix} k_1 \\ k_2 \\ \vdots \\ k_n \end{pmatrix} + \begin{pmatrix} c_1 \\ c_2 \\ \vdots \\ c_n \end{pmatrix}, \tag{S30}$$

or

$$a_{i|\mathbf{k}} = \sum_{l=1}^{n} b_{il} k_l + c_i. \tag{S31}$$

Our goal is to obtain the simplified replicator equation for any linear payoff function depicted by $\mathbf{b}$ and $\mathbf{c}$. Making a small transformation to Eq. (S31), we have

$$a_{i|\mathbf{k}_{+j}} = \sum_{l=1}^{n} b_{il} k_l + b_{ij} + c_i. \tag{S32}$$

Importantly, we recall that a simple special case of Eq. (S14) (Theorem 1 in Supplementary Note 6) is

$$\sum_{\sum_{j=1}^{n} k_j = k} \frac{k!}{\prod_{j=1}^{n} k_j!} \left(\prod_{j=1}^{n} {z_j}^{k_j} \right) k_i = k z_i. \tag{S33}$$

In this way, we have

$$\langle a_{i|\mathbf{k}_{+j}} \rangle_i = (k-1) \sum_{l=1}^{n} b_{il} q_{l|i} + b_{ij} + c_i = (k-2) \sum_{l=1}^{n} b_{il} x_l + b_{ii} + b_{ij} + c_i, \tag{S34a}$$

$$\langle a_{i|\mathbf{k}_{+j}} \rangle_j = (k-1) \sum_{l=1}^{n} b_{il} q_{l|j} + b_{ij} + c_i = (k-2) \sum_{l=1}^{n} b_{il} x_l + 2b_{ij} + c_i, \tag{S34b}$$

which gives the elements of the $\langle a_{i|\mathbf{k}_{+j}} \rangle_i$ type and the $\langle a_{i|\mathbf{k}_{+j}} \rangle_j$ type. We can directly utilize the replicator equation divided into expected single-game payoffs given by Eq. (S26). After calculating and organizing, we obtain

$$\dot{x}_1 = \frac{\delta(k-2)}{2(k-1)} x_i \left((k+1)(\bar{\pi}_i - \bar{\pi}) + 3 \sum_{j=1}^{n} x_j (b_{ii} - b_{ij} - b_{ji} - b_{jj}) + 6 \sum_{j=1}^{n} \sum_{l=1}^{n} x_j x_l b_{jl} \right), \tag{S35}$$

where $\bar{\pi}_i$ is the mean payoff of i-players in a well-mixed population,

$$\bar{\pi}_i = k \sum_{l=1}^{n} x_l b_{il} + c_i, \tag{S36}$$

and $\bar{\pi}$ is the mean payoff of all individuals in a well-mixed population,

$$\bar{\pi} = \sum_{i=1}^{n} x_i \bar{\pi}_i = k \sum_{i=1}^{n} \sum_{l=1}^{n} x_i x_l b_{il} + \sum_{i=1}^{n} x_i c_i. \tag{S37}$$

We know that the replicator equation in a well-mixed population is $\dot{x}_i = x_i(\bar{\pi}_i - \bar{\pi})$. In this way, Eq. (S35) clearly shows the additional terms brought by pairwise comparison in a structured population compared to the well-mixed population.

2.5 Comparison with two-strategy dynamics

Let us discuss on how our replicator equations for n-strategy systems reduce to the 2-strategy system under pairwise comparison. When $n=2$, the configuration $\mathbf{k}=(k_1,k_2)=(k_1,k-k_1)$ can be represented by only k_1, where $k_1=0,1,\ldots,k$. Similarly, we have $\mathbf{k}_{+1}=(k_1+1,k-k_1-1)$, $\mathbf{k}_{+2}=(k_1,k-k_1+1)$, where $k_1=0,1,\ldots,k-1$.

For $n=2$, the accumulated payoff of a j-player with neighbor configuration $\mathbf{k}$ can be expressed as

$$\pi_j^{\mathbf{k}}=a_{j|\mathbf{k}}+k_1\sum_{k_1'=0}^{k-1}\frac{(k-1)!}{k_1'!(k-k_1'-1)!}{q_{1|1}}^{k_1'}{q_{2|1}}^{k-k_1'-1}a_{j|\mathbf{k}_{+1}'}+(k-k_1)\sum_{k_1'=0}^{k-1}\frac{(k-1)!}{k_1'!(k-k_1'-1)!}{q_{1|2}}^{k_1'}{q_{2|2}}^{k-k_1'-1}a_{j|\mathbf{k}_{+2}'},\tag{S38}$$

whereas the accumulated payoff of an i-player, neighboring a focal j-player with neighbor configuration $\mathbf{k}$, can be expressed as

$$\begin{aligned}\pi_{i|j}^{\mathbf{k}}=a_{i|\mathbf{k}_{-i,+j}}+\sum_{k_1'=0}^{k-1}\frac{(k-1)!}{k_1'!(k-k_1'-1)!}{q_{1|i}}^{k_1'}{q_{2|i}}^{k-k_1'-1}\Bigg(a_{i|\mathbf{k}_{+j}'}+k_1'\sum_{k_1''=0}^{k-1}\frac{(k-1)!}{k_1''!(k-k_1''-1)!}{q_{1|1}}^{k_1''}{q_{2|1}}^{k-k_1''-1}a_{i|\mathbf{k}_{+1}''}\\
+(k-k_1'-1)\sum_{k_1''=0}^{k-1}\frac{(k-1)!}{k_1''!(k-k_1''-1)!}{q_{1|2}}^{k_1''}{q_{2|2}}^{k-k_1''-1}a_{i|\mathbf{k}_{+2}''}\Bigg).\end{aligned}\tag{S39}$$

Comparing Eqs. (S12), (S15), (S18), and (S19), we first write the primitive n-strategy replicator equation of Eq. (S12) as follows:

$$\dot{x}_i=\frac{\delta}{2}\sum_{j=1,j\neq i}^{n}x_j\sum_{\sum_{i'=1}^{n}k_{i'}=k}\frac{k!}{\prod_{i'=1}^{n}k_{i'}!}\left(\prod_{i'=1}^{n}{q_{i'|j}}^{k_{i'}}\right)\frac{k_i}{k}\left(\pi_{i|j}^{\mathbf{k}}-\pi_j^{\mathbf{k}}\right).\tag{S40}$$

Then, we discuss the corresponding $n=2$ case. For $n=2$, the system state can be described by only one of x_1 and x_2, because $x_1+x_2=1$, $\dot{x}_1+\dot{x}_2=0$. Let us choose x_1 to describe the system. According to Eq. (S40), the replicator equation for $n=2$ can be written as

$$\dot{x}_1=\frac{\delta}{2}(1-x_1)\sum_{k_1=0}^{k}\frac{k!}{k_1!(k-k_1)!}{q_{1|2}}^{k_1}{q_{2|2}}^{k-k_1}\frac{k_1}{k}(\pi_{1|2}^{\mathbf{k}}-\pi_2^{\mathbf{k}}),\tag{S41}$$

which is complete for application. If we want to analyze Eq. (S41) further, we can do the following calculation.

$$\begin{aligned}\pi_{1|2}^{\mathbf{k}}-\pi_2^{\mathbf{k}}=&\,a_{1|\mathbf{k}_{-1,+2}}+\sum_{k_1'=0}^{k-1}\frac{(k-1)!}{k_1'!(k-k_1'-1)!}{q_{1|1}}^{k_1'}{q_{2|1}}^{k-k_1'-1}\Bigg(a_{1|\mathbf{k}_{+2}'}+k_1'\sum_{k_1''=0}^{k-1}\frac{(k-1)!}{k_1''!(k-k_1''-1)!}{q_{1|1}}^{k_1''}{q_{2|1}}^{k-k_1''-1}a_{1|\mathbf{k}_{+1}''}\\
&+(k-k_1'-1)\sum_{k_1''=0}^{k-1}\frac{(k-1)!}{k_1''!(k-k_1''-1)!}{q_{1|2}}^{k_1''}{q_{2|2}}^{k-k_1''-1}a_{1|\mathbf{k}_{+2}''}\Bigg)-a_{2|\mathbf{k}}\\
&-k_1\sum_{k_1'=0}^{k-1}\frac{(k-1)!}{k_1'!(k-k_1'-1)!}{q_{1|1}}^{k_1'}{q_{2|1}}^{k-k_1'-1}a_{2|\mathbf{k}_{+1}'}-(k-k_1)\sum_{k_1'=0}^{k-1}\frac{(k-1)!}{k_1'!(k-k_1'-1)!}{q_{1|2}}^{k_1'}{q_{2|2}}^{k-k_1'-1}a_{2|\mathbf{k}_{+2}'}\\
=&\,a_{1|\mathbf{k}_{-1,+2}}+\sum_{k_1'=0}^{k-1}\frac{(k-1)!}{k_1'!(k-k_1'-1)!}{q_{1|1}}^{k_1'}{q_{2|1}}^{k-k_1'-1}a_{1|\mathbf{k}_{+2}'}+(k-1)q_{1|1}\sum_{k_1'=0}^{k-1}\frac{(k-1)!}{k_1'!(k-k_1'-1)!}{q_{1|1}}^{k_1'}{q_{2|1}}^{k-k_1'-1}a_{1|\mathbf{k}_{+1}'}\\
&+(k-1)q_{2|1}\sum_{k_1'=0}^{k-1}\frac{(k-1)!}{k_1'!(k-k_1'-1)!}{q_{1|2}}^{k_1'}{q_{2|2}}^{k-k_1'-1}a_{1|\mathbf{k}_{+2}'}-a_{2|\mathbf{k}}\\
&-k_1\sum_{k_1'=0}^{k-1}\frac{(k-1)!}{k_1'!(k-k_1'-1)!}{q_{1|1}}^{k_1'}{q_{2|1}}^{k-k_1'-1}a_{2|\mathbf{k}_{+1}'}-(k-k_1)\sum_{k_1'=0}^{k-1}\frac{(k-1)!}{k_1'!(k-k_1'-1)!}{q_{1|2}}^{k_1'}{q_{2|2}}^{k-k_1'-1}a_{2|\mathbf{k}_{+2}'},\end{aligned}\tag{S42}$$

and

$$\sum_{k_1=0}^{k}\frac{k!}{k_1!(k-k_1)!}{q_{1|2}}^{k_1}{q_{2|2}}^{k-k_1}\frac{k_1}{k}(\pi_{1|2}^{\mathbf{k}}-\pi_2^{\mathbf{k}})$$

$$= q_{1|2}\sum_{k_1=0}^{k-1}\frac{(k-1)!}{k_1!(k-k_1-1)!}{q_{1|2}}^{k_1}{q_{2|2}}^{k-k_1-1}(a_{1|\mathbf{k}_{+2}}-a_{2|\mathbf{k}_{+1}})+q_{1|2}\sum_{k_1'=0}^{k-1}\frac{(k-1)!}{k_1'!(k-k_1'-1)!}{q_{1|1}}^{k_1'}{q_{2|1}}^{k-k_1'-1}a_{1|\mathbf{k}'_{+2}}$$

$$+q_{1|1}(k-1)q_{1|2}\sum_{k_1'=0}^{k-1}\frac{(k-1)!}{k_1'!(k-k_1'-1)!}{q_{1|1}}^{k_1'}{q_{2|1}}^{k-k_1'-1}a_{1|\mathbf{k}'_{+1}}+q_{2|1}(k-1)q_{1|2}\sum_{k_1'=0}^{k-1}\frac{(k-1)!}{k_1'!(k-k_1'-1)!}{q_{1|2}}^{k_1'}{q_{2|2}}^{k-k_1'-1}a_{1|\mathbf{k}'_{+2}}$$

$$-q_{1|2}[1+(k-1)q_{1|2}]\sum_{k_1'=0}^{k-1}\frac{(k-1)!}{k_1'!(k-k_1'-1)!}{q_{1|1}}^{k_1'}{q_{2|1}}^{k-k_1'-1}a_{2|\mathbf{k}'_{+1}}$$

$$-(k-1)q_{1|2}q_{2|2}\sum_{k_1'=0}^{k-1}\frac{(k-1)!}{k_1'!(k-k_1'-1)!}{q_{1|2}}^{k_1'}{q_{2|2}}^{k-k_1'-1}a_{2|\mathbf{k}'_{+2}}$$

$$= q_{1|2}\Big\{\langle a_{1|\mathbf{k}_{+2}}\rangle_1-\langle a_{2|\mathbf{k}_{+1}}\rangle_2+[(k-2)x_1+1]\left(\langle a_{1|\mathbf{k}_{+1}}\rangle_1-\langle a_{2|\mathbf{k}_{+1}}\rangle_1\right)$$
$$+[(k-2)(1-x_1)+1]\left(\langle a_{1|\mathbf{k}_{+2}}\rangle_2-\langle a_{2|\mathbf{k}_{+2}}\rangle_2\right)\Big\}. \tag{S43}$$

Applying Eq. (S43) to Eq. (S41), we can obtain Eq. (S44), which allows us to compute the replicator dynamics through expected single-game payoffs.

$$\dot{x}_1=\frac{\delta(k-2)}{2(k-1)}x_1(1-x_1)\Big\{\langle a_{1|\mathbf{k}_{+2}}\rangle_1-\langle a_{2|\mathbf{k}_{+1}}\rangle_2+[(k-2)x_1+1]\left(\langle a_{1|\mathbf{k}_{+1}}\rangle_1-\langle a_{2|\mathbf{k}_{+1}}\rangle_1\right)$$
$$+[(k-2)(1-x_1)+1]\left(\langle a_{1|\mathbf{k}_{+2}}\rangle_2-\langle a_{2|\mathbf{k}_{+2}}\rangle_2\right)\Big\}. \tag{S44}$$

To be sure, Eq. (S44) can also be obtained by applying $n=2$ to Eq. (S26) directly.

Next, we present the case of $n=2$ for special linear systems. According to Eq. (S35), when it is sufficient for **b** and **c** to describe the payoff structure, the replicator dynamics for $n=2$ can be simplified as

$$\dot{x}_1=\frac{\delta(k-2)(k+1)}{2(k-1)}\left[x_1(\bar{\pi}_1-\bar{\pi})+\frac{3}{k+1}x_1(1-x_1)(1-2x_1)(b_{11}-b_{12}-b_{21}+b_{22})\right], \tag{S45}$$

where $x_1(\bar{\pi}_1-\bar{\pi})$ corresponds to the replicator dynamics in a well-mixed population, which can be obtained by the knowledge for well-mixed populations. Using **b** and **c** to express the payoff, we can also write them as

$$x_1(\bar{\pi}_1-\bar{\pi})=x_1(1-x_1)[k(b_{11}-b_{21})x_1+k(b_{12}-b_{22})(1-x_1)+c_1-c_2]. \tag{S46}$$

Supplementary Note 3: Applications

We give five examples here to demonstrate how to apply our general replicator equations to specific models. We include the traditional public goods game ($n=2$), public goods games with peer punishment ($n=3$, linear) & pool punishment ($n=3$, nonlinear), public goods games with the reward mechanism ($n=3$, linear), and the multi-stage public goods game ($n=4$, linear). It is worth noting that all applications are done under the pairwise comparison rule for strategy evolution.

3.1 The traditional public goods game ($n=2$)

The traditional public goods game in structured populations has been studied under both pairwise comparison[7,11] and death-birth[3,12]. Here, we present the results under pairwise comparison within our framework.

As mentioned in the main text, there are $n=2$ strategies in the traditional public goods game:

$1=$ Cooperation (C);

$2=$ Defection (D).

Cooperation means investing c in the common pool containing the co-players in **k** and oneself. Defection means investing nothing. The investment of all these $k+1$ players (k_1c for a focal defector and $(k_1+1)c$ for a focal cooperator) is enlarged by a synergy factor r ($r>1$). The resultant public goods (rk_1c for a focal defector and $r(k_1+1)c$ for a focal cooperator) are equally distributed to all $k+1$ players. Therefore, for co-player configuration $\mathbf{k}=(k_1,k_2)$, we have the following payoff calculation in a single game,

$$a_{1|\mathbf{k}}=\frac{r(k_1+1)c}{k+1}-c=\frac{rc}{k+1}k_1+\frac{rc}{k+1}-c, \tag{S47a}$$

$$a_{2|\mathbf{k}}=\frac{rk_1c}{k+1}=\frac{rc}{k+1}k_1. \tag{S47b}$$

3.1.1 The well-mixed population

The frequencies of strategies 1 and 2 are denoted by x_1 and $x_2 = 1 - x_1$. In a well-mixed population, the mean payoffs of the two strategies are calculated as follows.

$$\bar{\pi}_1 = \sum_{k_1=0}^{k} \frac{k!}{k_1!(k-k_1)!} x_1^{k_1}(1-x_1)^{k-k_1} a_{1|\mathbf{k}} = \frac{rc}{k+1} k x_1 + \frac{rc}{k+1} - c, \tag{S48a}$$

$$\bar{\pi}_2 = \sum_{k_1=0}^{k} \frac{k!}{k_1!(k-k_1)!} x_1^{k_1}(1-x_1)^{k-k_1} a_{2|\mathbf{k}} = \frac{rc}{k+1} k x_1. \tag{S48b}$$

The mean payoff of the total population is

$$\bar{\pi} = x_1\bar{\pi}_1 + (1-x_1)\bar{\pi}_2 = (r-1)c x_1. \tag{S49}$$

According to the traditional replicator dynamics in well-mixed populations, we have

$$\dot{x}_1 = x_1(\bar{\pi}_1 - \bar{\pi}) = x_1(1-x_1)\left(\frac{rc}{k+1} - c\right). \tag{S50}$$

It is clear that the system has two equilibrium points, $x_1 = 0$ and $x_1 = 1$. When $r < k+1$, the system is stable at $x_1 = 0$. When $r > k+1$, the system is stable at $x_1 = 1$.

3.1.2 The structured population

We observe that the payoff structure in Eq. (S47) is a linear function of $\mathbf{k}$. That is, the payoff in a single game can be expressed as $(a_{1|\mathbf{k}}, a_{2|\mathbf{k}})^\top = \mathbf{b}\cdot\mathbf{k}^\top + \mathbf{c}$, where

$$\mathbf{b} = \begin{pmatrix} \frac{rc}{k+1} & 0 \\ \frac{rc}{k+1} & 0 \end{pmatrix}, \quad \mathbf{c} = \begin{pmatrix} \frac{rc}{k+1} - c \\ 0 \end{pmatrix}. \tag{S51}$$

Therefore, we can utilize the conclusion of special linear systems given by Eq. (S45). The replicator equation for the traditional public goods game in a structured population is

$$\dot{x}_1 = \frac{\delta(k-2)(k+1)}{2(k-1)} x_1(\bar{\pi}_1 - \bar{\pi}) = \frac{\delta(k-2)(k+1)}{2(k-1)} x_1(1-x_1)\left(\frac{rc}{k+1} - c\right). \tag{S52}$$

The coefficient in front of $x_1(\bar{\pi}_1 - \bar{\pi})$ does not affect the equilibrium points and stability. Therefore, we can say that evolutionary dynamics of the public goods game in structured populations under pairwise comparison has no difference from the well-mixed population in the weak selection limit. We can attribute this to $b_{11} - b_{12} - b_{21} + b_{22} = 0$.

3.1.3 When does pairwise comparison have no effect on evolution?

It is an existing conclusion in previous literature that the equilibrium points and stability of public goods games under pairwise comparison in structured populations are equivalent to the well-mixed population[7]. This is also the reason why many works prefer death-birth when studying structured populations[3,7,12–14], whose evolutionary dynamics can make difference from the well-mixed population.

To our knowledge, there is no previous findings of an equivalence between the PC rule in well-mixed and structured populations for nonlinear payoff functions. The accidental equivalence happens for linear payoff functions, such as the public goods game. Nonetheless, the linearity does not necessarily lead to the equivalence between structured and well-mixed populations. For example, all two-player games are linear. While the prisoner's dilemma game is equivalent in structured and well-mixed populations under the PC rule, the snowdrift is not[2].

Here, we conclude the general condition for linear multiplayer games, that pairwise comparison in a structured population has no effect on evolution compared to well-mixed populations. This is to let the terms other than $x_i(\bar{\pi}_i - \bar{\pi})$ equal zero in Eq. (S35), that is,

$$b_{ii} - \sum_{j=1}^{n} x_j(b_{ij} + b_{ji} + b_{jj}) + 2\sum_{j=1}^{n}\sum_{l=1}^{n} x_j x_l b_{jl} = 0, \tag{S53}$$

for $i = 1, 2, \ldots, n$. Once Eq. (S53) is satisfied, we have $\dot{x}_i \propto x_i(\bar{\pi}_i - \bar{\pi})$ and pairwise comparison in structured populations makes no difference compared to the well-mixed population. We can see that the condition is only dependent of $\mathbf{b}$ but independent of

c. For 2-strategy linear multiplayer games, the condition reduces to $b_{11} - b_{12} - b_{21} + b_{22} = 0$, which is accidentally the case of the public goods game.

Using the judgement of Eq. (S53), we know that the public goods game is not the only multiplayer game where the PC rule plays no role. The multi-stage public goods game, which is a 4-strategy system[15], is also an example equivalent in structured and well-mixed populations under weak selection and the PC rule, which we will show in Supplementary Note 3.5.

One may say that using other update rules, such as death-birth, can avoid the accidental equivalence between structured and well-mixed populations under weak selection and can make new findings. However, we would like to stress an advantage of the equivalence under pairwise comparison. We have known that the traditional public goods game with the PC rule generates the same results as in the well-mixed population. That is to say, we can avoid blurring the effect of structured populations on the traditional 2-strategy model when we introduce a third strategy. When introducing the third strategy to the traditional public goods game and studying the coevolutionary games of more than two strategies, we can compare the structured and well-mixed populations to obtain the difference created by the additional strategies only. This cannot be realized by the death-birth rule. In other words, the PC rule focuses on the independent role of additional strategies in structured populations, while the death-birth rule would mix the effect of the traditional two strategies in structured populations, reducing the scientific validity of the conclusions on the additional strategies.

3.2 Public goods games with peer punishment ($n = 3$)

As mentioned in the main text, there are $n = 3$ strategies in the public goods game with peer punishment[16]:

1 = Cooperation (C);

2 = Defection (D);

3 = Peer punishment (E).

Based on the traditional public goods game, the peer punishment strategy is introduced as an additional strategy. A punishing player pays a cost α to punish a defective co-player. The punished defective player is charged with a fine β. As a result, given k_2 defective co-players, a punishing player has αk_2 paid. Similarly, given k_3 punishing co-players, a defective player has βk_3 charged. Meanwhile, we assume punishing players also perform the cooperative behavior, investing c to the common pool. This makes the cooperative players second-order free-riders.

Given the co-player configuration $\mathbf{k} = (k_1, k_2, k_3)$, we have the following payoff calculation in a single game.

$$a_{1|\mathbf{k}} = \frac{r(k_1+1+k_3)c}{k+1} - c = \frac{rc}{k+1}k_1 + \frac{rc}{k+1}k_3 + \frac{rc}{k+1} - c, \tag{S54a}$$

$$a_{2|\mathbf{k}} = \frac{r(k_1+k_3)c}{k+1} - \beta k_3 = \frac{rc}{k+1}k_1 + \left(\frac{rc}{k+1} - \beta\right)k_3, \tag{S54b}$$

$$a_{3|\mathbf{k}} = \frac{r(k_1+k_3+1)c}{k+1} - c - \alpha k_2 = \frac{rc}{k+1}k_1 - \alpha k_2 + \frac{rc}{k+1}k_3 + \frac{rc}{k+1} - c. \tag{S54c}$$

3.2.1 The well-mixed population

The frequencies of strategies 1, 2, 3 are denoted by x_1, x_2, and x_3 (or x_C, x_D, and x_E in the main text for straightforward understanding), respectively. In a well-mixed population, the mean payoffs of the three strategies are calculated as follows.

$$\bar{\pi}_1 = \sum_{k_1+k_2+k_3=k} \frac{k!}{k_1!k_2!k_3!} {x_1}^{k_1} {x_2}^{k_2} {x_3}^{k_3} a_{1|\mathbf{k}} = \frac{rc}{k+1}kx_1 + \frac{rc}{k+1}kx_3 + \frac{rc}{k+1} - c, \tag{S55a}$$

$$\bar{\pi}_2 = \sum_{k_1+k_2+k_3=k} \frac{k!}{k_1!k_2!k_3!} {x_1}^{k_1} {x_2}^{k_2} {x_3}^{k_3} a_{2|\mathbf{k}} = \frac{rc}{k+1}kx_1 + \left(\frac{rc}{k+1} - \beta\right)kx_3, \tag{S55b}$$

$$\bar{\pi}_3 = \sum_{k_1+k_2+k_3=k} \frac{k!}{k_1!k_2!k_3!} {x_1}^{k_1} {x_2}^{k_2} {x_3}^{k_3} a_{3|\mathbf{k}} = \frac{rc}{k+1}kx_1 - \alpha kx_2 + \frac{rc}{k+1}kx_3 + \frac{rc}{k+1} - c. \tag{S55c}$$

The mean payoff of the total population is then calculated by

$$\bar{\pi} = x_1\bar{\pi}_1 + x_2\bar{\pi}_2 + x_3\bar{\pi}_3 = \frac{rc}{k+1}kx_1 + \frac{rc}{k+1}kx_3 + (x_1+x_3)\left(\frac{rc}{k+1} - c\right) - kx_2x_3(\alpha+\beta). \tag{S56}$$

On this basis, we can write the replicator equations of the well-mixed population $\dot{x}_i = x_i(\bar{\pi}_i - \bar{\pi})$ as follows.

$$\dot{x}_1 = x_1(\bar{\pi}_1 - \bar{\pi}) = x_1\left[(1-x_1-x_3)\left(\frac{rc}{k+1} - c\right) + kx_2x_3(\alpha+\beta)\right], \tag{S57a}$$

$$\dot{x}_2 = x_2(\bar{\pi}_2 - \bar{\pi}) = x_2\left[-(x_1+x_3)\left(\frac{rc}{k+1}-c\right)+k\left(x_2x_3(\alpha+\beta)-x_3\beta\right)\right], \tag{S57b}$$

$$\dot{x}_3 = x_3(\bar{\pi}_3 - \bar{\pi}) = x_3\left[(1-x_1-x_3)\left(\frac{rc}{k+1}-c\right)+k\left(x_2x_3(\alpha+\beta)-x_2\alpha\right)\right]. \tag{S57c}$$

We denote the system state $\mathbf{x} = (x_1, x_2, x_3)$. Solving $\dot{\mathbf{x}} = \mathbf{0}$, we obtain equilibrium points, which can be divided into three categories. The first and second categories are single equilibrium points: a point on the D-vertex, $\mathbf{x}^{(D)} = (0,1,0)$, and a point on the DE-edge, $\mathbf{x}^{(DE)} = (0, x_2^{(DE)}, x_3^{(DE)})$, where

$$x_2^{(DE)} = \frac{1}{k(\alpha+\beta)}\left(\frac{rc}{k+1}-c+k\beta\right), \tag{S58a}$$

$$x_3^{(DE)} = 1 - x_1^{(DE)} = \frac{1}{k(\alpha+\beta)}\left(-\frac{rc}{k+1}+c+k\alpha\right). \tag{S58b}$$

The third category contains infinite equilibrium points on the CE-edge, denoted by $\mathbf{x}^{(CE)} = (x_1^{(CE)}, 0, x_3^{(CE)})$, where $0 \le x_1^{(CE)} \le 1$, $0 \le x_3^{(CE)} \le 1$, $x_1^{(CE)} + x_3^{(CE)} = 1$. This category covers other two vertex points $(1,0,0)$ and $(0,0,1)$.

The stability of $\mathbf{x}^{(D)}$ and $\mathbf{x}^{(DE)}$ can be studied by the regular method. We cancel $x_2 = 1 - x_1 - x_3$ and study the dynamics depicted by $\dot{x}_1$ and $\dot{x}_3$,

$$\dot{x}_1 = x_1(1-x_1-x_3)\left(\frac{rc}{k+1}-c+kx_3(\alpha+\beta)\right), \tag{S59a}$$

$$\dot{x}_3 = x_3(1-x_1-x_3)\left(\frac{rc}{k+1}-c+kx_3(\alpha+\beta)-k\alpha\right). \tag{S59b}$$

The Jacobian matrix of system (S59) is

$$J = \begin{pmatrix} \frac{\partial \dot{x}_1}{\partial x_1} & \frac{\partial \dot{x}_1}{\partial x_3} \\ \frac{\partial \dot{x}_3}{\partial x_1} & \frac{\partial \dot{x}_3}{\partial x_3} \end{pmatrix}$$
$$= \begin{pmatrix} (1-2x_1-x_3)\left(\frac{rc}{k+1}-c+kx_3(\alpha+\beta)\right) & -x_1\left(\frac{rc}{k+1}-c+k(1-x_1)(\alpha+\beta)\right) \\ -x_3\left(\frac{rc}{k+1}-c+kx_3(\alpha+\beta)-k\alpha\right) & (1-x_1-2x_3)\left(\frac{rc}{k+1}-c-k\alpha\right)+(2-2x_1-3x_3)kx_3(\alpha+\beta) \end{pmatrix}. \tag{S60}$$

Substituting the value of $\mathbf{x}^{(D)}$ into Eq. (S60), we have

$$J|_{\mathbf{x}=\mathbf{x}^{(D)}} = \begin{pmatrix} \frac{rc}{k+1}-c & 0 \\ 0 & \frac{rc}{k+1}-c-k\alpha \end{pmatrix}. \tag{S61}$$

The conditions ensuring $J|_{\mathbf{x}=\mathbf{x}^{(D)}}$ negative-definite are $r < k+1$ and $r < (1+k\alpha/c)(k+1)$. Considering $k+1 < (1+k\alpha/c)(k+1)$, we know that $\mathbf{x}^{(D)}$ is stable if and only if $r < k+1$.

Substituting the value of $\mathbf{x}^{(DE)}$ into Eq. (S60), we have

$$J|_{\mathbf{x}=\mathbf{x}^{(DE)}} = \begin{pmatrix} (1-x_3^{(DE)})\left(\frac{rc}{k+1}-c+kx_3^{(DE)}(\alpha+\beta)\right) & 0 \\ -x_3^{(DE)}\left(\frac{rc}{k+1}-c+kx_3^{(DE)}(\alpha+\beta)-k\alpha\right) & (1-2x_3^{(DE)})\left(\frac{rc}{k+1}-c-k\alpha\right)+(2-3x_3^{(DE)})kx_3^{(DE)}(\alpha+\beta) \end{pmatrix}. \tag{S62}$$

The first-order sequential principal subformula is

$$(1-x_3^{(DE)})\left(\frac{rc}{k+1}-c+kx_3^{(DE)}(\alpha+\beta)\right) = (1-x_3^{(DE)})\left(\frac{rc}{k+1}-c+\left(-\frac{rc}{k+1}+c+k\alpha\right)\right) = (1-x_3^{(DE)})k\alpha > 0. \tag{S63}$$

Therefore, $\mathbf{x}^{(DE)}$ is unstable.

Next, we study the stability of $\mathbf{x}^{(CE)} = (x_1^{(CE)}, 0, x_3^{(CE)})$, which contains infinite equilibrium points satisfying $x_1^{(CE)} + x_3^{(CE)} = 1$. This is because when $x_2 = 0$, we have $\dot{x}_1 = \dot{x}_3$ and strategies 1 and 3 are indistinguishable. Concerning this, we can treat $x_1 + x_3$ as a whole and study the system depicted by $\dot{x}_2$ and $\dot{x}_1 + \dot{x}_3$. We cancel $x_1 + x_3 = 1 - x_2$ and have

$$\dot{x}_2 = -x_2(1-x_2)\left(\frac{rc}{k+1} - c\right) + kx_2x_3\left(x_2(\alpha+\beta) - \beta\right). \tag{S64}$$

The element in the single-order Jacobian matrix is

$$\frac{\mathrm{d}\dot{x}_2}{\mathrm{d}x_2} = -(1-2x_2)\left(\frac{rc}{k+1} - c\right) + kx_3\left(2x_2(\alpha+\beta) - \beta\right). \tag{S65}$$

Substituting the value of $\mathbf{x}^{(CE)}$ into $\mathrm{d}\dot{x}_2/\mathrm{d}x_2$, we have

$$\left.\frac{\mathrm{d}\dot{x}_2}{\mathrm{d}x_2}\right|_{\mathbf{x}=\mathbf{x}^{(CE)}} = -\left(\frac{rc}{k+1} - c\right) - kx_3^{(CE)}\beta. \tag{S66}$$

Therefore, $\mathbf{x}^{(CE)}$ is stable if $x_1^{(CE)} < x_{1,\star}^{(CE)}$ (or $x_3^{(CE)} > x_{3,\star}^{(CE)}$), where

$$x_{1,\star}^{(CE)} = 1 + \left(\frac{r}{k+1} - 1\right)\frac{c}{k\beta} \equiv \left(1 + \frac{\alpha}{\beta}\right)x_2^{(DE)}, \tag{S67}$$

or $x_{3,\star}^{(CE)} = \left(-\frac{r}{k+1} + 1\right)\frac{c}{k\beta}$. That is, while every point on the CE-edge is equilibrium, only the points on the side of $x_1^{(CE)} < x_{1,\star}^{(CE)}$ are stable. Moreover, we note that $x_{1,\star}^{(CE)} > 1$ when $r > k+1$. That is, $x_1^{(CE)} < x_{1,\star}^{(CE)}$ always holds and the points on the CE-edge are stable everywhere for $r > k+1$.

3.2.2 The structured population

We notice that the payoff structure given by Eq. (S54) is linear, which means that we can utilize the simplified method for special linear systems given by Supplementary Note 2.4.3 for convenience.

Comparing the payoff structure $a_{i|\mathbf{k}}$ in Eq. (S54) with Eqs. (S29)–(S31), we extract matrices $\mathbf{b}$ and $\mathbf{c}$,

$$\mathbf{b} = \begin{pmatrix} \frac{rc}{k+1} & 0 & \frac{rc}{k+1} \\ \frac{rc}{k+1} & 0 & \frac{rc}{k+1} - \beta \\ \frac{rc}{k+1} & -\alpha & \frac{rc}{k+1} \end{pmatrix}, \quad \mathbf{c} = \begin{pmatrix} \frac{rc}{k+1} - c \\ 0 \\ \frac{rc}{k+1} - c \end{pmatrix}. \tag{S68}$$

According to Eq. (S35), let us calculate

$$3\sum_{j=1}^{3} x_j(b_{11} - b_{1j} - b_{j1} - b_{jj}) = -6(x_1+x_3)\frac{rc}{k+1}, \tag{S69a}$$

$$3\sum_{j=1}^{3} x_j(b_{22} - b_{2j} - b_{j2} - b_{jj}) = -6(x_1+x_3)\frac{rc}{k+1} + 3x_3(\alpha+\beta), \tag{S69b}$$

$$3\sum_{j=1}^{3} x_j(b_{33} - b_{3j} - b_{j3} - b_{jj}) = -6(x_1+x_3)\frac{rc}{k+1} + 3x_2(\alpha+\beta), \tag{S69c}$$

$$6\sum_{j=1}^{3}\sum_{l=1}^{3} x_jx_lb_{jl} = 6(x_1+x_3)\frac{rc}{k+1} - 6x_2x_3(\alpha+\beta). \tag{S69d}$$

Then, we can substitute them into Eq. (S35). Meanwhile, the expression of $\bar{\pi}_i$ and $\bar{\pi}$ is the same as we give in Supplementary Note 3.2.1 for well-mixed populations. In this way, we obtain the replicator equations in structured populations as follows.

$$\dot{x}_1 = \frac{\delta(k-2)}{2(k-1)}x_1\left\{(k+1)\left[(1-x_1-x_3)\left(\frac{rc}{k+1} - c\right) + kx_2x_3(\alpha+\beta)\right] - 6x_2x_3(\alpha+\beta)\right\}, \tag{S70a}$$

$$\dot{x}_2 = \frac{\delta(k-2)}{2(k-1)} x_2 \left\{ (k+1)\left[-(x_1+x_3)\left(\frac{rc}{k+1}-c\right) + k\left(x_2x_3(\alpha+\beta)-x_3\beta\right)\right] - 6x_2x_3(\alpha+\beta) + 3x_3(\alpha+\beta) \right\}, \tag{S70b}$$

$$\dot{x}_3 = \frac{\delta(k-2)}{2(k-1)} x_3 \left\{ (k+1)\left[(1-x_1-x_3)\left(\frac{rc}{k+1}-c\right) + k\left(x_2x_3(\alpha+\beta)-x_2\alpha\right)\right] - 6x_2x_3(\alpha+\beta) + 3x_2(\alpha+\beta) \right\}. \tag{S70c}$$

Similarly, solving $\dot{\mathbf{x}} = \mathbf{0}$, we obtain equilibrium points, which can be divided into three categories. The first and second categories are single equilibrium points: a point on the D-vertex, $\mathbf{x}^{(D)} = (0,1,0)$, and a point on the DE-edge, $\mathbf{x}^{(DE)} = (0, x_2^{(DE)}, x_3^{(DE)})$, where

$$x_2^{(DE)} = \frac{1}{(k-2)(k+3)(\alpha+\beta)}\left[(k+1)\left(\frac{rc}{k+1} - c + k\beta\right) - 3(\alpha+\beta)\right], \tag{S71a}$$

$$x_3^{(DE)} = 1 - x_2^{(DE)} = \frac{1}{(k-2)(k+3)(\alpha+\beta)}\left[(k+1)\left(-\frac{rc}{k+1} + c + k\alpha\right) - 3(\alpha+\beta)\right]. \tag{S71b}$$

The third category contains infinite equilibrium points on the CE-edge, denoted by $\mathbf{x}^{(CE)} = (x_1^{(CE)}, 0, x_3^{(CE)})$, where $0 \le x_1^{(CE)} \le 1$, $0 \le x_3^{(CE)} \le 1$, $x_1^{(CE)} + x_3^{(CE)} = 1$. This category covers other two vertex points $(1,0,0)$ and $(0,0,1)$.

Again, the stability of $\mathbf{x}^{(D)}$ and $\mathbf{x}^{(DE)}$ can be studied by the regular method. We cancel $x_2 = 1 - x_1 - x_3$ and study the dynamics depicted by $\dot{x}_1$ and $\dot{x}_3$,

$$\dot{x}_1 = \frac{\delta(k-2)}{2(k-1)} x_1(1-x_1-x_3)\left\{ (k+1)\left(\frac{rc}{k+1}-c\right) + x_3(k-2)(k+3)(\alpha+\beta)\right\}, \tag{S72a}$$

$$\dot{x}_3 = \frac{\delta(k-2)}{2(k-1)} x_3(1-x_1-x_3)\left\{ (k+1)\left(\frac{rc}{k+1}-c-k\alpha\right) + x_3(k-2)(k+3)(\alpha+\beta) + 3(\alpha+\beta)\right\}. \tag{S72b}$$

The Jacobian matrix of system (S72) is

$$J = \begin{pmatrix} \dfrac{\partial \dot{x}_1}{\partial x_1} & \dfrac{\partial \dot{x}_1}{\partial x_3} \\ \dfrac{\partial \dot{x}_3}{\partial x_1} & \dfrac{\partial \dot{x}_3}{\partial x_3} \end{pmatrix}, \tag{S73}$$

where

$$\frac{\partial \dot{x}_1}{\partial x_1} = \frac{\delta(k-2)}{2(k-1)}(1-2x_1-x_3)\left\{ (k+1)\left(\frac{rc}{k+1}-c\right) + x_3(k-2)(k+3)(\alpha+\beta)\right\}, \tag{S74a}$$

$$\frac{\partial \dot{x}_1}{\partial x_3} = -\frac{\delta(k-2)}{2(k-1)} x_1\left\{ (k+1)\left(\frac{rc}{k+1}-c\right) - (1-x_1-2x_3)(k-2)(k+3)(\alpha+\beta)\right\}, \tag{S74b}$$

$$\frac{\partial \dot{x}_3}{\partial x_1} = -\frac{\delta(k-2)}{2(k-1)} x_3\left\{ (k+1)\left(\frac{rc}{k+1}-c-k\alpha\right) + x_3(k-2)(k+3)(\alpha+\beta) + 3(\alpha+\beta)\right\}, \tag{S74c}$$

$$\frac{\partial \dot{x}_3}{\partial x_3} = \frac{\delta(k-2)}{2(k-1)}\left\{ (1-x_1-2x_3)\left[(k+1)\left(\frac{rc}{k+1}-c-k\alpha\right) + 3(\alpha+\beta)\right] + x_3(2-2x_1-3x_3)(k-2)(k+3)(\alpha+\beta)\right\}. \tag{S74d}$$

Substituting the value of $\mathbf{x}^{(D)}$ into Eq. (S73), we have

$$J|_{\mathbf{x}=\mathbf{x}^{(D)}} = \frac{\delta(k-2)}{2(k-1)} \begin{pmatrix} (k+1)\left(\dfrac{rc}{k+1}-c\right) & 0 \\ 0 & (k+1)\left(\dfrac{rc}{k+1}-c-k\alpha\right) + 3(\alpha+\beta) \end{pmatrix}. \tag{S75}$$

The condition ensuring $J|_{\mathbf{x}=\mathbf{x}^{(D)}}$ negative-definite is $r < k+1$ and $r < (1+k\alpha/c)(k+1) - 3(\alpha+\beta)/c$. It is hard to compare them in size, but we can express the second condition as $x_3^{(DE)} > 0$, which holds if $\mathbf{x}^{(DE)}$ exists. Therefore, if $r < k+1$ and $\mathbf{x}^{(DE)}$ exists, the equilibrium $\mathbf{x}^{(D)}$ is stable.

Substitute the value of $\mathbf{x}^{(DE)}$ into Eq. (S73), we have $\partial \dot{x}_1/\partial x_3|_{\mathbf{x}=\mathbf{x}^{(DE)}} = 0$. Therefore, the first- and second-order sequential principal subformulas' negativity is equivalent to $\partial \dot{x}_1/\partial x_1|_{\mathbf{x}=\mathbf{x}^{(DE)}} < 0$ and $\partial \dot{x}_3/\partial x_3|_{\mathbf{x}=\mathbf{x}^{(DE)}} < 0$. We have

$$\left.\frac{\partial \dot{x}_1}{\partial x_1}\right|_{\mathbf{x}=\mathbf{x}^{(DE)}} = \frac{\delta(k-2)}{2(k-1)}(1-x_3^{(DE)})\left[(k+1)k\alpha - 3(\alpha+\beta)\right], \tag{S76}$$

whose sign is hard to judge. Instead, let us calculate

$$\left.\frac{\partial \dot{x}_3}{\partial x_3}\right|_{\mathbf{x}=\mathbf{x}^{(DE)}} = \frac{\delta(k-2)}{2(k-1)}(1-x_3^{(DE)})\left[(k+1)\left(-\frac{rc}{k+1}+c+k\alpha\right)-3(\alpha+\beta)\right]. \tag{S77}$$

We can see that as long as $0 < x_3^{(DE)} < 1$, we have $\partial \dot{x}_3/\partial x_3|_{\mathbf{x}=\mathbf{x}^{(DE)}} > 0$. Therefore, if $\mathbf{x}^{(DE)}$ exists, $\mathbf{x}^{(DE)}$ cannot be stable.

Next, we study the stability of $\mathbf{x}^{(CE)} = (x_1^{(CE)}, 0, x_3^{(CE)})$, which contains infinite equilibrium points satisfying $x_1^{(CE)} + x_3^{(CE)} = 1$. Similar to well-mixed populations, this is because when $x_2 = 0$, we have $\dot{x}_1 = \dot{x}_3$ and strategies 1 and 3 are indistinguishable. Concerning this, we can treat $x_1 + x_3$ as a whole and study the system depicted by $\dot{x}_2$ and $\dot{x}_1 + \dot{x}_3$. We can cancel $x_1 + x_3 = 1 - x_2$ and have

$$\dot{x}_2 = \frac{\delta(k-2)(k+1)}{2(k-1)}\left\{-x_2(1-x_2)\left(\frac{rc}{k+1}-c\right)+kx_2x_3\left(\frac{3-(k-2)(k+3)x_2}{k(k+1)}(\alpha+\beta)-\beta\right)\right\}. \tag{S78}$$

The element in the single-order Jacobian matrix is

$$\frac{\mathrm{d}\dot{x}_2}{\mathrm{d}x_2} = \frac{\delta(k-2)(k+1)}{2(k-1)}\left\{-(1-2x_2)\left(\frac{rc}{k+1}-c\right)+kx_3\left(\frac{3-2(k-2)(k+3)x_2}{k(k+1)}(\alpha+\beta)-\beta\right)\right\}. \tag{S79}$$

Substituting the value of $\mathbf{x}^{(CE)}$ into $\mathrm{d}\dot{x}_2/\mathrm{d}x_2$, we have

$$\left.\frac{\mathrm{d}\dot{x}_2}{\mathrm{d}x_2}\right|_{\mathbf{x}=\mathbf{x}^{(CE)}} = \frac{\delta(k-2)(k+1)}{2(k-1)}\left\{-\left(\frac{rc}{k+1}-c\right)-kx_3^{(CE)}\left(\frac{3}{k(k+1)}(\alpha+\beta)-\beta\right)\right\}. \tag{S80}$$

Therefore, $\mathbf{x}^{(CE)}$ is stable if $x_1^{(CE)} < x_{1,\star}^{(CE)}$ (or $x_3^{(CE)} > x_{3,\star}^{(CE)}$), where

$$x_{1,\star}^{(CE)} = 1 + \left(\frac{r}{k+1}-1\right)\frac{c}{k\beta - 3(\alpha+\beta)/(k+1)} \equiv \frac{(k-2)(k+3)(\alpha+\beta)}{k(k+1)\beta - 3(\alpha+\beta)}x_2^{(DE)}, \tag{S81}$$

or $x_{3,\star}^{(CE)} = \left(-\frac{r}{k+1}+1\right)\frac{c}{k\beta-3(\alpha+\beta)/(k+1)}$. That is, while every point on the CE-edge is equilibrium, only the points on the side of $x_1^{(CE)} < x_{1,\star}^{(CE)}$ are stable. Moreover, we note that $x_{1,\star}^{(CE)} > 1$ when $r > k+1$. That is, $x_1^{(CE)} < x_{1,\star}^{(CE)}$ always holds and the points on the CE-edge are stable everywhere for $r > k+1$. The conclusions here are based on a regular size of β. If the value of β is very small, then the conclusion may be reversed, which is a different phenomenon compared with the well-mixed population.

3.2.3 Discussion

According to stability analysis, we can divide the results into two cases. First, when $r > k+1$, the fixation of defection at $\mathbf{x}^{(D)}$ is unstable and the points on the CE-edge are stable everywhere. The dilemma is overcome through the principles known in the traditional public goods game.

Second, when $r < k+1$, the fixation of defection occurs in the traditional public goods game, but in this 3-strategy system with peer punishment, the situations can be different. Here, we discuss the effect of peer punishment when $r < k+1$ and show how the results in Fig. 4 in the main text are obtained.

As mentioned in the main text, when the punishment strength is intermediate ($\beta_0 < \beta < \beta^\star$ for structured populations and $\beta > \beta_0^{\mathrm{WM}}$ for well-mixed populations, as revealed later), there are a stable vertex equilibrium $\mathbf{x}^{(D)}$, an unstable edge equilibrium $\mathbf{x}^{(DE)}$, and a stable equilibrium line $\mathbf{x}^{(CE)}$. The vertex equilibrium $\mathbf{x}^{(D)}$ and the equilibrium line $\mathbf{x}^{(CE)}$ are bi-stable, depending on the initial state space divided by $\mathbf{x}^{(DE)}$ and $\mathbf{x}_\star^{(CE)} = (x_{1,\star}^{(CE)}, 0, x_{3,\star}^{(CE)})$. In well-mixed populations, $x_2^{(DE)} \to 1$ as $\beta \to \infty$ according to Eq. (S58a), and $\mathbf{x}^{(D)}$ is always stable regardless of the value of β according to Eq. (S61). This is the analytical illustration that peer punishment cannot truly resolve social dilemmas in a well-mixed population. However, in structured populations, $x_2^{(DE)} \to k(k+1)/[(k-2)(k+3)] > 1$ as $\beta \to \infty$ according to Eq. (S71a). In particular, $x_2^{(DE)} > 1$ happens (i.e., $\mathbf{x}^{(DE)}$ no longer exists) when

$$\frac{1}{(k-2)(k+3)(\alpha+\beta)}\left[(k+1)\left(\frac{rc}{k+1}-c+k\beta\right)-3(\alpha+\beta)\right] > 1$$

$$\Leftrightarrow \beta > \beta^{\star} \equiv \frac{k+1}{3}\left(-\frac{rc}{k+1}+c+k\alpha\right)-\alpha. \tag{S82}$$

Meanwhile, according to Eq. (S75), $\mathbf{x}^{(D)}$ becomes unstable at the same time when Eq. (S82) is satisfied. The bi-stable system state becomes mono-stable on the CE-edge. This is the analytical interpretation that peer punishment can resolve social dilemmas in structured populations.

On the other hand, structured populations do not always facilitate the advantage of peer punishment compared to well-mixed populations. This property can be observed from the critical punishment strength over which peer punishment starts to play a role. In a structured population, according to Eq. (S71a), $x_2^{(DE)} > 0$ means

$$\frac{1}{(k-2)(k+3)(\alpha+\beta)}\left[(k+1)\left(\frac{rc}{k+1}-c+k\beta\right)-3(\alpha+\beta)\right] > 0$$
$$\Leftrightarrow \beta > \beta_0 \equiv \frac{k+1}{k^2+k-3}\left(-\frac{rc}{k+1}+c\right)+\frac{3\alpha}{k^2+k-3}. \tag{S83}$$

And, according to Eq. (S81), $x_{1,\star}^{(CE)} > 0 \Leftrightarrow x_2^{(DE)} > 0$. When $\beta < \beta_0$, the system is mono-stable at the vertex equilibrium $\mathbf{x}^{(D)}$, as it is in the traditional public goods game. Peer punishment starts functioning and creates bi-stability in structured populations when $\beta > \beta_0$.

In a well-mixed population, according to Eq. (S58a), $x_2^{(DE)} > 0$ means

$$\frac{1}{k(\alpha+\beta)}\left(\frac{rc}{k+1}-c+k\beta\right) > 0 \Leftrightarrow \beta > \beta_0^{\mathrm{WM}} \equiv \frac{1}{k}\left(-\frac{rc}{k+1}+c\right), \tag{S84}$$

and $x_{1,\star}^{(CE)} > 0 \Leftrightarrow x_2^{(DE)} > 0$ according to Eq. (S67). Peer punishment starts working and creates bi-stability in well-mixed populations when $\beta > \beta_0^{\mathrm{WM}}$.

Comparing Eqs. (S83) and (S84), we see that $\beta_0^{\mathrm{WM}} < \beta_0$ always holds. That is, peer punishment first starts to play a role in well-mixed populations when increasing the punishment strength.

Actually, there is a considerable interval of β that $x_2^{(DE)}$ in structured populations are smaller than the ones in well-mixed populations. That is, a structured population enlarges the initial state space leading to full defection, thus less effective to utilize peer punishment. A structured population becomes more advantageous only when its $x_2^{(DE)}$ are greater than the ones in well-mixed populations. According to Eqs. (S58a) and (S71a), this means

$$\frac{1}{(k-2)(k+3)(\alpha+\beta)}\left[(k+1)\left(\frac{rc}{k+1}-c+k\beta\right)-3(\alpha+\beta)\right] > \frac{1}{k(\alpha+\beta)}\left(\frac{rc}{k+1}-c+k\beta\right)$$
$$\Leftrightarrow \beta > \beta_{=} \equiv \frac{2}{k}\left(-\frac{rc}{k+1}+c\right)+\alpha. \tag{S85}$$

Only when $\beta > \beta_{=}$, a structured population has a smaller initial state space leading to full defection, thus more effective to utilize peer punishment than well-mixed populations. In particular, $\beta > \beta^{\star}$ completely eliminates the initial state space leading to full defection as shown in Eq. (S82), while a well-mixed population cannot realize this.

3.3 Public goods games with pool punishment ($n = 3$)

As mentioned in the main text, there are $n = 3$ strategies in the public goods game with pool punishment[17].

$1 =$ Cooperation (C);

$2 =$ Defection (D);

$3 =$ Pool punishment (O);

Based on the traditional public goods game, the pool punishment strategy is introduced as an additional strategy. A punishing player always pays a cost α to establish the institution for pool punishment. A defective player is charged with a fine β if there is at least one punishing co-player. That is, given k_3 punishing co-players, a defective player has $\beta f(k_3)$ charged, where $f(k_3) = 1$ if $k_3 > 0$ and $f(k_3) = 0$ if $k_3 = 0$. Meanwhile, we assume punishing players also perform the cooperative behavior, investing c to the common pool. Again, this makes the cooperative players second-order free-riders.

Therefore, given the co-player configuration $\mathbf{k} = (k_1, k_2, k_3)$, we have the following payoff calculation in a single game.

$$a_{1|\mathbf{k}} = \frac{r(k_1+1+k_3)c}{k+1} - c = \frac{rc}{k+1}k_1 + \frac{rc}{k+1}k_3 + \frac{rc}{k+1} - c, \tag{S86a}$$

$$a_{2|\mathbf{k}} = \frac{r(k_1+k_3)c}{k+1} - \beta f(k_3) = \frac{rc}{k+1}k_1 + \frac{rc}{k+1}k_3 - \beta f(k_3), \tag{S86b}$$

$$a_{3|\mathbf{k}} = \frac{r(k_1+k_3+1)c}{k+1}k_1 - c - \alpha = \frac{rc}{k+1}k_1 + \frac{rc}{k+1}k_3 + \frac{rc}{k+1} - c - \alpha. \tag{S86c}$$

3.3.1 The well-mixed population

The frequencies of strategies 1, 2, 3 are denoted by x_1, x_2, and x_3 (or x_C, x_D, and x_O in the main text), respectively. In a well-mixed population, the mean payoffs of the three strategies are calculated as follows.

$$\bar{\pi}_1 = \sum_{k_1+k_2+k_3=k} \frac{k!}{k_1!k_2!k_3!} {x_1}^{k_1} {x_2}^{k_2} {x_3}^{k_3} a_{1|\mathbf{k}} = \frac{rc}{k+1}kx_1 + \frac{rc}{k+1}kx_3 + \frac{rc}{k+1} - c, \tag{S87a}$$

$$\bar{\pi}_2 = \sum_{k_1+k_2+k_3=k} \frac{k!}{k_1!k_2!k_3!} {x_1}^{k_1} {x_2}^{k_2} {x_3}^{k_3} a_{2|\mathbf{k}} = \frac{rc}{k+1}kx_1 + \frac{rc}{k+1}kx_3 - \beta\left[1-(1-x_3)^k\right], \tag{S87b}$$

$$\bar{\pi}_3 = \sum_{k_1+k_2+k_3=k} \frac{k!}{k_1!k_2!k_3!} {x_1}^{k_1} {x_2}^{k_2} {x_3}^{k_3} a_{3|\mathbf{k}} = \frac{rc}{k+1}kx_1 + \frac{rc}{k+1}kx_3 + \frac{rc}{k+1} - c - \alpha. \tag{S87c}$$

The mean payoff of the total population is then calculated by

$$\bar{\pi} = x_1\bar{\pi}_1 + x_2\bar{\pi}_2 + x_3\bar{\pi}_3 = \frac{rc}{k+1}kx_1 + \frac{rc}{k+1}kx_3 + (x_1+x_3)\left(\frac{rc}{k+1} - c\right) - x_3\alpha - x_2\left[1-(1-x_3)^k\right]. \tag{S88}$$

On this basis, we can write the replicator equations of the well-mixed population $\dot{x}_i = x_i(\bar{\pi}_i - \bar{\pi})$ as follows.

$$\dot{x}_1 = x_1(\bar{\pi}_1 - \bar{\pi}) = x_1\left\{(1-x_1-x_3)\left(\frac{rc}{k+1} - c\right) + x_3\alpha + x_2\beta\left[1-(1-x_3)^k\right]\right\}, \tag{S89a}$$

$$\dot{x}_2 = x_2(\bar{\pi}_2 - \bar{\pi}) = x_2\left\{-(x_1+x_3)\left(\frac{rc}{k+1} - c\right) + x_3\alpha - (1-x_2)\beta\left[1-(1-x_3)^k\right]\right\}, \tag{S89b}$$

$$\dot{x}_3 = x_3(\bar{\pi}_3 - \bar{\pi}) = x_3\left\{(1-x_1-x_3)\left(\frac{rc}{k+1} - c\right) - (1-x_3)\alpha + x_2\beta\left[1-(1-x_3)^k\right]\right\}. \tag{S89c}$$

We denote the system state $\mathbf{x} = (x_1, x_2, x_3)$. Solving $\dot{\mathbf{x}} = \mathbf{0}$, we obtain four possible equilibrium points: a point on the C-vertex, $\mathbf{x}^{(C)} = (1,0,0)$, a point on the D-vertex, $\mathbf{x}^{(D)} = (0,1,0)$, a point on the O-vertex, $\mathbf{x}^{(O)} = (0,0,1)$, and a point on the DO-edge, $\mathbf{x}^{(DO)} = (0, x_2^{(DO)}, x_3^{(DO)})$, where

$$x_2^{(DO)} = \sqrt[k]{1 + \frac{1}{\beta}\left(\frac{rc}{k+1} - c - \alpha\right)}, \tag{S90a}$$

$$x_3^{(DO)} = 1 - x_2^{(DO)} = 1 - \sqrt[k]{1 + \frac{1}{\beta}\left(\frac{rc}{k+1} - c - \alpha\right)}. \tag{S90b}$$

The stability of these four equilibrium points can be studied by the regular method. We cancel $x_2 = 1 - x_1 - x_3$ and study the dynamics depicted by $\dot{x}_1$ and $\dot{x}_3$,

$$\dot{x}_1 = x_1\left\{(1-x_1-x_3)\left(\frac{rc}{k+1} - c\right) + x_3\alpha + (1-x_1-x_3)\left[1-(1-x_3)^k\right]\right\}, \tag{S91a}$$

$$\dot{x}_3 = x_3\left\{(1-x_1-x_3)\left(\frac{rc}{k+1} - c\right) - (1-x_3)\alpha + (1-x_1-x_3)\left[1-(1-x_3)^k\right]\right\}. \tag{S91b}$$

The Jacobian matrix of system (S91) is

$$J = \begin{pmatrix} \dfrac{\partial \dot{x}_1}{\partial x_1} & \dfrac{\partial \dot{x}_1}{\partial x_3} \\ \dfrac{\partial \dot{x}_3}{\partial x_1} & \dfrac{\partial \dot{x}_3}{\partial x_3} \end{pmatrix}, \tag{S92}$$

where

$$\frac{\partial \dot{x}_1}{\partial x_1} = (1-2x_1-x_3)\left(\frac{rc}{k+1}-c\right)+x_3\alpha+(1-2x_1-x_3)\left[1-(1-x_3)^k\right], \tag{S93}$$

$$\frac{\partial \dot{x}_1}{\partial x_3} = x_1\left\{-\frac{rc}{k+1}+c+\alpha-\beta+(1-x_3)^{k-1}\beta\left[1-x_3+k(1-x_1-x_3)\right]\right\}, \tag{S94}$$

$$\frac{\partial \dot{x}_3}{\partial x_1} = -x_3\left\{\frac{rc}{k+1}-c+\beta\left[1-(1-x_3)^k\right]\right\}, \tag{S95}$$

$$\begin{aligned}\frac{\partial \dot{x}_3}{\partial x_3} &= (1-x_1-2x_3)\left(\frac{rc}{k+1}-c\right)-(1-2x_3)\alpha \\ &\quad +\beta\left\{1-x_1-2x_3+(1-x_3)^{k-1}\left[-(1-x_1-x_3)+(k+2)(1-x_3)x_3-(k+1)x_1x_3\right]\right\}.\end{aligned} \tag{S96}$$

Substituting the value of $\mathbf{x}^{(C)}$ into Eq. (S92), we have

$$J|_{\mathbf{x}=\mathbf{x}^{(C)}} = \begin{pmatrix} -\frac{rc}{k+1}+c & -\frac{rc}{k+1}+c+\alpha-\beta \\ 0 & -\alpha \end{pmatrix}. \tag{S97}$$

The condition ensuring $J|_{\mathbf{x}=\mathbf{x}^{(C)}}$ negative-definite is $r>k+1$. Therefore, the C-vertex equilibrium $\mathbf{x}^{(C)}$ is stable if and only if $r>k+1$.

Substituting the value of $\mathbf{x}^{(D)}$ into Eq. (S92), we have

$$J|_{\mathbf{x}=\mathbf{x}^{(D)}} = \begin{pmatrix} \frac{rc}{k+1}-c & 0 \\ 0 & \frac{rc}{k+1}-c-\alpha \end{pmatrix}. \tag{S98}$$

The condition ensuring $J|_{\mathbf{x}=\mathbf{x}^{(D)}}$ negative-definite is $r<k+1$. Therefore, the D-vertex equilibrium $\mathbf{x}^{(D)}$ is stable if and only if $r<k+1$.

Substituting the value of $\mathbf{x}^{(O)}$ into Eq. (S92), we have $\partial \dot{x}_1/\partial x_1|_{\mathbf{x}=\mathbf{x}^{(O)}} = \alpha > 0$. Therefore, the Jacobian matrix at $\mathbf{x}^{(O)}$ is not negative-definite and the O-vertex equilibrium $\mathbf{x}^{(O)}$ is not stable.

Substituting the value of $\mathbf{x}^{(DO)}$ into Eq. (S92), we note that

$$\left.\frac{\partial \dot{x}_1}{\partial x_1}\right|_{\mathbf{x}=\mathbf{x}^{(DO)}} = \sqrt[k]{1+\frac{1}{\beta}\left(\frac{rc}{k+1}-c-\alpha\right)}\left\{\left(\frac{rc}{k+1}-c-\alpha\right)+\beta\left[-\frac{1}{\beta}\left(\frac{rc}{k+1}-c-\alpha\right)\right]\right\}+\alpha = \alpha > 0. \tag{S99}$$

Therefore, the Jacobian matrix at $\mathbf{x}^{(DO)}$ is not negative-definite and the equilibrium $\mathbf{x}^{(DO)}$ on the DO-edge is not stable.

3.3.2 The structured population

Next, we study pool punishment in structured populations. We notice that the payoff structure given by Eq. (S86) is nonlinear. Therefore, to obtain the replicator dynamics in a structured population, we need to utilize Eq. (S26) given by Supplementary Note 2.4.2. The process is to calculate all elements of the '$\langle a_{i|\mathbf{k}_{+j}}\rangle_i$ type' and the '$\langle a_{i|\mathbf{k}_{+j}}\rangle_j$ type' manually, which is tedious. For this $n=3$ system, the $\langle a_{i|\mathbf{k}_{+j}}\rangle_i$ type is

$$\left[\langle a_{i|\mathbf{k}_{+j}}\rangle_i\right]_{ij} = \begin{pmatrix} \langle a_{1|\mathbf{k}_{+1}}\rangle_1 & \langle a_{1|\mathbf{k}_{+2}}\rangle_1 & \langle a_{1|\mathbf{k}_{+3}}\rangle_1 \\ \langle a_{2|\mathbf{k}_{+1}}\rangle_2 & \langle a_{2|\mathbf{k}_{+2}}\rangle_2 & \langle a_{2|\mathbf{k}_{+3}}\rangle_2 \\ \langle a_{3|\mathbf{k}_{+1}}\rangle_3 & \langle a_{3|\mathbf{k}_{+2}}\rangle_3 & \langle a_{3|\mathbf{k}_{+3}}\rangle_3 \end{pmatrix}, \tag{S100}$$

where, by applying Eq. (S86) to Eq. (S21), we have

$$\begin{aligned}\langle a_{1|\mathbf{k}_{+1}}\rangle_1 &= \sum_{k_1+k_2+k_3=k-1}\frac{(k-1)!}{k_1!k_2!k_3!}q_{1|1}^{k_1}q_{2|1}^{k_2}q_{3|1}^{k_3}a_{1|\mathbf{k}_{+1}} \\ &= \sum_{k_1+k_2+k_3=k-1}\frac{(k-1)!}{k_1!k_2!k_3!}q_{1|1}^{k_1}q_{2|1}^{k_2}q_{3|1}^{k_3}\left(\frac{rc}{k+1}(k_1+1)+\frac{rc}{k+1}k_3+\frac{rc}{k+1}-c\right)\end{aligned}$$

$$= \frac{rc}{k+1}(k-1)q_{1|1} + \frac{rc}{k+1}(k-1)q_{3|1} + \frac{2rc}{k+1} - c$$

$$= \frac{rc}{k+1}(k-2)x_1 + \frac{rc}{k+1}(k-2)x_3 + \frac{3rc}{k+1} - c, \tag{S101}$$

and similarly,

$$\langle a_{1|\mathbf{k}_{+2}}\rangle_1 = \frac{rc}{k+1}(k-2)x_1 + \frac{rc}{k+1}(k-2)x_3 + \frac{2rc}{k+1} - c, \tag{S102a}$$

$$\langle a_{1|\mathbf{k}_{+3}}\rangle_1 = \frac{rc}{k+1}(k-2)x_1 + \frac{rc}{k+1}(k-2)x_3 + \frac{3rc}{k+1} - c; \tag{S102b}$$

$$\langle a_{2|\mathbf{k}_{+1}}\rangle_2 = \frac{rc}{k+1}(k-2)x_1 + \frac{rc}{k+1}(k-2)x_3 + \frac{rc}{k+1} - \beta\left[1 - \left(1 - \frac{k-2}{k-1}x_3\right)^{k-1}\right], \tag{S102c}$$

$$\langle a_{2|\mathbf{k}_{+2}}\rangle_2 = \frac{rc}{k+1}(k-2)x_1 + \frac{rc}{k+1}(k-2)x_3 - \beta\left[1 - \left(1 - \frac{k-2}{k-1}x_3\right)^{k-1}\right], \tag{S102d}$$

$$\langle a_{2|\mathbf{k}_{+3}}\rangle_2 = \frac{rc}{k+1}(k-2)x_1 + \frac{rc}{k+1}(k-2)x_3 + \frac{rc}{k+1} - \beta; \tag{S102e}$$

$$\langle a_{3|\mathbf{k}_{+1}}\rangle_3 = \frac{rc}{k+1}(k-2)x_1 + \frac{rc}{k+1}(k-2)x_3 + \frac{3rc}{k+1} - c - \alpha, \tag{S102f}$$

$$\langle a_{3|\mathbf{k}_{+2}}\rangle_3 = \frac{rc}{k+1}(k-2)x_1 + \frac{rc}{k+1}(k-2)x_3 + \frac{2rc}{k+1} - c - \alpha, \tag{S102g}$$

$$\langle a_{3|\mathbf{k}_{+3}}\rangle_3 = \frac{rc}{k+1}(k-2)x_1 + \frac{rc}{k+1}(k-2)x_3 + \frac{3rc}{k+1} - c - \alpha. \tag{S102h}$$

Next, the $\langle a_{i|\mathbf{k}_{+j}}\rangle_j$ type is

$$\left[\langle a_{i|\mathbf{k}_{+j}}\rangle_j\right]_{ij} = \begin{pmatrix} \langle a_{1|\mathbf{k}_{+1}}\rangle_1 & \langle a_{1|\mathbf{k}_{+2}}\rangle_2 & \langle a_{1|\mathbf{k}_{+3}}\rangle_3 \\ \langle a_{2|\mathbf{k}_{+1}}\rangle_1 & \langle a_{2|\mathbf{k}_{+2}}\rangle_2 & \langle a_{2|\mathbf{k}_{+3}}\rangle_3 \\ \langle a_{3|\mathbf{k}_{+1}}\rangle_1 & \langle a_{3|\mathbf{k}_{+2}}\rangle_2 & \langle a_{3|\mathbf{k}_{+3}}\rangle_3 \end{pmatrix}, \tag{S103}$$

where the diagonal elements have been calculated previously, and the remaining elements are

$$\langle a_{1|\mathbf{k}_{+2}}\rangle_2 = \frac{rc}{k+1}(k-2)x_1 + \frac{rc}{k+1}(k-2)x_3 + \frac{2rc}{k+1} - c, \tag{S104a}$$

$$\langle a_{1|\mathbf{k}_{+3}}\rangle_3 = \frac{rc}{k+1}(k-2)x_1 + \frac{rc}{k+1}(k-2)x_3 + \frac{3rc}{k+1} - c; \tag{S104b}$$

$$\langle a_{2|\mathbf{k}_{+1}}\rangle_1 = \frac{rc}{k+1}(k-2)x_1 + \frac{rc}{k+1}(k-2)x_3 + \frac{2rc}{k+1} - \beta\left[1 - \left(1 - \frac{k-2}{k-1}x_3\right)^{k-1}\right], \tag{S104c}$$

$$\langle a_{2|\mathbf{k}_{+3}}\rangle_3 = \frac{rc}{k+1}(k-2)x_1 + \frac{rc}{k+1}(k-2)x_3 + \frac{2rc}{k+1} - \beta; \tag{S104d}$$

$$\langle a_{3|\mathbf{k}_{+1}}\rangle_1 = \frac{rc}{k+1}(k-2)x_1 + \frac{rc}{k+1}(k-2)x_3 + \frac{3rc}{k+1} - c - \alpha, \tag{S104e}$$

$$\langle a_{3|\mathbf{k}_{+2}}\rangle_2 = \frac{rc}{k+1}(k-2)x_1 + \frac{rc}{k+1}(k-2)x_3 + \frac{rc}{k+1} - c - \alpha. \tag{S104f}$$

According to Eqs. (S101), (S102a)–(S102h), and (S104a)–(S104f), there are totaling $(2n-1)n = 15$ elements in these two '$\langle a_{i|\mathbf{k}_{+j}}\rangle_i$' and '$\langle a_{i|\mathbf{k}_{+j}}\rangle_j$' types.

We can apply these elements to Eq. (S26), which is

$$\dot{x}_i = \frac{\delta(k-2)}{2(k-1)}x_i\sum_{j=1}^{3}x_j\left(\langle a_{i|\mathbf{k}_{+j}}\rangle_i + (k-1)\langle a_{i|\mathbf{k}_{+j}}\rangle_j + \langle a_{i|\mathbf{k}_{+i}}\rangle_i - \langle a_{j|\mathbf{k}_{+i}}\rangle_j - \langle a_{j|\mathbf{k}_{+i}}\rangle_i - (k-2)\sum_{j=1}^{n}x_l\langle a_{j|\mathbf{k}_{+l}}\rangle_l - \langle a_{j|\mathbf{k}_{+j}}\rangle_j\right). \tag{S105}$$

Let us do this step by step. For $i = 1$, we have

$$\sum_{j=1}^{3}x_j\langle a_{1|\mathbf{k}_{+j}}\rangle_1 = \frac{rc}{k+1}(k-1)x_1 + \frac{rc}{k+1}(k-1)x_3 + \frac{2rc}{k+1} - c, \tag{S106a}$$

$$\sum_{j=1}^{3} x_j \langle a_{1|\mathbf{k}_{+j}} \rangle_j = \frac{rc}{k+1} k x_1 + \frac{rc}{k+1} k x_3 + \frac{rc}{k+1} - c, \tag{S106b}$$

$$\sum_{j=1}^{3} x_j \langle a_{1|\mathbf{k}_{+1}} \rangle_1 = \frac{rc}{k+1}(k-2)x_1 + \frac{rc}{k+1}(k-2)x_3 + \frac{3rc}{k+1} - c, \tag{S106c}$$

$$\sum_{j=1}^{3} x_j \langle a_{j|\mathbf{k}_{+1}} \rangle_1 = \frac{rc}{k+1}(k-1)x_1 + \frac{rc}{k+1}(k-1)x_3 + \frac{2rc}{k+1} - c(x_1+x_3) - \alpha x_3 - \beta(1-x_1-x_3)\left[1-\left(1-\frac{k-2}{k-1}x_3\right)^{k-1}\right], \tag{S106d}$$

$$\sum_{j=1}^{3} x_j \langle a_{j|\mathbf{k}_{+1}} \rangle_j = \frac{rc}{k+1} k x_1 + \frac{rc}{k+1} k x_3 + \frac{rc}{k+1} - c(x_1+x_3) - \alpha x_3 - \beta(1-x_1-x_3)\left[1-\left(1-\frac{k-2}{k-1}x_3\right)^{k-1}\right], \tag{S106e}$$

$$\sum_{j=1}^{3} x_j \sum_{l=1}^{3} x_l \langle a_{j|\mathbf{k}_{+l}} \rangle_l = (rc-c)(x_1+x_3) - \alpha x_3 - \beta(1-x_1-x_3)(1-x_3)\left[1-\left(1-\frac{k-2}{k-1}x_3\right)^{k-1}\right] - \beta(1-x_1-x_3)x_3, \tag{S106f}$$

$$\sum_{j=1}^{3} x_j \langle a_{j|\mathbf{k}_{+j}} \rangle_j = (rc-c)(x_1+x_3) - \alpha x_3 - \beta(1-x_1-x_3)\left[1-\left(1-\frac{k-2}{k-1}x_3\right)^{k-1}\right], \tag{S106g}$$

which can be used to obtain $\dot{x}_1$.

For $i=3$, $\sum_{j=1}^{3} x_j \sum_{l=1}^{3} x_l \langle a_{j|\mathbf{k}_{+l}} \rangle_l$ and $\sum_{j=1}^{3} x_j \langle a_{j|\mathbf{k}_{+j}} \rangle_j$ have been obtained previously, and we have the remaining terms:

$$\sum_{j=1}^{3} x_j \langle a_{3|\mathbf{k}_{+j}} \rangle_3 = \frac{rc}{k+1}(k-1)x_1 + \frac{rc}{k+1}(k-1)x_3 + \frac{2rc}{k+1} - c - \alpha, \tag{S107a}$$

$$\sum_{j=1}^{3} x_j \langle a_{3|\mathbf{k}_{+j}} \rangle_j = \frac{rc}{k+1} k x_1 + \frac{rc}{k+1} k x_3 + \frac{rc}{k+1} - c - \alpha, \tag{S107b}$$

$$\sum_{j=1}^{3} x_j \langle a_{3|\mathbf{k}_{+3}} \rangle_3 = \frac{rc}{k+1}(k-2)x_1 + \frac{rc}{k+1}(k-2)x_3 + \frac{3rc}{k+1} - c - \alpha, \tag{S107c}$$

$$\sum_{j=1}^{3} x_j \langle a_{j|\mathbf{k}_{+3}} \rangle_3 = \frac{rc}{k+1}(k-1)x_1 + \frac{rc}{k+1}(k-1)x_3 + \frac{2rc}{k+1} - c(x_1+x_3) - \alpha x_3 - \beta(1-x_1-x_3), \tag{S107d}$$

$$\sum_{j=1}^{3} x_j \langle a_{j|\mathbf{k}_{+3}} \rangle_j = \frac{rc}{k+1} k x_1 + \frac{rc}{k+1} k x_3 + \frac{rc}{k+1} - c(x_1+x_3) - \alpha x_3 - \beta(1-x_1-x_3), \tag{S107e}$$

which can be used to obtain $\dot{x}_3$.

Applying Eqs. (S106a)–(S106g) and (S107a)–(S107e) to Eq. (S105), we obtain the following simple replicator equation:

$$\dot{x}_1 = \frac{\delta(k-2)(k+1)}{2(k-1)} x_1 \left\{ (1-x_1-x_3)\left(\frac{rc}{k+1} - c\right) + x_3\alpha + (1-x_1-x_3)\beta\left[1-\left(1-\frac{k-2}{k+1}x_3\right)\left(1-\frac{k-2}{k-1}x_3\right)^{k-1}\right]\right\}, \tag{S108a}$$

$$\dot{x}_3 = \frac{\delta(k-2)(k+1)}{2(k-1)} x_3 \left\{ (1-x_1-x_3)\left(\frac{rc}{k+1} - c\right) - (1-x_3)\alpha + (1-x_1-x_3)\beta\left[1-\frac{k-1}{k+1}\left(1-\frac{k-2}{k-1}x_3\right)^{k}\right]\right\}. \tag{S108b}$$

Please note that $\dot{x}_2 = -\dot{x}_1 - \dot{x}_3$ has been canceled.

Similarly, solving $\dot{\mathbf{x}} = \mathbf{0}$, we obtain four possible equilibrium points: a point on the C-vertex, $\mathbf{x}^{(C)} = (1,0,0)$, a point on the D-vertex, $\mathbf{x}^{(D)} = (0,1,0)$, a point on the O-vertex, $\mathbf{x}^{(O)} = (0,0,1)$, and a point on the DO-edge, $\mathbf{x}^{(DO)} = (0, x_2^{(DO)}, x_3^{(DO)})$, where

$$x_2^{(DO)} = \frac{k-1}{k-2}\left(-\frac{1}{k-1} + \sqrt[k]{\frac{k+1}{k-1}\left[1+\frac{1}{\beta}\left(\frac{rc}{k+1} - c - \alpha\right)\right]}\right), \tag{S109a}$$

$$x_3^{(DO)} = 1 - x_2^{(DO)} = \frac{k-1}{k-2}\left(1 - \sqrt[k]{\frac{k+1}{k-1}\left[1 + \frac{1}{\beta}\left(\frac{rc}{k+1} - c - \alpha\right)\right]}\right). \tag{S109b}$$

The Jacobian matrix of system (S108) is

$$J = \begin{pmatrix} \frac{\partial \dot{x}_1}{\partial x_1} & \frac{\partial \dot{x}_1}{\partial x_3} \\ \frac{\partial \dot{x}_3}{\partial x_1} & \frac{\partial \dot{x}_3}{\partial x_3} \end{pmatrix}, \tag{S110}$$

where

$$\frac{\partial \dot{x}_1}{\partial x_1} = \frac{\delta(k-2)(k+1)}{2(k-1)}\left\{(1-2x_1-x_3)\left(\frac{rc}{k+1} - c\right) + x_3\alpha \right.$$
$$\left. + (1-2x_1-x_3)\beta\left[1 - \left(1 - \frac{k-2}{k+1}x_3\right)\left(1 - \frac{k-2}{k-1}x_3\right)^{k-1}\right]\right\}, \tag{S111}$$

$$\frac{\partial \dot{x}_1}{\partial x_3} = \frac{\delta(k-2)(k+1)}{2(k-1)}x_1\left\{-\frac{rc}{k+1} + c + \alpha - \beta\left[1 - \left(1 - \frac{k-2}{k+1}x_3\right)\left(1 - \frac{k-2}{k-1}x_3\right)^{k-1}\right]\right.$$
$$\left. + (1-x_1-x_3)\beta\left(1 - \frac{k-2}{k-1}x_3\right)^{k-2}\frac{k-2}{k+1}\left(k+2 - \frac{k(k-2)}{k-1}x_3\right)\right\}, \tag{S112}$$

$$\frac{\partial \dot{x}_3}{\partial x_1} = -\frac{\delta(k-2)(k+1)}{2(k-1)}x_3\left\{\frac{rc}{k+1} - c + \beta\left[1 - \frac{k-1}{k+1}\left(1 - \frac{k-2}{k-1}x_3\right)^k\right]\right\}, \tag{S113}$$

$$\frac{\partial \dot{x}_3}{\partial x_3} = \frac{\delta(k-2)(k+1)}{2(k-1)}\left\{(1-x_1-2x_3)\left(\frac{rc}{k+1} - c\right) - (1-2x_3)\alpha + (1-x_1-2x_3)\beta\left[1 - \frac{k-1}{k+1}\left(1 - \frac{k-2}{k-1}x_3\right)^k\right]\right.$$
$$\left. + x_3(1-x_1-x_3)\beta\frac{k(k-2)}{k+1}\left(1 - \frac{k-2}{k-1}x_3\right)^{k-1}\right\}. \tag{S114}$$

Substituting the value of $\mathbf{x}^{(C)}$ into Eq. (S110), we have

$$J|_{\mathbf{x}=\mathbf{x}^{(C)}} = \frac{\delta(k-2)(k+1)}{2(k-1)}\begin{pmatrix} -\frac{rc}{k+1} + c & -\frac{rc}{k+1} + c + \alpha \\ 0 & -\frac{rc}{k+1} + c - \alpha \end{pmatrix}. \tag{S115}$$

The condition ensuring $J|_{\mathbf{x}=\mathbf{x}^{(C)}}$ negative-definite is $r > k+1$. Therefore, the C-vertex equilibrium $\mathbf{x}^{(D)}$ is stable if and only if $r > k+1$.

Substituting the value of $\mathbf{x}^{(D)}$ into Eq. (S110), we have

$$J|_{\mathbf{x}=\mathbf{x}^{(D)}} = \frac{\delta(k-2)(k+1)}{2(k-1)}\begin{pmatrix} \frac{rc}{k+1} - c & 0 \\ 0 & \frac{rc}{k+1} - c - \alpha + \frac{2\beta}{k+1} \end{pmatrix}. \tag{S116}$$

The condition ensuring $J|_{\mathbf{x}=\mathbf{x}^{(D)}}$ negative-definite and the D-vertex equilibrium $\mathbf{x}^{(D)}$ stable is $r < k+1$ and $\beta < \beta^\star$, where

$$\beta^\star = \frac{k+1}{2}\left(-\frac{rc}{k+1} + c + \alpha\right). \tag{S117}$$

This indicates that when $\beta > \beta^\star$, the D-vertex equilibrium cannot be stable even if $r < k+1$.

Substituting the value of $\mathbf{x}^{(O)}$ into Eq. (S110), we have $\partial \dot{x}_1 / \partial x_1|_{\mathbf{x}=\mathbf{x}^{(O)}} \propto \alpha > 0$. Therefore, the Jacobian matrix at $\mathbf{x}^{(O)}$ is not negative-definite and the O-vertex equilibrium $\mathbf{x}^{(O)}$ is not stable.

Substituting the value of $\mathbf{x}^{(DO)}$ into Eq. (S110), we note that $\partial\dot{x}_3/\partial x_1|_{\mathbf{x}=\mathbf{x}^{(DO)}} = 0$. Therefore, the first- and second-order sequential principal subformulas' negativity is equivalent to $\partial\dot{x}_1/\partial x_1|_{\mathbf{x}=\mathbf{x}^{(DO)}} < 0$ and $\partial\dot{x}_3/\partial x_3|_{\mathbf{x}=\mathbf{x}^{(DO)}} < 0$. Let us calculate

$$\begin{aligned}\left.\frac{\partial\dot{x}_3}{\partial x_3}\right|_{\mathbf{x}=\mathbf{x}^{(DO)}} &= \frac{\delta(k-2)(k+1)}{2(k-1)}\left\{(1-2x_3^{(DO)})\left(\frac{rc}{k+1}-c-\alpha+\beta\left[1-\frac{k-1}{k+1}\left(1-\frac{k-2}{k-1}x_3^{(DO)}\right)^k\right]\right)\right.\\ &\quad\left.+x_3^{(DO)}(1-x_3^{(DO)})\beta\frac{k(k-2)}{k+1}\left(1-\frac{k-2}{k-1}x_3^{(DO)}\right)^{k-1}\right\}\\ &= \frac{\delta(k-2)(k+1)}{2(k-1)}x_3^{(DO)}(1-x_3^{(DO)})\beta\frac{k(k-2)}{k+1}\left(1-\frac{k-2}{k-1}x_3^{(DO)}\right)^{k-1} > 0.\end{aligned} \tag{S118}$$

Therefore, the equilibrium $\mathbf{x}^{(DO)}$ on the DO-edge is not stable.

3.3.3 Discussion

According to stability analysis, we can divide the results into two cases. First, when $r > k+1$, the fixation of defection at $\mathbf{x}^{(D)}$ is unstable and the fixation of cooperation at $\mathbf{x}^{(C)}$ is stable. The dilemma is overcome through known principles known in the traditional public goods game.

Second, when $r < k+1$, the fixation of defection occurs in the traditional public goods game, but in this 3-strategy system with pool punishment, the situations can be different. Here, we discuss the effect of pool punishment when $r < k+1$ and show how the results in Fig. 7 in the main text are obtained.

As mentioned in the main text, when the punishment strength is intermediate ($\beta_0 < \beta < \beta^\star$ for structured populations and $\beta > \beta_0^{\mathrm{WM}}$ for well-mixed populations, as revealed later), there are a stable vertex equilibrium $\mathbf{x}^{(D)}$, an unstable vertex equilibrium $\mathbf{x}^{(O)}$, an unstable vertex equilibrium $\mathbf{x}^{(C)}$, and an unstable edge equilibrium $\mathbf{x}^{(DO)}$. In the 3-strategy system state space, $\mathbf{x}^{(D)}$ is the only stable equilibrium point. However, on the DO edge where $x_1 = 0$, the vertex equilibrium points $\mathbf{x}^{(D)}$ and $\mathbf{x}^{(O)}$ are bi-stable, depending on the initial state divided by $\mathbf{x}^{(DO)}$. In well-mixed populations, $x_2^{(DO)} \to 1$ as $\beta \to \infty$ according to Eq. (S90a), and $\mathbf{x}^{(D)}$ is always stable regardless of the value of β according to Eq. (S98). This is the analytical illustration that pool punishment cannot truly resolve social dilemmas in a well-mixed population. However, in structured populations, $x_2^{(DO)} \to [(k+1)^{1/k}(k-1)^{1-1/k}-1]/(k-2) > 1$ as $\beta \to \infty$ according to Eq. (S109a). In particular, $x_2^{(DO)} > 1$ happens (i.e., $\mathbf{x}^{(DO)}$ no longer exists) when

$$\frac{k-1}{k-2}\left(-\frac{1}{k-1}+\sqrt[k]{\frac{k+1}{k-1}\left[1+\frac{1}{\beta}\left(\frac{rc}{k+1}-c-\alpha\right)\right]}\right) > 1 \Leftrightarrow \beta > \beta^\star \equiv \frac{k+1}{2}\left(-\frac{rc}{k+1}+c+\alpha\right). \tag{S119}$$

Meanwhile, according to Eq. (S116), $\mathbf{x}^{(D)}$ becomes unstable at the same time when Eq. (S119) is satisfied, while $\mathbf{x}^{(O)}$ and $\mathbf{x}^{(C)}$ remain unstable. In this case, the system enters a rock-paper-scissors cycle, where defection conquers cooperation ($\partial\dot{x}_1/\partial x_1 < 0$ when $x_3 = 0$), cooperation conquers pool punishment ($\partial\dot{x}_1/\partial x_1 > 0$ when $x_2 = 0$), and pool punishment conquers defection ($\partial\dot{x}_3/\partial x_3 > 0$ when $x_1 = 0$). This is the analytical interpretation that pool punishment can resolve social dilemmas in structured populations.

On the other hand, we also find that structured populations do not always facilitate the advantage of pool punishment compared to well-mixed populations. This property can be observed from the critical punishment strength over which pool punishment starts to play a role. In a structured population, according to Eq. (S109a), $x_2^{(DO)} > 0$ means

$$\begin{aligned}&\frac{k-1}{k-2}\left(-\frac{1}{k-1}+\sqrt[k]{\frac{k+1}{k-1}\left[1+\frac{1}{\beta}\left(\frac{rc}{k+1}-c-\alpha\right)\right]}\right) > 0\\ &\Leftrightarrow \beta > \beta_0 \equiv \frac{(k+1)(k-1)^{k-1}}{1-(k+1)(k-1)^{k-1}}\left(\frac{rc}{k+1}-c-\alpha\right).\end{aligned} \tag{S120}$$

When $\beta < \beta_0$, $\mathbf{x}^{(DO)}$ is not a real vector and the system is stable at the vertex equilibrium $\mathbf{x}^{(D)}$, even along the DO-edge. Pool punishment starts working and creates bi-stability along the DO-edge in structured populations when $\beta > \beta_0$.

In a well-mixed population, according to Eq. (S90a), $x_2^{(DO)} > 0$ means

$$\sqrt[k]{1+\frac{1}{\beta}\left(\frac{rc}{k+1}-c-\alpha\right)} > 0 \Leftrightarrow \beta > \beta_0^{\mathrm{WM}} \equiv -\frac{rc}{k+1}+c+\alpha. \tag{S121}$$

Pool punishment starts functioning and creates bi-stability along the DO-edge in well-mixed populations when $\beta > \beta_0^{\mathrm{WM}}$.

Comparing Eqs. (S120) and (S121), we see that $\beta_0^{\mathrm{WM}} < \beta_0$ always holds. That is, pool punishment first becomes efficient in well-mixed populations when increasing the punishment strength.

Actually, there is a considerable interval of β that $x_2^{(DO)}$ in structured populations are smaller than the ones in well-mixed populations. That is, a structured population enlarges the initial state interval leading to full defection along the DO-edge, thus less effective to utilize pool punishment. A structured population becomes more advantageous only when its $x_2^{(DO)}$ are greater than the ones in well-mixed populations. According to Eqs. (S90a) and (S109a), this means

$$
\begin{aligned}
&\frac{k-1}{k-2}\left(-\frac{1}{k-1}+\sqrt[k]{\frac{k+1}{k-1}\left[1+\frac{1}{\beta}\left(\frac{rc}{k+1}-c-\alpha\right)\right]}\right) > \sqrt[k]{1+\frac{1}{\beta}\left(\frac{rc}{k+1}-c-\alpha\right)} \\
\Leftrightarrow\ &\beta > \beta_{=} \equiv \frac{\left[(k+1)^{\frac{1}{k}}(k-1)^{1-\frac{1}{k}}-k+2\right]^k}{1-\left[(k+1)^{\frac{1}{k}}(k-1)^{1-\frac{1}{k}}-k+2\right]^k}\left(\frac{rc}{k+1}-c-\alpha\right). \qquad \text{(S122)}
\end{aligned}
$$

Only when $\beta > \beta_{=}$, a structured population has a smaller initial state interval leading to full defection along the DO-edge, thus more effective to utilize pool punishment than well-mixed populations. In particular, $\beta > \beta^{\star}$ can even destabilize the full defection equilibrium as shown in Eq. (S119) and transform the system to a rock-paper-scissors-like cyclic dominance, while a well-mixed population cannot realize this.

3.4 Public goods games with the reward mechanism ($n = 3$)

As a different mechanism from punishment, we study public goods games with the rewarding mechanism. In this model, there are $n = 3$ strategies[18]:

1 = Cooperation (C);
2 = Defection (D);
3 = Reward (R).

On the basis of the public goods game, a rewarding player, as the third strategy, pays a normalized cost α/k to reward a co-player who cooperates. An individual with cooperative behavior receives a normalized reward γ/k. Here, the cost α and reward γ are normalized to keep comparable with the previous work[18]. We assume that rewarding players also perform the cooperative behavior (i.e., investing c in the common pool). Therefore, there are $k_1 + k_3$ co-players who perform the cooperative behavior, which incurs the cost $\alpha(k_1 + k_3)/k$ to each rewarding player. Given k_3 rewarding co-players, each cooperator receives a reward $\gamma k_3/k$. Each rewarding player, as performing the cooperative behavior, also receives a reward $\gamma k_3/k$.

To sum up, given the co-player configuration $\mathbf{k} = (k_1, k_2, k_3)$, we have the following payoff calculation in a single game.

$$
a_{1|\mathbf{k}} = \frac{r(k_1+1+k_3)c}{k+1} - c + \frac{\gamma k_3}{k} = \frac{rc}{k+1}k_1 + \left(\frac{rc}{k+1}+\frac{\gamma}{k}\right)k_3 + \frac{rc}{k+1} - c, \qquad \text{(S123a)}
$$

$$
a_{2|\mathbf{k}} = \frac{r(k_1+k_3)c}{k+1} = \frac{rc}{k+1}k_1 + \frac{rc}{k+1}k_3, \qquad \text{(S123b)}
$$

$$
a_{3|\mathbf{k}} = \frac{r(k_1+k_3+1)c}{k+1} - c + \frac{\gamma k_3}{k} - \frac{\alpha(k_1+k_3)}{k} = \left(\frac{rc}{k+1}-\frac{\alpha}{k}\right)k_1 + \left(\frac{rc}{k+1}+\frac{\gamma-\alpha}{k}\right)k_3 + \frac{rc}{k+1} - c. \qquad \text{(S123c)}
$$

3.4.1 The well-mixed population

The frequencies of strategies 1, 2, 3 are denoted by x_1, x_2, and x_3 (or x_C, x_D, and x_R for straightforward understanding), respectively. In a well-mixed population, the mean payoffs of the three strategies are calculated as follows.

$$
\bar{\pi}_1 = \sum_{k_1+k_2+k_3=k} \frac{k!}{k_1!k_2!k_3!} {x_1}^{k_1}{x_2}^{k_2}{x_3}^{k_3} a_{1|\mathbf{k}} = \frac{rc}{k+1}kx_1 + \left(\frac{rc}{k+1}+\frac{\gamma}{k}\right)kx_3 + \frac{rc}{k+1} - c, \qquad \text{(S124a)}
$$

$$
\bar{\pi}_2 = \sum_{k_1+k_2+k_3=k} \frac{k!}{k_1!k_2!k_3!} {x_1}^{k_1}{x_2}^{k_2}{x_3}^{k_3} a_{2|\mathbf{k}} = \frac{rc}{k+1}kx_1 + \frac{rc}{k+1}kx_3, \qquad \text{(S124b)}
$$

$$
\bar{\pi}_3 = \sum_{k_1+k_2+k_3=k} \frac{k!}{k_1!k_2!k_3!} {x_1}^{k_1}{x_2}^{k_2}{x_3}^{k_3} a_{3|\mathbf{k}} = \left(\frac{rc}{k+1}-\frac{\alpha}{k}\right)kx_1 + \left(\frac{rc}{k+1}+\frac{\gamma-\alpha}{k}\right)kx_3 + \frac{rc}{k+1} - c. \qquad \text{(S124c)}
$$

The mean payoff of the total population is then calculated by

$$
\bar{\pi} = x_1\bar{\pi}_1 + x_2\bar{\pi}_2 + x_3\bar{\pi}_3 = \frac{rc}{k+1}kx_1 + \frac{rc}{k+1}kx_3 + (x_1+x_3)\left(\frac{rc}{k+1}-c\right) + x_3(x_1+x_3)(\gamma-\alpha). \qquad \text{(S125)}
$$

On this basis, we can write the replicator equations of the well-mixed population $\dot{x}_i = x_i(\bar{\pi}_i - \bar{\pi})$ as follows.

$$\dot{x}_1 = x_1(\bar{\pi}_1 - \bar{\pi}) = x_1\left[(1-x_1-x_3)\left(\frac{rc}{k+1}-c\right)+x_3(1-x_1-x_3)\gamma+x_3(x_1+x_3)\alpha\right], \tag{S126a}$$

$$\dot{x}_2 = x_2(\bar{\pi}_2 - \bar{\pi}) = x_2\left[-(x_1+x_3)\left(\frac{rc}{k+1}-c\right)-x_3(x_1+x_3)\gamma+x_3(x_1+x_3)\alpha\right], \tag{S126b}$$

$$\dot{x}_3 = x_3(\bar{\pi}_3 - \bar{\pi}) = x_3\left[(1-x_1-x_3)\left(\frac{rc}{k+1}-c\right)+x_3(1-x_1-x_3)\gamma-(1-x_3)(x_1+x_3)\alpha\right]. \tag{S126c}$$

We denote the system state $\mathbf{x} = (x_1, x_2, x_3)$. Solving $\dot{\mathbf{x}} = \mathbf{0}$, we obtain four equilibrium points: a point on the C-vertex, $\mathbf{x}^{(C)} = (1,0,0)$, a point on the D-vertex, $\mathbf{x}^{(D)} = (0,1,0)$, a point on the R-vertex, $\mathbf{x}^{(R)} = (0,0,1)$, and a point on the DR-edge, $\mathbf{x}^{(DR)} = (0, x_2^{(DR)}, x_3^{(DR)})$, where

$$x_2^{(DR)} = 1 - \frac{1}{\gamma-\alpha}\left(-\frac{rc}{k+1}+c\right), \tag{S127a}$$

$$x_3^{(DR)} = \frac{1}{\gamma-\alpha}\left(-\frac{rc}{k+1}+c\right). \tag{S127b}$$

The stability of these four equilibrium points can be studied using the regular method. We cancel $x_2 = 1 - x_1 - x_3$ and study the dynamics depicted by $\dot{x}_1$ and $\dot{x}_3$,

$$\dot{x}_1 = x_1\left[(1-x_1-x_3)\left(\frac{rc}{k+1}-c\right)+x_3(1-x_1-x_3)\gamma+x_3(x_1+x_3)\alpha\right], \tag{S128a}$$

$$\dot{x}_3 = x_3\left[(1-x_1-x_3)\left(\frac{rc}{k+1}-c\right)+x_3(1-x_1-x_3)\gamma-(1-x_3)(x_1+x_3)\alpha\right]. \tag{S128b}$$

The Jacobian matrix of system (S128) is

$$J = \begin{pmatrix} \dfrac{\partial \dot{x}_1}{\partial x_1} & \dfrac{\partial \dot{x}_1}{\partial x_3} \\ \dfrac{\partial \dot{x}_3}{\partial x_1} & \dfrac{\partial \dot{x}_3}{\partial x_3} \end{pmatrix}, \tag{S129}$$

where

$$\frac{\partial \dot{x}_1}{\partial x_1} = (1-2x_1-x_3)\left(\frac{rc}{k+1}-c+x_3\gamma\right)+x_3(2x_1+x_3)\alpha, \tag{S130a}$$

$$\frac{\partial \dot{x}_1}{\partial x_3} = x_1\left(-\frac{rc}{k+1}+c+(1-x_1-2x_3)\gamma+(x_1+2x_3)\alpha\right), \tag{S130b}$$

$$\frac{\partial \dot{x}_3}{\partial x_1} = x_3\left(-\frac{rc}{k+1}+c-x_3\gamma-(1-x_3)\alpha\right), \tag{S130c}$$

$$\frac{\partial \dot{x}_3}{\partial x_3} = (1-x_1-2x_3)\left(\frac{rc}{k+1}-c\right)+x_3(2-2x_1-3x_3)\gamma-(x_1+2x_3)\alpha+x_3(2x_1+3x_3)\alpha. \tag{S130d}$$

Substituting the value of $\mathbf{x}^{(C)}$ into Eq. (S129), we have

$$J|_{\mathbf{x}=\mathbf{x}^{(C)}} = \begin{pmatrix} -\dfrac{rc}{k+1}+c & -\dfrac{rc}{k+1}+c+\alpha \\ 0 & -\alpha \end{pmatrix}. \tag{S131}$$

The conditions ensuring $J|_{\mathbf{x}=\mathbf{x}^{(C)}}$ negative-definite are $r > k+1$ and $\alpha > 0$. The second condition holds clearly. Therefore, we know that $\mathbf{x}^{(C)}$ is stable if and only if $r > k+1$.

Substituting the value of $\mathbf{x}^{(D)}$ into Eq. (S129), we have

$$J|_{\mathbf{x}=\mathbf{x}^{(D)}} = \begin{pmatrix} \dfrac{rc}{k+1}-c & 0 \\ 0 & \dfrac{rc}{k+1}-c \end{pmatrix}. \tag{S132}$$

The condition ensuring $J|_{\mathbf{x}=\mathbf{x}^{(D)}}$ negative-definite is $r<k+1$. Therefore, the equilibrium $\mathbf{x}^{(D)}$ is stable when $r<k+1$.

Substituting the value of $\mathbf{x}^{(R)}$ into Eq. (S129), we have

$$J|_{\mathbf{x}=\mathbf{x}^{(R)}} = \begin{pmatrix} \alpha & 0 \\ -\frac{rc}{k+1}+c-\gamma & -\frac{rc}{k+1}+c-\gamma+\alpha \end{pmatrix}. \tag{S133}$$

Since $\alpha>0$, $J|_{\mathbf{x}=\mathbf{x}^{(R)}}$ is not negative-definite. Therefore, the equilibrium $\mathbf{x}^{(R)}$ is not stable.

Substituting the value of $\mathbf{x}^{(DR)}$ into Eq. (S129), we obtain $J|_{\mathbf{x}=\mathbf{x}^{(DR)}}$, in which

$$\begin{aligned} \left.\frac{\partial \dot{x}_1}{\partial x_1}\right|_{\mathbf{x}=\mathbf{x}^{(DR)}} &= (1-x_3^{(DR)})\left(\frac{rc}{k+1}-c\right)+x_3^{(DR)}\left(\gamma+x_3^{(DR)}(\gamma-\alpha)\right) \\ &= \left[\alpha+\frac{2}{\gamma-\alpha}\left(-\frac{rc}{k+1}+c\right)\right]\left(-\frac{rc}{k+1}+c\right)\frac{1}{\gamma-\alpha}>0. \end{aligned} \tag{S134}$$

Eq. (S134) holds because $\gamma-\alpha>0$ as long as $x_3^{(DR)}$ exists according to Eq. (S127b), and the case that we focus on is $r<k+1$. Therefore, the equilibrium point $\mathbf{x}^{(DR)}$ is not stable.

3.4.2 The structured population

We notice that the payoff structure given by Eq. (S123) is linear, which means that we can utilize the simplified method for special linear systems given by Supplementary Note 2.4.3 for convenience.

Comparing the payoff structure $a_{i|\mathbf{k}}$ in Eq. (S123) with Eqs. (S29)–(S31), we extract matrices $\mathbf{b}$ and $\mathbf{c}$,

$$\mathbf{b} = \begin{pmatrix} \frac{rc}{k+1} & 0 & \frac{rc}{k+1}+\frac{\gamma}{k} \\ \frac{rc}{k+1} & 0 & \frac{rc}{k+1} \\ \frac{rc}{k+1}-\frac{\alpha}{k} & 0 & \frac{rc}{k+1}+\frac{\gamma-\alpha}{k} \end{pmatrix}, \quad \mathbf{c} = \begin{pmatrix} \frac{rc}{k+1}-c \\ 0 \\ \frac{rc}{k+1}-c \end{pmatrix}. \tag{S135}$$

According to Eq. (S35), let us calculate

$$3\sum_{j=1}^{3} x_j(b_{11}-b_{1j}-b_{j1}-b_{jj}) = -6(x_1+x_3)\frac{rc}{k+1}-6x_3\frac{\gamma-\alpha}{k}, \tag{S136a}$$

$$3\sum_{j=1}^{3} x_j(b_{22}-b_{2j}-b_{j2}-b_{jj}) = -6(x_1+x_3)\frac{rc}{k+1}-3x_3\frac{\gamma-\alpha}{k}, \tag{S136b}$$

$$3\sum_{j=1}^{3} x_j(b_{33}-b_{3j}-b_{j3}-b_{jj}) = -6(x_1+x_3)\frac{rc}{k+1}+(3x_2-6x_3)\frac{\gamma-\alpha}{k}, \tag{S136c}$$

$$6\sum_{j=1}^{3}\sum_{l=1}^{3} x_j x_l b_{jl} = 6(x_1+x_3)\frac{rc}{k+1}+6x_3(x_1+x_3)\frac{\gamma-\alpha}{k}. \tag{S136d}$$

Then, we insert them into Eq. (S35). Meanwhile, the expressions of $\bar{\pi}_i$ and $\bar{\pi}$ are the same as we give in Supplementary Note 3.4.1 for well-mixed populations. In this way, we obtain the replicator equations in structured populations as follows.

$$\dot{x}_1 = \frac{\delta(k-2)}{2(k-1)}x_1\left\{(k+1)\left[(1-x_1-x_3)\left(\frac{rc}{k+1}-c\right)+x_3(1-x_1-x_3)\gamma+x_3(x_1+x_3)\alpha\right]-6x_2x_3\frac{\gamma-\alpha}{k}\right\}, \tag{S137a}$$

$$\dot{x}_2 = \frac{\delta(k-2)}{2(k-1)}x_2\left\{(k+1)\left[-(x_1+x_3)\left(\frac{rc}{k+1}-c\right)-x_3(x_1+x_3)\gamma+x_3(x_1+x_3)\alpha\right]-3x_3(1-2x_1-2x_3)\frac{\gamma-\alpha}{k}\right\}, \tag{S137b}$$

$$\dot{x}_3 = \frac{\delta(k-2)}{2(k-1)}x_3\left\{(k+1)\left[(1-x_1-x_3)\left(\frac{rc}{k+1}-c\right)+x_3(1-x_1-x_3)\gamma-(1-x_3)(x_1+x_3)\alpha\right]+3x_2(1-2x_3)\frac{\gamma-\alpha}{k}\right\}. \tag{S137c}$$

Similarly, solving $\dot{\mathbf{x}} = \mathbf{0}$, we can obtain five possible equilibrium points: a point on the C-vertex, $\mathbf{x}^{(C)} = (1,0,0)$, a point on the D-vertex, $\mathbf{x}^{(D)} = (0,1,0)$, a point on the R-vertex, $\mathbf{x}^{(R)} = (0,0,1)$, a point on the DR-edge, $\mathbf{x}^{(DR)} = (0, x_2^{(DR)}, x_3^{(DR)})$, where

$$x_2^{(DR)} = \frac{k(k+1)}{(k^2+k-6)(\gamma-\alpha)}\left(\frac{rc}{k+1} - c\right) + \frac{k^2+k-3}{k^2+k-6}, \tag{S138a}$$

$$x_3^{(DR)} = \frac{k(k+1)}{(k^2+k-6)(\gamma-\alpha)}\left(-\frac{rc}{k+1} + c\right) - \frac{3}{k^2+k-6}, \tag{S138b}$$

and finally, an interior point $\mathbf{x}^{(CDR)} = (x_1^{(CDR)}, x_2^{(CDR)}, x_3^{(CDR)})$, where

$$x_1^{(CDR)} = 1 - \frac{k(k+1)\alpha}{k(k+1)\alpha + 3(\gamma-\alpha)} - \frac{k(k+1)}{k(k+1)\gamma - 3(\gamma-\alpha)}\left(-\frac{rc}{k+1} + c\right), \tag{S139a}$$

$$x_2^{(CDR)} = \frac{k(k+1)\alpha}{k(k+1)\alpha + 3(\gamma-\alpha)}, \tag{S139b}$$

$$x_3^{(CDR)} = \frac{k(k+1)}{k(k+1)\gamma - 3(\gamma-\alpha)}\left(-\frac{rc}{k+1} + c\right). \tag{S139c}$$

We note that in public goods games with the reward mechanism, structured populations create a new equilibrium point $\mathbf{x}^{(CDR)}$ that does not exist in well-mixed populations. This is a new phenomenon that we did not observe in punishment mechanisms.

We cancel $x_2 = 1 - x_1 - x_3$ and study the dynamics depicted by $\dot{x}_1$ and $\dot{x}_3$,

$$\dot{x}_1 = \frac{\delta(k-2)(k+1)}{2(k-1)} x_1 \left\{ (1-x_1-x_3)\left[\frac{rc}{k+1} - c + x_3\left(\gamma - 6\frac{\gamma-\alpha}{k(k+1)}\right)\right] + x_3(x_1+x_3)\alpha \right\}, \tag{S140a}$$

$$\dot{x}_3 = \frac{\delta(k-2)(k+1)}{2(k-1)} x_3 \left\{ x_3(1-x_1-x_3)\left[\frac{rc}{k+1} - c + x_3\left(\gamma - 6\frac{\gamma-\alpha}{k(k+1)}\right) + 3\frac{\gamma-\alpha}{k(k+1)}\right] - (1-x_3)(x_1+x_3)\alpha \right\}. \tag{S140b}$$

The Jacobian matrix of system (S140) is

$$J = \begin{pmatrix} \dfrac{\partial \dot{x}_1}{\partial x_1} & \dfrac{\partial \dot{x}_1}{\partial x_3} \\ \dfrac{\partial \dot{x}_3}{\partial x_1} & \dfrac{\partial \dot{x}_3}{\partial x_3} \end{pmatrix}, \tag{S141}$$

where

$$\frac{\partial \dot{x}_1}{\partial x_1} = \frac{\delta(k-2)(k+1)}{2(k-1)} \left\{ (1-2x_1-x_3)\left[\frac{rc}{k+1} - c + x_3\left(\gamma - 6\frac{\gamma-\alpha}{k(k+1)}\right)\right] + x_3(2x_1+x_3)\alpha \right\}, \tag{S142a}$$

$$\frac{\partial \dot{x}_1}{\partial x_3} = \frac{\delta(k-2)(k+1)}{2(k-1)} x_1 \left[-\frac{rc}{k+1} + c - 2x_3\left(\gamma - 6\frac{\gamma-\alpha}{k(k+1)}\right) + (x_1+2x_3)\alpha\right], \tag{S142b}$$

$$\frac{\partial \dot{x}_3}{\partial x_1} = \frac{\delta(k-2)(k+1)}{2(k-1)} x_3 \left[-\frac{rc}{k+1} + c - x_3\left(\gamma - 6\frac{\gamma-\alpha}{k(k+1)}\right) - 3\frac{\gamma-\alpha}{k(k+1)} - (1-x_3)\alpha\right], \tag{S142c}$$

$$\begin{aligned}\frac{\partial \dot{x}_3}{\partial x_3} = \frac{\delta(k-2)(k+1)}{2(k-1)} \Bigg\{ &(1-x_1-2x_3)\left(\frac{rc}{k+1} - c + 3\frac{\gamma-\alpha}{k(k+1)}\right) + (2-2x_1-3x_3)x_3\left(\gamma - 6\frac{\gamma-\alpha}{k(k+1)}\right) \\ &- (1-2x_3)x_1\alpha - (2-3x_3)x_3\alpha \Bigg\}. \end{aligned} \tag{S142d}$$

Substituting the value of $\mathbf{x}^{(C)}$ into Eq. (S141), we have

$$J|_{\mathbf{x}=\mathbf{x}^{(C)}} = \frac{\delta(k-2)(k+1)}{2(k-1)} \begin{pmatrix} -\dfrac{rc}{k+1} + c & -\dfrac{rc}{k+1} + c + \alpha \\ 0 & -\alpha \end{pmatrix}. \tag{S143}$$

The conditions ensuring $J|_{\mathbf{x}=\mathbf{x}^{(C)}}$ negative-definite are $r > k+1$ and $\alpha > 0$. The second condition holds clearly. Therefore, we know that $\mathbf{x}^{(C)}$ is stable if and only if $r > k+1$.

Substituting the value of $\mathbf{x}^{(D)}$ into Eq. (S141), we have

$$J|_{\mathbf{x}=\mathbf{x}^{(D)}} = \frac{\delta(k-2)(k+1)}{2(k-1)} \begin{pmatrix} \frac{rc}{k+1} - c & 0 \\ 0 & \frac{rc}{k+1} - c + 3\frac{\gamma-\alpha}{k(k+1)} \end{pmatrix}. \tag{S144}$$

The conditions ensuring $J|_{\mathbf{x}=\mathbf{x}^{(D)}}$ negative-definite and the D-vertex equilibrium $\mathbf{x}^{(D)}$ stable are $r < k+1$ and $\gamma < \gamma^{\star}$, where

$$\gamma^{\star} = \frac{k(k+1)}{3}\left(-\frac{rc}{k+1} + c\right) + \alpha. \tag{S145}$$

This indicates that when $\gamma > \gamma^{\star}$, the D-vertex equilibrium cannot be stable even if $r < k+1$.

Substituting the value of $\mathbf{x}^{(R)}$ into Eq. (S141), we have

$$J|_{\mathbf{x}=\mathbf{x}^{(R)}} = \frac{\delta(k-2)(k+1)}{2(k-1)} \begin{pmatrix} \alpha & 0 \\ -\frac{rc}{k+1} + c - \gamma - 3\frac{\gamma-\alpha}{k(k+1)} & -\frac{rc}{k+1} + c - \frac{k^2+k-3}{k(k+1)}(\gamma-\alpha) \end{pmatrix}. \tag{S146}$$

Since $\alpha > 0$, $J|_{\mathbf{x}=\mathbf{x}^{(R)}}$ is not negative-definite. Therefore, the R-vertex equilibrium point $\mathbf{x}^{(R)}$ is not stable.

Substituting the value of $\mathbf{x}^{(DR)}$ into Eq. (S141), we obtain $J|_{\mathbf{x}=\mathbf{x}^{(DR)}}$, where we note that $\partial\dot{x}_1/\partial x_3|_{\mathbf{x}=\mathbf{x}^{(DR)}} = 0$. Therefore, the first- and second-order sequential principal subformulas' negativity is equivalent to $\partial\dot{x}_1/\partial x_1|_{\mathbf{x}=\mathbf{x}^{(DR)}} < 0$ and $\partial\dot{x}_3/\partial x_3|_{\mathbf{x}=\mathbf{x}^{(DR)}} < 0$. Let us study $\partial\dot{x}_3/\partial x_3|_{\mathbf{x}=\mathbf{x}^{(DR)}}$. Reorganizing Eq. (S142d) but keeping $x_3^{(DR)}$, we obtain

$$\left.\frac{\partial\dot{x}_3}{\partial x_3}\right|_{\mathbf{x}=\mathbf{x}^{(DR)}} = \frac{\delta(k-2)(k+1)}{2(k-1)}\left(\mathscr{A}(x_3^{(DR)})^2 + \mathscr{B}x_3^{(DR)} + \mathscr{C}\right), \tag{S147}$$

where $\mathscr{A}$, $\mathscr{B}$, and $\mathscr{C}$ are constants,

$$\mathscr{A} = -3\frac{k^2+k-6}{k(k+1)}(\gamma-\alpha), \tag{S148a}$$

$$\mathscr{B} = 2\left(-\frac{rc}{k+1} + c + \frac{k^2+k-9}{k(k+1)}(\gamma-\alpha)\right), \tag{S148b}$$

$$\mathscr{C} = \frac{rc}{k+1} - c + 3\frac{\gamma-\alpha}{k(k+1)}. \tag{S148c}$$

Importantly, we notice that

$$x_3^{(DR)} < 1 \Leftrightarrow \gamma - \alpha > \frac{k(k+1)}{k^2+k-3}\left(-\frac{rc}{k+1} + c\right) > 0, \tag{S149}$$

which indicates $\mathscr{A} < 0$.

According to Eq. (S147), $\partial\dot{x}_3/\partial x_3|_{\mathbf{x}=\mathbf{x}^{(DR)}}$ is a quadratic function of $x_3^{(DR)}$, with $\mathscr{A} < 0$ as we found. The properties of quadratic functions indicate that $\partial\dot{x}_3/\partial x_3|_{\mathbf{x}=\mathbf{x}^{(DR)}} \geq 0$ in the interval $0 \leq x_3^{(DR)} \leq 1$ as long as (1) $\partial\dot{x}_3/\partial x_3|_{\mathbf{x}=\mathbf{x}^{(DR)}} \geq 0$ at $x_3^{(DR)} = 0$ and (2) $\partial\dot{x}_3/\partial x_3|_{\mathbf{x}=\mathbf{x}^{(DR)}} \geq 0$ at $x_3^{(DR)} = 1$. Let us examine these two conditions. First, when $x_3^{(DR)} = 0$, we have

$$\left.\frac{\partial\dot{x}_3}{\partial x_3}\right|_{\mathbf{x}=\mathbf{x}^{(DR)}} = \frac{rc}{k+1} - c + 3\frac{\gamma-\alpha}{k(k+1)} = -\frac{k(k+1)}{(k^2+k-6)(\gamma-\alpha)}x_3^{(DR)} = 0. \tag{S150}$$

Second, when $x_3^{(DR)} = 1$, we have

$$\left.\frac{\partial\dot{x}_3}{\partial x_3}\right|_{\mathbf{x}=\mathbf{x}^{(DR)}} = -\frac{rc}{k+1} + c - \frac{k^2+k-3}{k(k+1)}(\gamma-\alpha) = -\frac{k(k+1)}{(k^2+k-6)(\gamma-\alpha)}(1-x_3^{(DR)}) = 0. \tag{S151}$$

Therefore, $\partial\dot{x}_3/\partial x_3|_{\mathbf{x}=\mathbf{x}^{(DR)}} \geq 0$ and $J|_{\mathbf{x}=\mathbf{x}^{(DR)}}$ is not negative-definite in the interval $0 \leq x_3^{(DR)} \leq 1$. The equilibrium point $\mathbf{x}^{(DR)}$ is not stable when it exists.

Substituting the value of $\mathbf{x}^{(CDR)}$ into Eq. (S141), we obtain $J|_{\mathbf{x}=\mathbf{x}^{(CDR)}}$, where

$$\left.\frac{\partial \dot{x}_1}{\partial x_1}\right|_{\mathbf{x}=\mathbf{x}^{(CDR)}} = \frac{\delta(k-2)(k+1)}{2(k-1)}\Bigg\{(1-2x_1^{(CDR)}-x_3^{(CDR)})\left[\frac{rc}{k+1}-c+x_3^{(CDR)}\left(\gamma-6\frac{\gamma-\alpha}{k(k+1)}\right)\right] + x_3^{(CDR)}(2x_1^{(CDR)}+x_3^{(CDR)})\alpha\Bigg\}. \tag{S152}$$

At $\mathbf{x}^{(CDR)}$, we have $\dot{x}_1 = 0$, that is,

$$\frac{\delta(k-2)(k+1)}{2(k-1)}\left\{(1-x_1^{(CDR)}-x_3^{(CDR)})\left[\frac{rc}{k+1}-c+x_3^{(CDR)}\left(\gamma-6\frac{\gamma-\alpha}{k(k+1)}\right)\right]+x_3^{(CDR)}(x_1^{(CDR)}+x_3^{(CDR)})\alpha\right\}=0. \tag{S153}$$

We calculate Eq. (S152) minus Eq. (S153), (i.e., $\partial\dot{x}_3/\partial x_3|_{\mathbf{x}=\mathbf{x}^{(CDR)}} - 0 = \partial\dot{x}_3/\partial x_3|_{\mathbf{x}=\mathbf{x}^{(CDR)}}$) and further organize the result, which leads to

$$\begin{aligned}\left.\frac{\partial \dot{x}_1}{\partial x_1}\right|_{\mathbf{x}=\mathbf{x}^{(CDR)}} &= \frac{\delta(k-2)(k+1)}{2(k-1)}x_1^{(CDR)}\left(-\frac{rc}{k+1}+c-x_3^{(CDR)}\frac{k^2+k-6}{k(k+1)}(\gamma-\alpha)\right)\\ &= \frac{\delta(k-2)(k+1)}{2(k-1)}x_1^{(CDR)}\frac{k(k+1)\alpha+3(\gamma-\alpha)}{k(k+1)\gamma-3(\gamma-\alpha)}\left(-\frac{rc}{k+1}+c\right)\\ &= \frac{\delta(k-2)(k+1)}{2(k-1)}x_1^{(CDR)}\frac{x_3^{(CDR)}}{x_2^{(CDR)}}\alpha>0.\end{aligned} \tag{S154}$$

Therefore, $J|_{\mathbf{x}=\mathbf{x}^{(CDR)}}$ is not negative-definite and the interior equilibrium point $\mathbf{x}^{(CDR)}$ is not stable when it exists.

3.4.3 Discussion

Similar to the case in punishment, according to stability analysis, we can divide the results into two cases. First, when $r > k+1$, the fixation of defection at $\mathbf{x}^{(D)}$ is unstable, and the fixation of cooperation at $\mathbf{x}^{(C)}$ is stable. The dilemma is overcome through known principles known in the traditional public goods game.

Second, when $r < k+1$, the fixation of defection occurs in the traditional public goods game, but in this 3-strategy system with the reward mechanism, the situations can be different. Here, we discuss the effect of reward when $r < k+1$.

When the reward strength is intermediate ($\gamma_0 < \gamma < \gamma^\star$ for structured populations and $\gamma > \gamma_0^{\mathrm{WM}}$ for well-mixed populations, as revealed later), there are a stable vertex equilibrium $\mathbf{x}^{(D)}$, an unstable vertex equilibrium $\mathbf{x}^{(R)}$, an unstable vertex equilibrium $\mathbf{x}^{(C)}$, an unstable edge equilibrium $\mathbf{x}^{(DR)}$, and for structured populations, a possible unstable interior equilibrium $\mathbf{x}^{(CDR)}$. In the 3-strategy system state space, $\mathbf{x}^{(D)}$ is the only stable equilibrium point. However, on the *DR* edge where $x_1 = 0$, the vertex equilibrium points $\mathbf{x}^{(D)}$ and $\mathbf{x}^{(R)}$ are bi-stable, depending on the initial state divided by $\mathbf{x}^{(DR)}$. In well-mixed populations, $x_2^{(DR)} \to 1$ as $\gamma \to \infty$ according to Eq. (S127a), and $\mathbf{x}^{(D)}$ is always stable regardless of the value of γ according to Eq. (S132). In this way, we analytically illustrate that reward cannot truly resolve social dilemmas in a well-mixed population, similar to punishment. However, in structured populations, $x_2^{(DR)} \to (k^2+k-3)/(k^2+k-6) > 1$ as $\gamma \to \infty$ according to Eq. (S138a). In particular, $x_2^{(DR)} > 1$ happens (i.e., $\mathbf{x}^{(DR)}$ no longer exists) when

$$\frac{k(k+1)}{(k^2+k-6)(\gamma-\alpha)}\left(\frac{rc}{k+1}-c\right)+\frac{k^2+k-3}{k^2+k-6}>1 \Leftrightarrow \gamma>\gamma^\star\equiv\frac{k(k+1)}{3}\left(-\frac{rc}{k+1}+c\right)+\alpha. \tag{S155}$$

Meanwhile, according to Eq. (S144), $\mathbf{x}^{(D)}$ becomes unstable at the same time when Eq. (S155) is satisfied, while $\mathbf{x}^{(R)}$ and $\mathbf{x}^{(C)}$ remain unstable. In this case, the system enters a rock-paper-scissors cycle (Fig. S1**c**, **d**), where defection conquers cooperation ($\partial\dot{x}_1/\partial x_1 < 0$ when $x_3 = 0$), cooperation conquers reward ($\partial\dot{x}_1/\partial x_1 > 0$ when $x_2 = 0$), and reward conquers defection ($\partial\dot{x}_3/\partial x_3 > 0$ when $x_1 = 0$). In this way, we analytically confirm that reward can resolve social dilemmas in structured populations (Fig. S1**e**).

On the other hand, we also find that structured populations do not always facilitate the advantage of reward compared to well-mixed populations. This property can be observed from the critical reward strength over which the reward mechanism starts to play a role. In a structured population, according to Eq. (S138a), $x_2^{(DR)} > 0$ means

$$\frac{k(k+1)}{(k^2+k-6)(\gamma-\alpha)}\left(\frac{rc}{k+1}-c\right)+\frac{k^2+k-3}{k^2+k-6}>0 \Leftrightarrow \gamma>\gamma_0\equiv\frac{k(k+1)}{k^2+k-3}\left(-\frac{rc}{k+1}+c\right)+\alpha. \tag{S156}$$

When $\gamma < \gamma$, we have $x_2^{(DR)} < 0$ and the system is stable at the vertex equilibrium $\mathbf{x}^{(D)}$ even along the DR-edge. The reward mechanism starts working and creates bi-stability along the DR-edge in structured populations when $\gamma > \gamma_0$.

In a well-mixed population, according to Eq. (S127a), $x_2^{(DR)} > 0$ means

$$1 - \frac{1}{\gamma - \alpha}\left(-\frac{rc}{k+1} + c\right) > 0 \Leftrightarrow \gamma > \gamma_0^{\mathrm{WM}} \equiv -\frac{rc}{k+1} + c + \alpha. \tag{S157}$$

The reward mechanism starts functioning and creates bi-stability along the DR-edge in well-mixed populations when $\gamma > \gamma_0^{\mathrm{WM}}$.

Comparing Eqs. (S156) and (S157), we see that $\gamma_0^{\mathrm{WM}} < \gamma_0$ always holds. That is, the reward mechanism first becomes efficient in well-mixed populations when increasing the reward strength.

There is a considerable interval of γ that $x_2^{(DR)}$ in structured populations are smaller than the ones in well-mixed populations. That is, a structured population enlarges the initial state interval leading to full defection along the DR-edge, thus less effective in utilizing the reward mechanism (Fig. S1**a**, **b**). A structured population becomes more advantageous only when its $x_2^{(DR)}$ are greater than the ones in well-mixed populations. According to Eqs. (S127a) and (S138a), this means

$$\begin{aligned}
&\frac{k(k+1)}{(k^2+k-6)(\gamma-\alpha)}\left(\frac{rc}{k+1} - c\right) + \frac{k^2+k-3}{k^2+k-6} > 1 - \frac{1}{\gamma-\alpha}\left(-\frac{rc}{k+1} + c\right) \\
&\Leftrightarrow \gamma > \gamma_= \equiv \frac{1}{2}\left(-\frac{rc}{k+1} + c\right) + \alpha.
\end{aligned} \tag{S158}$$

Only when $\gamma > \gamma_=$, a structured population has a smaller initial state interval leading to full defection along the DR-edge, thus more effective to utilize the reward mechanism than well-mixed populations. In particular, $\gamma > \gamma^\star$ can even destabilize the full defection equilibrium as shown in Eq. (S155) and transform the system to a rock-paper-scissors-like cyclic dominance, while a well-mixed population cannot realize this (Fig. S1**f**).

Similar principles were also found in pool punishment (Supplementary Note 3.3), whose payoff function is nonlinear. However, through the reward mechanism, we reveal that rock-paper-scissors cycles could emerge even if the payoff function is linear.

These analytical results are in qualitative agreement with the α-γ phase diagrams from previous research[18], as shown in Fig. S2. The phase diagrams under non-marginal selection indicate the existence of a cyclic dominance phase $(D+C+R)_\mathrm{C}$ (Fig. S2**a**, **c**), where strategy D invades C, strategy C invades R, and strategy R invades D. This phase is also predicted under weak selection in the previous analysis. However, the parameter space we show does not include this phase, with the analytical expressions of their boundaries listed in the caption of Fig. S2. Due to the role of a non-marginal selection strength, the effect of reward works in advance and induces the rock-paper-scissors cycling phase as we increase γ. In particular, there is even a $C+R$ phase under non-marginal selection, which does not exist under weak selection. This can be explained by analogizing the reward mechanism to a donation game—R-players donate α to each C- or R-co-player, who receives γ. Under weak selection, evolution cannot favor donors if we apply the pairwise comparison update rule[9]. However, under non-marginal selection, donors may form spatial clusters (which are more remarkable than the ones under weak selection) and thus survive. Again, while there are differences between outcomes derived from non-marginal selection through numerical simulation (Fig. S2**a**, **c**) and those obtained under weak selection with analytical methods (Fig. S2**b**, **d**), both approaches indicate some distinct behavioral patterns in structured populations that are not observed in well-mixed populations.

3.5 The multi-stage public goods game ($n = 4$)

Last, we involve another example, the multi-stage public goods game[15]. In the simplest multi-stage public goods game, we consider two stages. Players can invest (i.e., cooperate) in the first and second stages. Namely, the number of available strategies is $n = 4$:

$1 =$ Cooperation in both stages (CC);

$2 =$ Cooperation only in the first stage (CD);

$3 =$ Cooperation only in the second stage (DC);

$4 =$ Defection in both stages (DD).

The traditional public goods game is equivalent to a one-stage public goods game, where players invest in the only stage and receive the produced public goods. In the extended two-stage public goods game, however, the produced public goods from the first stage (contributed by CC- and CD-players) are not distributed immediately, but reinvested to the second stage. The second stage uses the inherited public goods from the first stage plus the independent investment (contributed by CC- and DC-players) to produce the final public goods. The final public goods are then distributed to all players in the game. We assume that the synergy factor of the first stage is r_1, and the synergy factor of the second stage is r_2.

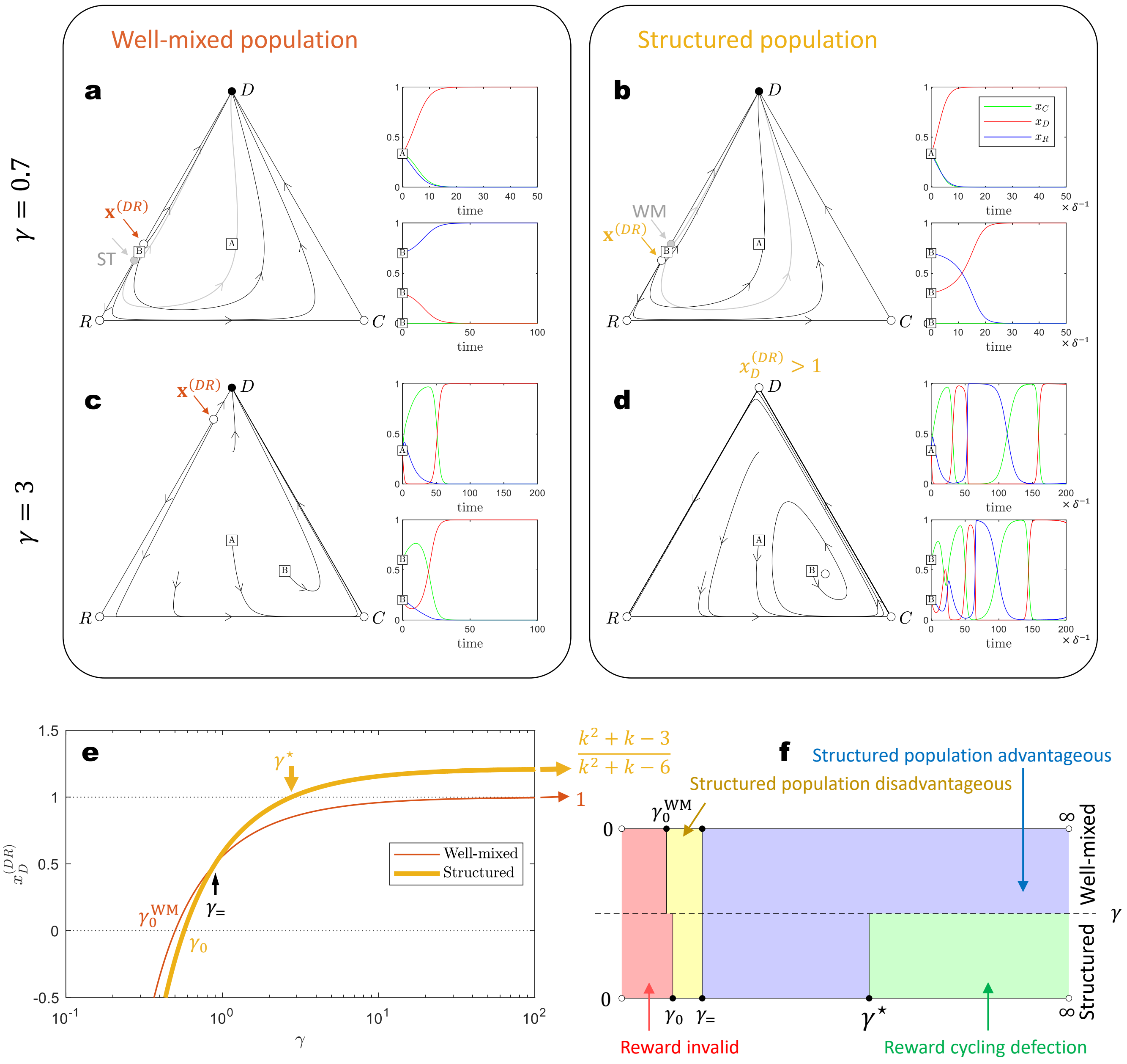

Figure S1. The reward mechanism can resolve the social dilemma of public goods game in structured populations. a and **b**, In the three-strategy system space, the state consistently converges to full D. However, along the DR edge, an unstable equilibrium point, $\mathbf{x}^{(DR)}$, creates a bi-stable space. In the D versus R dynamics, the final state, either D or R, is determined by the initial conditions. Under mild reward ($\gamma = 0.7$), a structured population tends to favor defection, reducing the basin leading to the R outcome. **c** and **d**, Conversely, with strong reward ($\gamma = 3$), structured populations result in the cyclic dominance of the three strategies around the interior equilibrium $\mathbf{x}^{(CDR)}$, thereby preventing the full D state in public goods games. In contrast, the state space in well-mixed populations remains two distinct basins on the DR edge, preventing cyclic dominance. **e**, As the reward strength γ increases, $x_D^{(DR)}$ increases, expanding the initial space leading to the R outcome. In well-mixed populations, $x_D^{(DR)} \to 1$ as $\gamma \to \infty$, and the basin leading to defection cannot be completely eliminated. However, in structured populations, $x_D^{(DR)} \to (k^2+k-3)/(k^2+k-6) > 1$ when $\gamma > \gamma^\star$, invariably resulting in the cyclic dominance of the three strategies. **f**, The diagram of the different effects of reward in well-mixed versus structured populations. Structured populations are advantageous in promoting cooperation under strong reward but are a bit less effective when the reward is mild. **Input parameters**: $r = 3$, $c = 1$, $\alpha = 0.1$, $k = 4$.

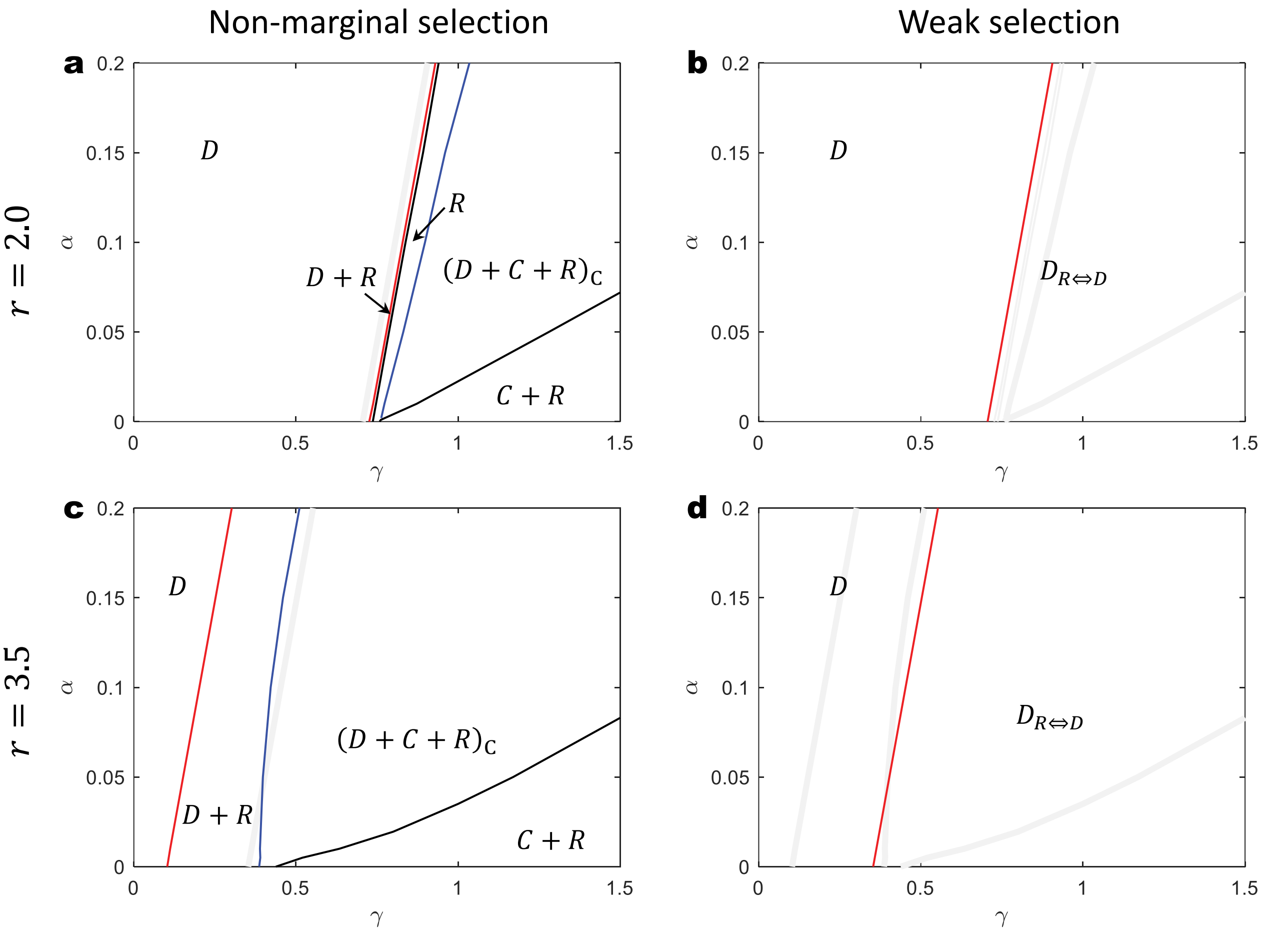

Figure S2. **Phase diagrams of the system behavior with the reward mechanism are qualitatively similar under non-marginal and weak selection strength.** **a** and **c** (the data are in agreement with those published in Figs. 1(a) and 3(a) from ref.[18]), Numerical simulations under non-marginal selection ($\delta = 2$). The phases are defined as follows: D—only D exists; R—only R exists; $(D+C+R)_{\text{C}}$—cyclic dominance among D, C, and R; $D+R$—D and R coexist; $C+R$—C and R coexist. **b** and **d**, The phase diagram is divided by analytical γ_0 and $\gamma^\star$ (not visible in the presented parameter space) under weak selection ($\delta \to 0^+$). Here, γ_0 divides the D and $D_{R\Leftrightarrow D}$ phases, while $\gamma^\star$ separates the $D_{R\Leftrightarrow D}$ and $(D+C+R)_{\text{C}}$ phases. Specifically, in **b**, $\gamma_0 = 12/17+\alpha$ (red), $\gamma^\star = 4+\alpha$ (invisible); in **d**, $\gamma_0 = 6/17+\alpha$, $\gamma^\star = 2+\alpha$ (invisible). The definition of the $D_{R\Leftrightarrow D}$ phase—the system finally evolves to full D if cooperation is initially present, or to the fixation of either R or D in the absence of initial cooperators. **Other parameters:** $c = 1$, $k = 4$.

Therefore, given the co-player configuration $\mathbf{k} = (k_1, k_2, k_3, k_4)$, we have the following payoff calculation in a single multi-stage public goods game.

$$a_{1|\mathbf{k}} = \frac{r_2[r_1(k_1+1+k_2)c+(k_1+1+k_3)c]}{k+1} - 2c = \frac{r_2(r_1+1)c}{k+1}k_1 + \frac{r_2r_1c}{k+1}k_2 + \frac{r_2c}{k+1}k_3 + \frac{r_2(r_1+1)c}{k+1} - 2c, \tag{S159a}$$

$$a_{2|\mathbf{k}} = \frac{r_2[r_1(k_1+k_2+1)c+(k_1+k_3)c]}{k+1} - c = \frac{r_2(r_1+1)c}{k+1}k_1 + \frac{r_2r_1c}{k+1}k_2 + \frac{r_2c}{k+1}k_3 + \frac{r_2r_1c}{k+1} - c, \tag{S159b}$$

$$a_{3|\mathbf{k}} = \frac{r_2[r_1(k_1+k_2)c+(k_1+k_3+1)c]}{k+1} - c = \frac{r_2(r_1+1)c}{k+1}k_1 + \frac{r_2r_1c}{k+1}k_2 + \frac{r_2c}{k+1}k_3 + \frac{r_2c}{k+1} - c, \tag{S159c}$$

$$a_{4|\mathbf{k}} = \frac{r_2[r_1(k_1+k_2)c+(k_1+k_3)c]}{k+1} = \frac{r_2(r_1+1)c}{k+1}k_1 + \frac{r_2r_1c}{k+1}k_2 + \frac{r_2c}{k+1}k_3. \tag{S159d}$$

3.5.1 The structured population

For this multi-stage public goods game model, we find that structured populations are equivalent to well-mixed populations under pairwise comparison and in the weak selection limit. Let us explain why. Observing the payoff structure given by

Eq. (S159), we find it linear. We can again utilize the simplified method for special linear systems given by Supplementary Note 2.4.3 for convenience. Comparing the payoff structure $a_{i|\mathbf{k}}$ with Eqs. (S29)–(S31), we extract matrices **b** and **c**,

$$\mathbf{b}=\begin{pmatrix}\frac{r_2(r_1+1)c}{k+1} & \frac{r_2r_1c}{k+1} & \frac{r_2c}{k+1} & 0\\ \frac{r_2(r_1+1)c}{k+1} & \frac{r_2r_1c}{k+1} & \frac{r_2c}{k+1} & 0\\ \frac{r_2(r_1+1)c}{k+1} & \frac{r_2r_1c}{k+1} & \frac{r_2c}{k+1} & 0\\ \frac{r_2(r_1+1)c}{k+1} & \frac{r_2r_1c}{k+1} & \frac{r_2c}{k+1} & 0\end{pmatrix},\ \mathbf{c}=\begin{pmatrix}\frac{r_2(r_1+1)c}{k+1}-2c\\ \frac{r_2r_1c}{k+1}-c\\ \frac{r_2c}{k+1}-c\\ 0\end{pmatrix}. \tag{S160}$$

The frequencies of strategies 1, 2, 3, 4 are denoted by x_1, x_2, x_3, and x_4. We then calculate

$$3\sum_{j=1}^{4}x_j(b_{ii}-b_{ij}-b_{ji}-b_{jj})=-\frac{6r_2(r_1+1)c}{k+1}x_1-\frac{6r_2r_1c}{k+1}x_2-\frac{6r_2c}{k+1}x_3,\quad \text{for } i=1,2,3,4, \tag{S161a}$$

$$6\sum_{j=1}^{4}\sum_{l=1}^{4}x_jx_lb_{jl}=\frac{6r_2(r_1+1)c}{k+1}x_1+\frac{6r_2r_1c}{k+1}x_2+\frac{6r_2c}{k+1}x_3, \tag{S161b}$$

which leads to

$$b_{ii}-\sum_{j=1}^{4}x_j(b_{ij}+b_{ji}+b_{jj})+2\sum_{j=1}^{4}\sum_{l=1}^{4}x_jx_lb_{jl}=0. \tag{S162}$$

This meets the general condition Eq. (S53) proposed by Supplementary Note 3.1.3 that pairwise comparison equates well-mixed and structured populations under weak selection. The additional effect brought by network structures is zero, and according to Eq. (S35), the replicator equations reduce to $\dot{x}_i \propto x_i(\bar{\pi}_i-\bar{\pi})$, equivalent to the ones in well-mixed populations.

With this in mind, we only put the analysis in the context of structured populations here, using the methods within our framework.

Given the additional terms Eqs. (S161a) and (S161b), we still need calculate the mean payoffs for different strategies in well-mixed populations, which are

$$\bar{\pi}_1=\sum_{k_1+k_2+k_3+k_4=k}\frac{k!}{k_1!k_2!k_3!k_4!}x_1^{k_1}x_2^{k_2}x_3^{k_3}x_4^{k_4}a_{1|\mathbf{k}}=\frac{r_2(r_1+1)c}{k+1}kx_1+\frac{r_2r_1c}{k+1}kx_2+\frac{r_2c}{k+1}kx_3+\frac{r_2(r_1+1)c}{k+1}-c, \tag{S163a}$$

$$\bar{\pi}_2=\sum_{k_1+k_2+k_3+k_4=k}\frac{k!}{k_1!k_2!k_3!k_4!}x_1^{k_1}x_2^{k_2}x_3^{k_3}x_4^{k_4}a_{2|\mathbf{k}}=\frac{r_2(r_1+1)c}{k+1}kx_1+\frac{r_2r_1c}{k+1}kx_2+\frac{r_2c}{k+1}kx_3+\frac{r_2r_1c}{k+1}-c, \tag{S163b}$$

$$\bar{\pi}_3=\sum_{k_1+k_2+k_3+k_4=k}\frac{k!}{k_1!k_2!k_3!k_4!}x_1^{k_1}x_2^{k_2}x_3^{k_3}x_4^{k_4}a_{3|\mathbf{k}}=\frac{r_2(r_1+1)c}{k+1}kx_1+\frac{r_2r_1c}{k+1}kx_2+\frac{r_2c}{k+1}kx_3+\frac{r_2c}{k+1}-c, \tag{S163c}$$

$$\bar{\pi}_4=\sum_{k_1+k_2+k_3+k_4=k}\frac{k!}{k_1!k_2!k_3!k_4!}x_1^{k_1}x_2^{k_2}x_3^{k_3}x_4^{k_4}a_{3|\mathbf{k}}=\frac{r_2(r_1+1)c}{k+1}kx_1+\frac{r_2r_1c}{k+1}kx_2+\frac{r_2c}{k+1}kx_3. \tag{S163d}$$

The mean payoff of the total population is then calculated by

$$\bar{\pi}=x_1\bar{\pi}_1+x_2\bar{\pi}_2+x_3\bar{\pi}_3+x_4\bar{\pi}_4=(k+1)\left(\frac{r_2(r_1+1)c}{k+1}x_1+\frac{r_2r_1c}{k+1}x_2+\frac{r_2c}{k+1}x_3\right)-(2x_1+x_2+x_3)c. \tag{S164}$$

Inserting Eqs. (S161a), (S161b), (S163a)–(S163d), and (S164) into Eq. (S35), we obtain the replicator equations for multi-stage public goods game in structured populations:

$$\dot{x}_1=\frac{\delta(k-2)(k+1)}{2(k-1)}x_1\left[(1-x_1)\left(\frac{r_2(r_1+1)c}{k+1}-2c\right)-x_2\left(\frac{r_2r_1c}{k+1}-c\right)-x_3\left(\frac{r_2c}{k+1}-c\right)\right], \tag{S165a}$$

$$\dot{x}_2=\frac{\delta(k-2)(k+1)}{2(k-1)}x_2\left[-x_1\left(\frac{r_2(r_1+1)c}{k+1}-2c\right)+(1-x_2)\left(\frac{r_2r_1c}{k+1}-c\right)-x_3\left(\frac{r_2c}{k+1}-c\right)\right], \tag{S165b}$$

$$\dot{x}_3 = \frac{\delta(k-2)(k+1)}{2(k-1)} x_3 \left[-x_1 \left(\frac{r_2(r_1+1)c}{k+1} - 2c \right) - x_2 \left(\frac{r_2 r_1 c}{k+1} - c \right) + (1-x_3) \left(\frac{r_2 c}{k+1} - c \right) \right], \tag{S165c}$$

$$\dot{x}_4 = -\dot{x}_1 - \dot{x}_2 - \dot{x}_3. \tag{S165d}$$

We denote the system state $\mathbf{x} = (x_1, x_2, x_3, x_4)$. Solving $\dot{\mathbf{x}} = \mathbf{0}$, we obtain four equilibrium points, denoted by $\mathbf{x}^{(CC)} = (1,0,0,0)$, $\mathbf{x}^{(CD)} = (0,1,0,0)$, $\mathbf{x}^{(DC)} = (0,0,1,0)$, and $\mathbf{x}^{(DD)} = (0,0,0,1)$. To analyze their stability, we study the Jacobian matrix of the system composed by Eqs. (S165a)–(S165c) (cancel $\dot{x}_4 = -\dot{x}_1 - \dot{x}_2 - \dot{x}_3$),

$$J = \begin{pmatrix} \frac{\partial \dot{x}_1}{\partial x_1} & \frac{\partial \dot{x}_1}{\partial x_2} & \frac{\partial \dot{x}_1}{\partial x_3} \\ \frac{\partial \dot{x}_2}{\partial x_1} & \frac{\partial \dot{x}_2}{\partial x_2} & \frac{\partial \dot{x}_2}{\partial x_3} \\ \frac{\partial \dot{x}_3}{\partial x_1} & \frac{\partial \dot{x}_3}{\partial x_2} & \frac{\partial \dot{x}_3}{\partial x_3} \end{pmatrix}$$

$$= \frac{\delta(k-2)(k+1)}{2(k-1)} \begin{pmatrix} J_{11} & -x_1 \left(\frac{r_2 r_1 c}{k+1} - c \right) & -x_1 \left(\frac{r_2 c}{k+1} - c \right) \\ -x_2 \left(\frac{r_2(r_1+1)c}{k+1} - 2c \right) & J_{22} & -x_2 \left(\frac{r_2 c}{k+1} - c \right) \\ -x_3 \left(\frac{r_2(r_1+1)c}{k+1} - 2c \right) & -x_3 \left(\frac{r_2 r_1 c}{k+1} - c \right) & J_{33} \end{pmatrix}, \tag{S166}$$

where

$$J_{11} = (1-2x_1) \left(\frac{r_2(r_1+1)c}{k+1} - 2c \right) - x_2 \left(\frac{r_2 r_1 c}{k+1} - c \right) - x_3 \left(\frac{r_2 c}{k+1} - c \right), \tag{S167a}$$

$$J_{22} = -x_1 \left(\frac{r_2(r_1+1)c}{k+1} - 2c \right) + (1-2x_2) \left(\frac{r_2 r_1 c}{k+1} - c \right) - x_3 \left(\frac{r_2 c}{k+1} - c \right), \tag{S167b}$$

$$J_{33} = -x_1 \left(\frac{r_2(r_1+1)c}{k+1} - 2c \right) - x_2 \left(\frac{r_2 r_1 c}{k+1} - c \right) + (1-2x_3) \left(\frac{r_2 c}{k+1} - c \right). \tag{S167c}$$

Substituting the value of $\mathbf{x}^{(CC)}$ into Eq. (S166), we have

$$J|_{\mathbf{x}=\mathbf{x}^{(CC)}} = \frac{\delta(k-2)(k+1)}{2(k-1)} \begin{pmatrix} -\frac{r_2(r_1+1)c}{k+1} + 2c & -\frac{r_2 r_1 c}{k+1} + c & -\frac{r_2 c}{k+1} + c \\ 0 & -\frac{r_2 c}{k+1} + c & 0 \\ 0 & 0 & -\frac{r_2 r_1 c}{k+1} + c \end{pmatrix}. \tag{S168}$$

The conditions ensuring $J|_{\mathbf{x}=\mathbf{x}^{(CC)}}$ negative-definite are $r_1 r_2 > 2(k+1)$, $r_2 > k+1$, and $r_1 r_2 > k+1$. The last one covers the first one. Therefore, the equilibrium point $\mathbf{x}^{(CC)}$ is stable if and only if $r_1 r_2 > k+1$, $r_2 > k+1$.

Substituting the value of $\mathbf{x}^{(CD)}$ into Eq. (S166), we have

$$J|_{\mathbf{x}=\mathbf{x}^{(CD)}} = \frac{\delta(k-2)(k+1)}{2(k-1)} \begin{pmatrix} \frac{r_2 c}{k+1} - c & 0 & 0 \\ -\frac{r_2(r_1+1)c}{k+1} + 2c & -\frac{r_2 r_1 c}{k+1} + c & -\frac{r_2 c}{k+1} + c \\ 0 & 0 & -\frac{r_2(r_1-1)c}{k+1} \end{pmatrix}. \tag{S169}$$

The conditions ensuring $J|_{\mathbf{x}=\mathbf{x}^{(CD)}}$ negative-definite are $r_2 < k+1$, $r_1 r_2 > k+1$, and $r_1 > 1$. If the first two conditions hold, then the last one is naturally valid. Therefore, $\mathbf{x}^{(CD)}$ is stable if and only if $r_1 r_2 > k+1$, $r_2 < k+1$.

Substituting the value of $\mathbf{x}^{(DC)}$ into Eq. (S166), we have

$$J|_{\mathbf{x}=\mathbf{x}^{(DC)}} = \frac{\delta(k-2)(k+1)}{2(k-1)} \begin{pmatrix} \frac{r_2 r_1 c}{k+1} - c & 0 & 0 \\ 0 & \frac{r_2(r_1-1)c}{k+1} - c & 0 \\ -\frac{r_2(r_1+1)c}{k+1} + 2c & -\frac{r_2 r_1 c}{k+1} + c & -\frac{r_2 c}{k+1} + c \end{pmatrix}. \tag{S170}$$

The conditions ensuring $J|_{\mathbf{x}=\mathbf{x}^{(DC)}}$ negative-definite are $r_1 r_2 < k+1$, $r_1 < 1$, and $r_2 > k+1$. If the first and third conditions hold, then the second one is naturally valid. Therefore, $\mathbf{x}^{(DC)}$ is stable if and only if $r_1 r_2 < k+1$, $r_2 > k+1$.

Substituting the value of $\mathbf{x}^{(DD)}$ into Eq. (S166), we have

$$J|_{\mathbf{x}=\mathbf{x}^{(DD)}} = \frac{\delta(k-2)(k+1)}{2(k-1)} \begin{pmatrix} \frac{r_2(r_1+1)c}{k+1} - 2c & 0 & 0 \\ 0 & \frac{r_2 r_1 c}{k+1} - c & 0 \\ 0 & 0 & \frac{r_2 c}{k+1} - c \end{pmatrix}. \tag{S171}$$

The conditions ensuring $J|_{\mathbf{x}=\mathbf{x}^{(DD)}}$ negative-definite are $r_2(r_1+1) < 2(k+1)$, $r_1 r_2 < k+1$, and $r_2 < k+1$. If the second and third conditions hold, then the first one is naturally valid. Therefore, $\mathbf{x}^{(DD)}$ is stable if and only if $r_1 r_2 < k+1$, $r_2 < k+1$.

3.5.2 Discussion

To compare our analytical results under weak selection and the numerical ones from previous work under non-marginal selection[15], we refer to their phase diagrams on the r_1-r_2 plane (Fig. S3). Under a non-marginal selection strength (Fig. S3**a**), there are three pure phases as r_1 and r_2 increase, from DD, through CD, to CC. Between these pure phases, there are mixed phases, such as the $DD+CD$ phase (between DD and CC) and the $CD+CC$ phase (between CD and CC). However, in the weak selection limit, these mixed phases do not exist (Fig. S3**b**). On the presented phase diagrams, there are only three pure phases. This is similar to the phenomenon in the traditional public goods game, where only C and D phases exist under weak selection (Supplementary Note 3.1.2) and the mixed phase $C+D$ only exists under non-marginal selection[19]. In the extended multi-stage public goods game, the predicted phase boundaries under weak selection (Fig. S3**b**) qualitatively match the ones under non-marginal selection (Fig. S3**a**), illustrating the utility of our analytical framework.

Our analytical framework also predicts a DC phase, which exists when $r_1 r_2 < k+1$ and $r_2 > k+1$ (see Eq. (S170)). This region is outside the upper left corner of Fig. S3**b** and is invisible in the parameter space we present. In this region, $r_1 < 1$, which is not meaningful in the sense of public goods games. Therefore, neither the previous numerical work nor our analytical framework shows the region where the DC phase exists.

Supplementary Note 4: Death-birth

Here, we supplement the corresponding results under the death-birth rule.

In a unit time, a random focal individual A is selected to die, and A's neighbors compete for the vacant position proportional to their fitness. The neighbor B reproduces its strategy to the position of A with the probability as follows:

$$W = \frac{F_B}{\sum_k F_X}, \tag{S172}$$

where $\sum_k F_X$ represents the total fitness of A's neighbors. If individual B has a higher fitness among A's neighbors, then B has a higher probability to reproduce its strategy to the position of A. It should be noted that the focal individual's fitness is completely ignored under death-birth.

Below, we analyze the dynamics of the death-birth rule rigorously.

4.1 The increase of i-players

The increase of i-players happens when a focal j-player ($j \neq i$) is selected to update its strategy and an i-player takes the position. Given the focal j-player's neighbor configuration $\mathbf{k}$, the probability that an i-player takes the j-player's position is

$$\mathscr{P}(j \leftarrow i) = \frac{k_i F_{i|j}^{\mathbf{k}}}{\sum_{i'=1}^{n} k_{i'} F_{i'|j}^{\mathbf{k}}} = \frac{k_i}{k} + \frac{k_i}{k}\left(\pi_{i|j}^{\mathbf{k}} - \sum_{i'=1}^{n} \frac{k_{i'}}{k}\pi_{i'|j}^{\mathbf{k}}\right)\delta + \mathscr{O}(\delta^2). \tag{S173}$$

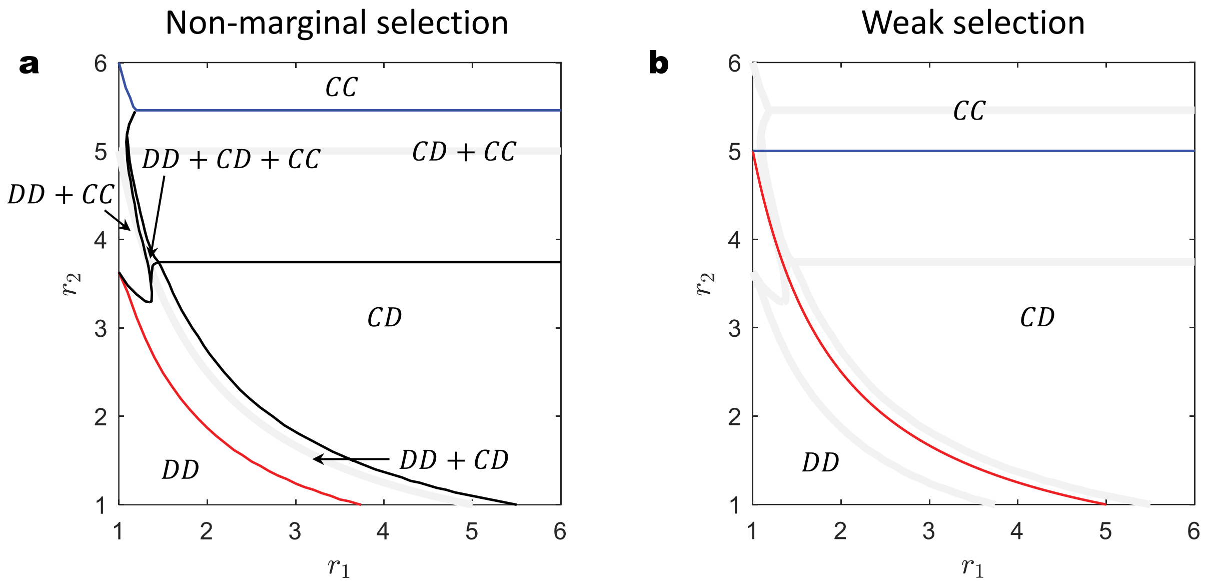

Figure S3. Phase diagrams of the system behavior in multi-stage public goods games are qualitatively similar under non-marginal and weak selection strength. a (the data are in agreement with those published in Fig. 2 from ref.[15]), Numerical simulations under non-marginal selection ($\delta = 2$). The phases are defined directly by their name. For example, CC represents a phase where only CC exists, and $CD+CC$ represents a phase where CC and CD coexist. **b**, The phase diagram is divided by analytical $r_1 r_2 = k+1$ (red) and $r_2 = k+1$ (blue) under weak selection ($\delta \to 0^+$). Here, $r_1 r_2 = k+1$ divides the DD and CD phases, while $r_2 = k+1$ separates the CD and CC phases. **Other parameters:** $c = 1$, $k = 4$.

Then, we apply it to all possibilities for $j \neq i$ and neighbor configurations $\mathbf{k}$, obtaining the probability that the number of i-players increases by 1 during a unit time step,

$$\mathscr{P}\left(\Delta x_i = \frac{1}{N}\right) = \sum_{j=1, j\neq i}^{n} x_j \sum_{\sum_{i'=1}^{n} k_{i'} = k} \frac{k!}{\prod_{i'=1}^{n} k_{i'}!} \left(\prod_{i'=1}^{n} {q_{i'|j}}^{k_{i'}}\right) \mathscr{P}(j \leftarrow i)$$
$$= \sum_{j=1, j\neq i}^{n} x_j q_{i|j} + \sum_{j=1, j\neq i}^{n} x_j \sum_{\sum_{i'=1}^{n} k_{i'} = k} \frac{k!}{\prod_{i'=1}^{n} k_{i'}!} \left(\prod_{i'=1}^{n} {q_{i'|j}}^{k_{i'}}\right) \frac{k_i}{k} \left(\pi_{i|j}^{\mathbf{k}} - \sum_{i'=1}^{n} \frac{k_{i'}}{k} \pi_{i'|j}^{\mathbf{k}}\right) \delta + \mathscr{O}(\delta^2). \quad \text{(S174)}$$

4.2 The decrease of i-players

The decrease of i-players happens when a focal i-player is selected to update its strategy and the player who takes the position is not an i-player. Unlike pairwise comparison, where the focal individual has a probability of keeping its own strategy, $\sum_{j=1}^{n} \mathscr{P}(i \leftarrow j) \neq 1$, here, under death-birth, the focal individual must adopt the strategy of a neighbor, $\sum_{j=1}^{n} \mathscr{P}(i \leftarrow j) = 1$. Therefore, given the focal i-player's neighbor configuration $\mathbf{k}$, the probability that the player who takes the position is not an i-player can be written as

$$1 - \mathscr{P}(i \leftarrow i) = 1 - \frac{k_i F_{i|i}^{\mathbf{k}}}{\sum_{i'=1}^{n} k_{i'} F_{i'|i}^{\mathbf{k}}} = \frac{k - k_i}{k} - \frac{k_i}{k}\left(\pi_{i|i}^{\mathbf{k}} - \sum_{i'=1}^{n} \frac{k_{i'}}{k} \pi_{i'|i}^{\mathbf{k}}\right) \delta + \mathscr{O}(\delta^2). \quad \text{(S175)}$$

Applying it to all possibilities for the neighbor configuration $\mathbf{k}$ after selecting a focal i-player with probability x_i, we obtain the probability that the number of i-players decreases by 1 during a unit time step,

$$\mathscr{P}\left(\Delta x_i = -\frac{1}{N}\right) = x_i \sum_{\sum_{i'=1}^{n} k_{i'} = k} \frac{k!}{\prod_{i'=1}^{n} k_{i'}!} \left(\prod_{i'=1}^{n} {q_{i'|i}}^{k_{i'}}\right) [1 - \mathscr{P}(i \leftarrow i)]$$
$$= x_i(1 - q_{i|i}) - x_i \sum_{\sum_{i'=1}^{n} k_{i'} = k} \frac{k!}{\prod_{i'=1}^{n} k_{i'}!} \left(\prod_{i'=1}^{n} {q_{i'|i}}^{k_{i'}}\right) \frac{k_i}{k} \left(\pi_{i|i}^{\mathbf{k}} - \sum_{i'=1}^{n} \frac{k_{i'}}{k} \pi_{i'|i}^{\mathbf{k}}\right) \delta + \mathscr{O}(\delta^2). \quad \text{(S176)}$$

4.3 The replicator equation

The instant change in the proportion x_i of i-players consists of the increase and decrease of i-players. Applying Eqs. (S174) and (S176), and considering that a full Monte Carlo step contains N elementary steps, we have

$$
\begin{aligned}
\dot{x}_i &= N \times \left\{ \frac{1}{N} \mathscr{P}\left(\Delta x_i = \frac{1}{N}\right) + \left(-\frac{1}{N}\right) \mathscr{P}\left(\Delta x_i = -\frac{1}{N}\right) \right\} \\
&= \sum_{j=1}^{n} x_j \sum_{\sum_{i'=1}^{n} k_{i'}=k} \frac{k!}{\prod_{i'=1}^{n} k_{i'}!} \left(\prod_{i'=1}^{n} q_{i'|j}{}^{k_{i'}} \right) \frac{k_i}{k} \left(\pi_{i|j}^{\mathbf{k}} - \sum_{i'=1}^{n} \frac{k_{i'}}{k} \pi_{i'|j}^{\mathbf{k}} \right) \delta + \mathscr{O}(\delta^2).
\end{aligned} \tag{S177}
$$

In Eq. (S177), the δ^0 term has been eliminated: applying Eq. (S1), we know $x_j q_{i|j} = p_{ij} = x_i q_{j|i}$, such that the δ^0 term in Eq. (S174) can be expressed as $\sum_{j=1,j\neq i}^{n} x_j q_{i|j} = \sum_{j=1,j\neq i}^{n} x_i q_{j|i}$; similarly, we know $1 - q_{i|i} = \sum_{j=1,j\neq i}^{n} q_{j|i}$ by Eqs. (S1), which rewrites the δ^0 term in Eq. (S176) as $x_i(1-q_{i|i}) = x_i \sum_{j=1,j\neq i}^{n} q_{j|i}$, equal to the one in Eq. (S174).

The δ^0 term being eliminated, the instant change in x_i happens on the order of δ^1. Meanwhile, the instant change in $q_{i|j}$ for $i, j = 1, 2, \ldots, n$ happens on the order of δ^0 since the δ^0 is non-zero (see Supplementary Note 5.2). That is, the change in $q_{i|j}$ is much faster than x_i, so that x_i changes on the basis of $q_{i|j}$ achieving equilibrium. According to Supplementary Note 5.2, we have the following solution when $q_{i|j}$ achieves stability.

$$
q_{i|j} = \begin{cases} \dfrac{k-2}{k-1} x_i, & j \neq i, \\[2ex] \dfrac{k-2}{k-1} x_i + \dfrac{1}{k-1}, & j = i. \end{cases} \tag{S178}
$$

Eq. (S178) is independently derived under death-birth but appear the same as the ones under pairwise comparison.

The primitive replicator equation of x_i under death-birth is Eq. (S177), where $\mathscr{O}(\delta^2) = 0$, $\pi_j^{\mathbf{k}}$ and $\pi_i^{\mathbf{k}}$ are given by Eq. (S3), $\pi_{i|j}^{\mathbf{k}}$ and $\pi_{j|i}^{\mathbf{k}}$ are given by Eq. (S5), and $q_{i'|j}$ is given by Eq. (S178). The degrees of freedom of the replicator dynamics system are $n-1$, represented by independent variables x_i ($i = 1, 2, \ldots, n$, cancel one of them by $\sum_i^n x_i = 1$).

4.4 Simplification and discussion

Next, we utilize the useful Theorem 1 to simplify the replicator equation under death-birth and discuss it.

4.4.1 Decomposition to accumulated payoff

First, we decompose the general replicator equations by keeping expected accumulated payoffs. Using Theorem 1, we can calculate Eq. (S177) as follows.

$$
\begin{aligned}
\dot{x}_i &= \delta \sum_{j=1}^{n} x_j \sum_{\sum_{i'=1}^{n} k_{i'}=k} \frac{k!}{\prod_{i'=1}^{n} k_{i'}!} \left(\prod_{i'=1}^{n} q_{i'|j}{}^{k_{i'}} \right) \frac{k_i}{k} \left(\pi_{i|j}^{\mathbf{k}} - \sum_{i'=1}^{n} \frac{k_{i'}}{k} \pi_{i'|j}^{\mathbf{k}} \right) \\
&= \delta \sum_{j=1}^{n} x_j q_{i|j} \sum_{\sum_{i'=1}^{n} k_{i'}=k-1} \frac{(k-1)!}{\prod_{i'=1}^{n} k_{i'}!} \left(\prod_{i'=1}^{n} q_{i'|j}{}^{k_{i'}} \right) \pi_{i|j}^{\mathbf{k}+i} \\
&\quad - \delta \sum_{j=1}^{n} x_j q_{i|j} \sum_{\sum_{i'=1}^{n} k_{i'}=k-1} \frac{(k-1)!}{\prod_{i'=1}^{n} k_{i'}!} \left(\prod_{i'=1}^{n} q_{i'|j}{}^{k_{i'}} \right) \left(\frac{k_i+1}{k} \pi_{i|j}^{\mathbf{k}+i} + \sum_{i'=1, i'\neq i}^{n} \frac{k_{i'}}{k} \pi_{i'|j}^{\mathbf{k}+i} \right) \\
&= \delta \sum_{j=1}^{n} x_j q_{i|j} \sum_{\sum_{i'=1}^{n} k_{i'}=k-1} \frac{(k-1)!}{\prod_{i'=1}^{n} k_{i'}!} \left(\prod_{i'=1}^{n} q_{i'|j}{}^{k_{i'}} \right) \pi_{i|j}^{\mathbf{k}+i} \\
&\quad - \delta \sum_{j=1}^{n} x_j q_{i|j} \sum_{\sum_{i'=1}^{n} k_{i'}=k-1} \frac{(k-1)!}{\prod_{i'=1}^{n} k_{i'}!} \left(\prod_{i'=1}^{n} q_{i'|j}{}^{k_{i'}} \right) \left(\frac{1}{k} \pi_{i|j}^{\mathbf{k}+i} + \sum_{i'=1}^{n} \frac{k_{i'}}{k} \pi_{i'|j}^{\mathbf{k}+i} \right) \\
&= \frac{\delta(k-1)}{k} x_i \sum_{j=1}^{n} q_{j|i} \sum_{\sum_{i'=1}^{n} k_{i'}=k-1} \frac{(k-1)!}{\prod_{i'=1}^{n} k_{i'}!} \left(\prod_{i'=1}^{n} q_{i'|j}{}^{k_{i'}} \right) \pi_{i|j}^{\mathbf{k}+i} \\
&\quad - \frac{\delta(k-1)}{k} x_i \sum_{j=1}^{n} q_{j|i} \sum_{\sum_{i'=1}^{n} k_{i'}=k-2} \frac{(k-2)!}{\prod_{i'=1}^{n} k_{i'}!} \left(\prod_{i'=1}^{n} q_{i'|j}{}^{k_{i'}} \right) \sum_{i'=1}^{n} q_{i'|j} \pi_{i'|j}^{\mathbf{k}+i,+i'}.
\end{aligned} \tag{S179}
$$

Using the aforementioned notations for $\langle \pi_{i|j}^{\mathbf{k}_{+i}} \rangle$ in Eq. (S16), as well as its extension for $\langle \pi_{i'|j}^{\mathbf{k}_{+i,+i'}} \rangle$,

$$\langle \pi_{i'|j}^{\mathbf{k}_{+i,+i'}} \rangle = \sum_{\sum_{i'=1}^{n} k_{i'}=k-2} \frac{(k-2)!}{\prod_{i'=1}^{n} k_{i'}!} \left(\prod_{i'=1}^{n} q_{i'|j}{}^{k_{i'}} \right) \pi_{i'|j}^{\mathbf{k}_{+i,+i'}}, \tag{S180}$$

we can write Eq. (S179) as

$$\begin{aligned} \dot{x}_i &= \frac{\delta(k-1)}{k} x_i \sum_{j=1}^{n} q_{j|i} \left(\langle \pi_{i|j}^{\mathbf{k}_{+i}} \rangle - \sum_{i'=1}^{n} q_{i'|j} \langle \pi_{i'|j}^{\mathbf{k}_{+i,+i'}} \rangle \right) \\ &= \frac{\delta(k-1)}{k} x_i \left(\langle \pi_i^{\mathbf{k}} \rangle - \sum_{j=1}^{n} \sum_{i'=1}^{n} q_{i'|j} q_{j|i} \langle \pi_{i'|j}^{\mathbf{k}_{+i,+i'}} \rangle \right). \end{aligned} \tag{S181}$$

In Eq. (S181), we have used the relation $\sum_{j=1}^{n} q_{j|i} \langle \pi_{i|j}^{\mathbf{k}_{+i}} \rangle = \langle \pi_i^{\mathbf{k}} \rangle$ as suggested by Theorem 3.

Again, the replicator equation expressed by expected accumulated payoffs given by Eq. (S181) bears an intuitive understanding if we use the following concepts:

- $\pi_i^{(0)} = \langle \pi_i^{\mathbf{k}} \rangle$, the expected accumulated payoff of the i-player itself (zero-step away on the graph).
- $\pi_i^{(2)} = \sum_{j=1}^{n} \sum_{i'=1}^{n} q_{i'|j} q_{j|i} \langle \pi_{i'|j}^{\mathbf{k}_{+i,+i'}} \rangle$, the expected accumulated payoff of the i-player's second-order neighbors (two-step away on the graph). Intuitively, $q_{i'|j} q_{j|i}$ is used to find two-step away i'-players away from the i-player. The first step walks to a j-player, whose neighbor configuration is $\mathbf{k}_{+i,+i'}$. The second step walks to an i'-player.

Using these concepts, we know that $\dot{x}_i \propto x_i(\pi_i^{(0)} - \pi_i^{(2)})$. Under death-birth, the reproduction rate of i-players depends on how much their expected accumulated payoff higher than the one of neighbors' neighbors. The essence of replicator dynamics $\dot{x}_i$ is the competition between oneself and its second-order neighbors. This is also consistent with the result obtained by the identity-by-descent idea[9,10], and here we further generalize it to n-strategy multiplayer systems.

4.4.2 Decomposition to single-game payoff

Next, we further divide the replicator equation into expected single-game payoffs, stressing the convenience for actual calculation.

Similarly, we use the notation of $\langle a_{i|\mathbf{k}} \rangle_j$ defined in Eq. (S21) to represent the payoff in a single game. We know the expression of $\langle \pi_i^{\mathbf{k}} \rangle$ from Eq. (S23), which is derived from the payoff calculation stage, independent of strategy update rules. Furthermore, we expand $\langle \pi_{i'|j}^{\mathbf{k}_{+i,+i'}} \rangle$ by Eq. (S5) and obtain

$$\begin{aligned} \langle \pi_{i'|j}^{\mathbf{k}_{+i,+i'}} \rangle &= \sum_{\sum_{i'=1}^{n} k_{i'}=k-2} \frac{(k-2)!}{\prod_{i'=1}^{n} k_{i'}!} \left(\prod_{i'=1}^{n} q_{i'|j}{}^{k_{i'}} \right) \left\{ a_{i'|\mathbf{k}_{+i,+j}} + \sum_{\sum_{l=1}^{n} k'_l=k-1} \frac{(k-1)!}{\prod_{l=1}^{n} k'_l!} \left(\prod_{l=1}^{n} q_{l|i'}{}^{k'_l} \right) \left[a_{i'|\mathbf{k}'_{+j}} \right.\right. \\ &\qquad \left.\left. + \sum_{l=1}^{n} k'_l \sum_{\sum_{\ell=1}^{n} k''_\ell=k-1} \frac{(k-1)!}{\prod_{\ell=1}^{n} k''_\ell!} \left(\prod_{\ell=1}^{n} q_{\ell|l}{}^{k''_\ell} \right) a_{i'|\mathbf{k}''_{+l}} \right] \right\} \\ &= \sum_{\sum_{i'=1}^{n} k_{i'}=k-2} \frac{(k-2)!}{\prod_{i'=1}^{n} k_{i'}!} \left(\prod_{i'=1}^{n} q_{i'|j}{}^{k_{i'}} \right) a_{i'|\mathbf{k}_{+i,+j}} + \sum_{\sum_{l=1}^{n} k'_l=k-1} \frac{(k-1)!}{\prod_{l=1}^{n} k'_l!} \left(\prod_{l=1}^{n} q_{l|i'}{}^{k'_l} \right) a_{i'|\mathbf{k}'_{+j}} \\ &\qquad + (k-1) \sum_{l=1}^{n} q_{l|i'} \sum_{\sum_{\ell=1}^{n} k''_\ell=k-1} \frac{(k-1)!}{\prod_{\ell=1}^{n} k''_\ell!} \left(\prod_{\ell=1}^{n} q_{\ell|l}{}^{k''_\ell} \right) a_{i'|\mathbf{k}''_{+l}} \\ &= \langle a_{i'|\mathbf{k}_{+i,+j}} \rangle_j + \langle a_{i'|\mathbf{k}_{+j}} \rangle_{i'} + (k-1) \sum_{l=1}^{n} q_{l|i'} \langle a_{i'|\mathbf{k}_{+l}} \rangle_l. \end{aligned} \tag{S182}$$

Applying Eqs. (S23) and (S182) to Eq. (S181), we have

$$\dot{x}_i = \frac{\delta(k-1)}{k} x_i \left[\left(\langle a_{i|\mathbf{k}} \rangle_i + k \sum_{j=1}^{n} q_{j|i} \langle a_{i|\mathbf{k}_{+j}} \rangle_j \right) - \sum_{j=1}^{n} \sum_{i'=1}^{n} q_{i'|j} q_{j|i} \left(\langle a_{i'|\mathbf{k}_{+j}} \rangle_{i'} + \langle a_{i'|\mathbf{k}_{+i,+j}} \rangle_j + (k-1) \sum_{l=1}^{n} q_{l|i'} \langle a_{i'|\mathbf{k}_{+l}} \rangle_l \right) \right]. \tag{S183}$$

To decrease the number of elements, we transform $\langle a_{i|\mathbf{k}}\rangle_i$ into $\sum_{j=1}^n q_{j|i}\langle a_{i|\mathbf{k}_{+j}}\rangle_i$ according to Theorem 4 and rewrite Eq. (S183) as

$$\dot{x}_i = \frac{\delta(k-1)}{k} x_i \sum_{j=1}^n q_{j|i} \left(\langle a_{i|\mathbf{k}_{+j}}\rangle_i + k\langle a_{i|\mathbf{k}_{+j}}\rangle_j - \sum_{i'=1}^n q_{i'|j} \left(\langle a_{i'|\mathbf{k}_{+j}}\rangle_{i'} + \langle a_{i'|\mathbf{k}_{+i,+j}}\rangle_j + (k-1)\sum_{l=1}^n q_{l|i'}\langle a_{i'|\mathbf{k}_{+l}}\rangle_l \right)\right). \quad \text{(S184)}$$

We do not bother giving the general expression of Eq. (S184) with all $q_{j|i}$ quantities transformed into x_j, since the results would be complicated and unruly. Please note that the calculation inside each $\langle\cdot\rangle$ also contains $q_{j|i}$ quantities and we always need to transform them manually in applications.

According to Eq. (S184), we can attribute everything about $\langle\cdot\rangle$ into three types, the '$\langle a_{i|\mathbf{k}_{+j}}\rangle_i$ type', the '$\langle a_{i|\mathbf{k}_{+j}}\rangle_j$ type', and the '$\langle a_{i'|\mathbf{k}_{+i,+j}}\rangle_j$ type'. The $\langle a_{i|\mathbf{k}_{+j}}\rangle_i$ and $\langle a_{i|\mathbf{k}_{+j}}\rangle_j$ types have been listed as matrices $\left[\langle a_{i|\mathbf{k}_{+j}}\rangle_i\right]_{ij}$ and $\left[\langle a_{i|\mathbf{k}_{+j}}\rangle_j\right]_{ij}$ in Eqs. (S27) and (S28), respectively. The number of elements to calculate manually in the $\langle a_{i|\mathbf{k}_{+j}}\rangle_i$ and $\langle a_{i|\mathbf{k}_{+j}}\rangle_j$ types is $(2n-1)n$ as we have discussed.

The $\langle a_{i'|\mathbf{k}_{+i,+j}}\rangle_j$ type, however, contains three dimensions. It can be expressed by totaling i' matrices, each with dimensions i and j:

- The $\langle a_{i'|\mathbf{k}_{+i,+j}}\rangle_j$ type:

$$\left[\langle a_{i'|\mathbf{k}_{+i,+j}}\rangle_j\right]_{ij} = \begin{pmatrix} \langle a_{i'|\mathbf{k}_{+1,+1}}\rangle_1 & \langle a_{i'|\mathbf{k}_{+1,+2}}\rangle_2 & \cdots & \langle a_{i'|\mathbf{k}_{+1,+n}}\rangle_n \\ \langle a_{i'|\mathbf{k}_{+2,+1}}\rangle_1 & \langle a_{i'|\mathbf{k}_{+2,+2}}\rangle_2 & \cdots & \langle a_{i'|\mathbf{k}_{+2,+n}}\rangle_n \\ \vdots & \vdots & \ddots & \vdots \\ \langle a_{i'|\mathbf{k}_{+n,+1}}\rangle_1 & \langle a_{i'|\mathbf{k}_{+n,+2}}\rangle_2 & \cdots & \langle a_{i'|\mathbf{k}_{+n,+n}}\rangle_n \end{pmatrix}, \quad \text{(S185)}$$

  for $i' = 1,2,\ldots,n$.

There are n^3 elements in all $\left[\langle a_{i'|\mathbf{k}_{+i,+j}}\rangle_j\right]_{ij}$ matrices. Therefore, the total computation involves $n^3+(2n-1)n = (n^2+2n-1)n$ elements. The computational complexity is $\mathrm{O}(n^3)$, which is feasible within polynomial time.

4.4.3 Special linear system

We can also further simplify the calculation when faced with special payoff structures. Given a co-player configuration $\mathbf{k}$, a linear payoff structure can be determined by the coefficient matrix $\mathbf{b}$ and the constant vector $\mathbf{c}$, that is, $a_{i|\mathbf{k}} = \sum_{l=1}^n b_{il}k_l + c_i$, as previously shown in Eqs. (S29)–(S31).

The expressions of $\langle a_{i|\mathbf{k}_{+j}}\rangle_i$ and $\langle a_{i|\mathbf{k}_{+j}}\rangle_j$ as functions of $\mathbf{b}$ and $\mathbf{c}$ has been obtained in Eqs. (S34a) and (S34b), respectively. We further calculate the elements of the $\langle a_{i'|\mathbf{k}_{+i,+j}}\rangle_j$ type,

$$\langle a_{i'|\mathbf{k}_{+i,+j}}\rangle_j = (k-2)\sum_{l=1}^n b_{i'l}q_{l|j} + b_{i'i} + b_{i'j} + c_{i'} = \frac{(k-2)^2}{k-1}\sum_{l=1}^n b_{i'l}x_l + \frac{2k-3}{k-1}b_{i'j} + b_{i'i} + c_{i'}. \quad \text{(S186)}$$

We substitute Eqs. (S34a), (S34b) and (S186) into Eq. (S184). Through a long but feasible calculation (transforming all $q_{j|i}$ quantities to x_j quantities and organizing the result), we obtain

$$\dot{x}_i = \frac{\delta(k-2)}{k(k-1)^2} x_i \left\{ \frac{(k^2-2)^2}{k}(\bar{\pi}_i - \bar{\pi}) + \frac{3k^2-4}{k}\left((kb_{ii}+c_i) - \sum_{j=1}^n x_j(kb_{jj}+c_j) \right) \right.$$
$$\left. -(k^2+2k-4)\sum_{j=1}^n x_j \left(b_{ji} - \sum_{l=1}^n x_l b_{jl} \right) \right\}, \quad \text{(S187)}$$

where $\bar{\pi}_i = k\sum_{l=1}^n x_l b_{il} + c_i$ is the mean payoff of i-players in a well-mixed population, and $\bar{\pi} = \sum_{i=1}^n x_i\bar{\pi}_i$ is the mean payoff of all individuals in a well-mixed population.

We know that the replicator equation in a well-mixed population is $\dot{x}_i = x_i(\bar{\pi}_i - \bar{\pi})$. In this way, Eq. (S187) clearly showed the additional terms brought by death-birth in a structured population compared to the well-mixed population.

4.5 Comparison with two-strategy dynamics

Let us discuss on how our replicator equations under death-birth for n-strategy systems reduce to the 2-strategy system. Similarly, the configuration $\mathbf{k}=(k_1,k_2)=(k_1,k-k_1)$ can be represented by only k_1, where $k_1=0,1,\ldots,k$.

The n-strategy system simplified to expected accumulated payoffs shown in Eq. (S181) is for comparison with the identity-by-decent idea by Allen and Nowak[9], that the essence of death-birth is the competition from the individuals from two-step away. However, in this section, we want to compare with the 2-strategy system for general multiplayer games in structured populations, which was studied by Li *et al.*[3]. In this way, we need to reorganize the results in Eq. (S181) first.

According to Theorem 5, we have $\langle \pi_i^{\mathbf{k}} \rangle = \sum_{j=1}^{n}\sum_{i'=1}^{n} q_{i'|j}q_{j|i}\langle \pi_{i|j}^{\mathbf{k}_{+i,+i'}}\rangle$. Substituting this into Eq. (S181), we have

$$\dot{x}_i=\frac{\delta(k-1)}{k}x_i\sum_{j=1}^{n}\sum_{i'=1}^{n}q_{i'|j}q_{j|i}\left(\langle\pi_{i|j}^{\mathbf{k}_{+i,+i'}}\rangle-\langle\pi_{i'|j}^{\mathbf{k}_{+i,+i'}}\rangle\right). \tag{S188}$$

Further replacing all $q_{j|i}$ quantities with x_j quantities by Eq. (S178) and organizing the results, we have

$$\dot{x}_i=\frac{\delta(k-2)}{k(k-1)}x_i\sum_{j=1}^{n}x_j\left[\left(\langle\pi_{i|j}^{\mathbf{k}_{+i,+j}}\rangle-\langle\pi_{j|j}^{\mathbf{k}_{+i,+j}}\rangle\right)+\left(\langle\pi_{i|i}^{\mathbf{k}_{+i,+j}}\rangle-\langle\pi_{j|i}^{\mathbf{k}_{+i,+j}}\rangle\right)+(k-2)\sum_{i'=1}^{n}x_{i'}\left(\langle\pi_{i|j}^{\mathbf{k}_{+i,+i'}}\rangle-\langle\pi_{i'|j}^{\mathbf{k}_{+i,+i'}}\rangle\right)\right], \tag{S189}$$

which can be reduced to 2-strategy system depicted by $\dot{x}_1$ with the consideration of $x_2=1-x_1$,

$$\begin{aligned}\dot{x}_1=&\frac{\delta(k-2)}{k(k-1)}x_1(1-x_1)\Big\{k\left(\langle\pi_{1|2}^{\mathbf{k}_{+1,+2}}\rangle-\langle\pi_{2|2}^{\mathbf{k}_{+1,+2}}\rangle\right)\\&+[(k-2)x_1+1]\left[\left(\langle\pi_{1|1}^{\mathbf{k}_{+1,+2}}\rangle-\langle\pi_{2|1}^{\mathbf{k}_{+1,+2}}\rangle\right)-\left(\langle\pi_{1|2}^{\mathbf{k}_{+1,+2}}\rangle-\langle\pi_{2|2}^{\mathbf{k}_{+1,+2}}\rangle\right)\right]\Big\}.\end{aligned} \tag{S190}$$

Eq. (S190), which describes general 2-strategy multiplayer games in structured populations under the death-birth rule, has the same form as the one given by Li *et al.*[3]. As a supplement to their work, here we stress a detail as indicated in Eq. (S190): the calculation should be made in the remaining $k-2$ individuals given the existence of at least a pair of 1-player and 2-player.

In particular, let us list the quantities in Eq. (S190) and clarify the calculation. Referring to Eq. (S16), we have

$$\langle\pi_{1|1}^{\mathbf{k}_{+1,+2}}\rangle=\sum_{k_1=0}^{k-2}\frac{(k-2)!}{k_1!(k-k_1-2)!}q_{1|1}{}^{k_1}q_{2|1}{}^{k-k_1-2}\pi_{1|1}^{\mathbf{k}_{+1,+2}}, \tag{S191a}$$

$$\langle\pi_{2|1}^{\mathbf{k}_{+1,+2}}\rangle=\sum_{k_1=0}^{k-2}\frac{(k-2)!}{k_1!(k-k_1-2)!}q_{1|1}{}^{k_1}q_{2|1}{}^{k-k_1-2}\pi_{2|1}^{\mathbf{k}_{+1,+2}}, \tag{S191b}$$

$$\langle\pi_{1|2}^{\mathbf{k}_{+1,+2}}\rangle=\sum_{k_1=0}^{k-2}\frac{(k-2)!}{k_1!(k-k_1-2)!}q_{1|2}{}^{k_1}q_{2|2}{}^{k-k_1-2}\pi_{1|2}^{\mathbf{k}_{+1,+2}}, \tag{S191c}$$

$$\langle\pi_{2|2}^{\mathbf{k}_{+1,+2}}\rangle=\sum_{k_1=0}^{k-2}\frac{(k-2)!}{k_1!(k-k_1-2)!}q_{1|2}{}^{k_1}q_{2|2}{}^{k-k_1-2}\pi_{2|2}^{\mathbf{k}_{+1,+2}}, \tag{S191d}$$

where, according to Eq. (S5), we have

$$\begin{aligned}\pi_{1|1}^{\mathbf{k}_{+1,+2}}=&\,a_{1|\mathbf{k}_{+1,+2}}+\sum_{k_1'=0}^{k-1}\frac{(k-1)!}{k_1'!(k-k_1'-1)!}q_{1|1}{}^{k_1'}q_{2|1}{}^{k-k_1'-1}\Bigg(a_{1|\mathbf{k}'_{+1}}+k_1'\sum_{k_1''=0}^{k-1}\frac{(k-1)!}{k_1''!(k-k_1''-1)!}q_{1|1}{}^{k_1''}q_{2|1}{}^{k-k_1''-1}a_{1|\mathbf{k}''_{+1}}\\&+(k-k_1'-1)\sum_{k_1''=0}^{k-1}\frac{(k-1)!}{k_1''!(k-k_1''-1)!}q_{1|2}{}^{k_1''}q_{2|2}{}^{k-k_1''-1}a_{1|\mathbf{k}''_{+2}}\Bigg),\end{aligned} \tag{S192a}$$

$$\begin{aligned}\pi_{2|1}^{\mathbf{k}_{+1,+2}}=&\,a_{2|\mathbf{k}_{+1,+1}}+\sum_{k_1'=0}^{k-1}\frac{(k-1)!}{k_1'!(k-k_1'-1)!}q_{1|2}{}^{k_1'}q_{2|2}{}^{k-k_1'-1}\Bigg(a_{2|\mathbf{k}'_{+1}}+k_1'\sum_{k_1''=0}^{k-1}\frac{(k-1)!}{k_1''!(k-k_1''-1)!}q_{1|1}{}^{k_1''}q_{2|1}{}^{k-k_1''-1}a_{2|\mathbf{k}''_{+1}}\\&+(k-k_1'-1)\sum_{k_1''=0}^{k-1}\frac{(k-1)!}{k_1''!(k-k_1''-1)!}q_{1|2}{}^{k_1''}q_{2|2}{}^{k-k_1''-1}a_{2|\mathbf{k}''_{+2}}\Bigg),\end{aligned} \tag{S192b}$$

$$\pi_{1|2}^{\mathbf{k}_{+1,+2}}=a_{1|\mathbf{k}_{+2,+2}}+\sum_{k_1'=0}^{k-1}\frac{(k-1)!}{k_1'!(k-k_1'-1)!}q_{1|1}{}^{k_1'}q_{2|1}{}^{k-k_1'-1}\Bigg(a_{1|\mathbf{k}'_{+2}}+k_1'\sum_{k_1''=0}^{k-1}\frac{(k-1)!}{k_1''!(k-k_1''-1)!}q_{1|1}{}^{k_1''}q_{2|1}{}^{k-k_1''-1}a_{1|\mathbf{k}''_{+1}}$$

$$+(k-k_1'-1)\sum_{k_1''=0}^{k-1}\frac{(k-1)!}{k_1''!(k-k_1''-1)!}q_{1|2}{}^{k_1''}q_{2|2}{}^{k-k_1''-1}a_{1|\mathbf{k}''_{+2}}\Bigg), \tag{S192c}$$

$$\pi_{2|2}^{\mathbf{k}_{+1,+2}}=a_{2|\mathbf{k}_{+1,+2}}+\sum_{k_1'=0}^{k-1}\frac{(k-1)!}{k_1'!(k-k_1'-1)!}q_{1|2}{}^{k_1'}q_{2|2}{}^{k-k_1'-1}\Bigg(a_{2|\mathbf{k}'_{+2}}+k_1'\sum_{k_1''=0}^{k-1}\frac{(k-1)!}{k_1''!(k-k_1''-1)!}q_{1|1}{}^{k_1''}q_{2|1}{}^{k-k_1''-1}a_{2|\mathbf{k}''_{+1}}$$

$$+(k-k_1'-1)\sum_{k_1''=0}^{k-1}\frac{(k-1)!}{k_1''!(k-k_1''-1)!}q_{1|2}{}^{k_1''}q_{2|2}{}^{k-k_1''-1}a_{2|\mathbf{k}''_{+2}}\Bigg). \tag{S192d}$$

In this way, the calculation of general 2-strategy multiplayer games given by Eq. (S190) is completed.

Similarly, after presenting the 2-strategy case by expected accumulated payoffs, we can further compute the results, which are divided into expected single-game payoffs. Under death-birth, however, the results would seem unruly and not easier than Eq. (S190). The 2-strategy case keeping expected single-game payoffs can be obtained by applying $n=2$ to Eq. (S184), which leads to an over-complicated result. In conclusion, we always need to treat the payoff structure of different models case by case, and using Eq. (S190) is an acceptable choice that we have.

In the case of special linear systems, however, we can refer to a well-organized general equation. Applying $n=2$ to Eq. (S187), we have

$$\dot{x}_1=\frac{\delta(k-2)}{k(k-1)^2}\Bigg\{\frac{(k^2-2)^2}{k}x_1(\bar{\pi}_1-\bar{\pi})+\frac{3k^2-4}{k}x_1(1-x_1)\left[k(b_{11}-b_{22})+c_1-c_2\right]$$

$$-(k^2+2k-4)x_1(1-x_1)\left[(b_{11}-b_{12})x_1+(b_{21}-b_{22})(1-x_1)\right]\Bigg\}, \tag{S193}$$

where b_{ij} and c_i are given by Eq. (S29) to express the linear payoff structure $a_{i|\mathbf{k}}=\sum_{l=1}^{n}b_{il}k_l+c_i$, and $x_1(\bar{\pi}_1-\bar{\pi})$ corresponds to the replicator dynamics in a well-mixed population, which can be obtained by existing knowledge of well-mixed populations.

Supplementary Note 5: Edge dynamics

As we know from Eq. (S1) in Supplementary Note 1.1, the system can be described by x_i and $q_{j|i}$. While we study the dynamics of x_i in previous sections, the dynamics of $q_{j|i}$ is further supplemented here. In particular, we show how to obtain the relation between x_i and $q_{j|i}$ as shown in Eqs. (S13) and (S178), for both pairwise comparison and death-birth. The same results have been declared in previous literature[2], but the details of the calculation process were unclear.

5.1 Pairwise comparison

First, we study edge dynamics under pairwise comparison to explain the origin of Eq. (S13) presented in Supplementary Note 2.3.

5.1.1 The increase of il-edges

The number of il-edges in the system increases by k_l when a j-player is replaced by an i-player ($j\neq i$)—the new i-player connects to the previous j-player's neighbors l, thus increasing the number of il-edges by k_l. Since the total number of edges in the system is $kN/2$, the frequency of il-edges, p_{il}, is increased by $k_l/(kN/2)=2k_l/(kN)$. For a possible neighbor configuration $\mathbf{k}$ of the j-player, we go through all $j\neq i$, obtaining the probability that the configuration $\mathbf{k}$ is found around the j-player and thus the frequency of il-edges increases by $2k_l/(kN)$:

$$\mathscr{P}\left(\Delta p_{il}=\frac{2k_l}{kN}\right)=\sum_{j=1,j\neq i}^{n}x_j\frac{k!}{\prod_{i'=1}^{n}k_{i'}!}\left(\prod_{i'=1}^{n}q_{i'|j}{}^{k_{i'}}\right)\mathscr{P}(j\leftarrow i). \tag{S194}$$

Then, we go through all possible configurations $\mathbf{k}$. We put this step late here because the change in the frequency of il-edges, $2k_l/(kN)$, varies in different $\mathbf{k}$. As we see, the expected change in the frequency of il-edges is calculated as:

$$\sum_{\sum_{i'=1}^{n}k_{i'}=k}\frac{2k_l}{kN}\mathscr{P}\left(\Delta p_{il}=\frac{2k_l}{kN}\right)=\sum_{\sum_{i'=1}^{n}k_{i'}=k}\frac{2k_l}{kN}\sum_{j=1,j\neq i}^{n}x_j\frac{k!}{\prod_{i'=1}^{n}k_{i'}!}\left(\prod_{i'=1}^{n}q_{i'|j}{}^{k_{i'}}\right)\mathscr{P}(j\leftarrow i)$$

$$=\sum_{\sum_{i'=1}^{n}k_{i'}=k}\frac{2k_l}{kN}\sum_{j=1,j\neq i}^{n}x_j\frac{k!}{\prod_{i'=1}^{n}k_{i'}!}\left(\prod_{i'=1}^{n}q_{i'|j}{}^{k_{i'}}\right)\left(\frac{k_i}{2k}+\mathscr{O}(\delta)\right)$$

$$= \begin{cases} \sum_{j=1,j\neq i}^{n} x_j \frac{q_{i|j}[(k-1)q_{i|j}+1]}{kN} + \mathscr{O}(\delta), & i=l, \\ \sum_{j=1,j\neq i}^{n} x_j \frac{(k-1)q_{l|j}q_{i|j}}{kN} + \mathscr{O}(\delta), & i\neq l. \end{cases}$$

$$= \frac{x_i}{kN} \begin{cases} \sum_{j=1,j\neq i}^{n} q_{j|i}[(k-1)q_{i|j}+1] + \mathscr{O}(\delta), & i=l, \\ \sum_{j=1,j\neq i}^{n} (k-1)q_{l|j}q_{j|i} + \mathscr{O}(\delta), & i\neq l. \end{cases} \tag{S195}$$

In Eq. (S195), $\mathscr{P}(j \leftarrow i)$ refers to Eq. (S8). We only take the first-order Taylor expansion, because the first-order term would be non-zero as we will see in Eq. (S198), thus not necessary to study the second-order term.

5.1.2 The decrease of il-edges

The number of il-edges in the system decreases by k_l when an i-player is replaced by a j-player ($j \neq i$). The died i-player disconnects from the previous neighbors l, thus decreasing the number of il-edges by k_l. In other words, the frequency of il-edges p_{il} is decreased by $k_l/(kN/2) = 2k_l/(kN)$. For a possible neighbor configuration $\mathbf{k}$ of the i-player, we go through all possible $j \neq i$, obtaining the probability that the configuration $\mathbf{k}$ is found around the i-player and thus the frequency of il-edges decreases by $2k_l/(kN)$:

$$\mathscr{P}\left(\Delta p_{il} = -\frac{2k_l}{kN}\right) = x_i \sum_{j=1,j\neq i}^{n} \frac{k!}{\prod_{i'=1}^{n} k_{i'}!} \left(\prod_{i'=1}^{n} {q_{i'|i}}^{k_{i'}}\right) \mathscr{P}(i \leftarrow j). \tag{S196}$$

Then, we go through all possible configurations $\mathbf{k}$. The expected change in the frequency of il-edges is calculated as:

$$\begin{aligned} \sum_{\sum_{i'=1}^{n} k_{i'}=k} \left(-\frac{2k_l}{kN}\right) \mathscr{P}\left(\Delta p_{il} = -\frac{2k_l}{kN}\right) &= \sum_{\sum_{i'=1}^{n} k_{i'}=k} \left(-\frac{2k_l}{kN}\right) x_i \sum_{j=1,j\neq i}^{n} \frac{k!}{\prod_{i'=1}^{n} k_{i'}!} \left(\prod_{i'=1}^{n} {q_{i'|i}}^{k_{i'}}\right) \mathscr{P}(i \leftarrow j) \\ &= \sum_{\sum_{i'=1}^{n} k_{i'}=k} \left(-\frac{2k_l}{kN}\right) x_i \sum_{j=1,j\neq i}^{n} \frac{k!}{\prod_{i'=1}^{n} k_{i'}!} \left(\prod_{i'=1}^{n} {q_{i'|i}}^{k_{i'}}\right) \left(\frac{k_j}{2k} + \mathscr{O}(\delta)\right) \\ &= \sum_{\sum_{i'=1}^{n} k_{i'}=k} \left(-\frac{2k_l}{kN}\right) x_i \frac{k!}{\prod_{i'=1}^{n} k_{i'}!} \left(\prod_{i'=1}^{n} {q_{i'|i}}^{k_{i'}}\right) \left(\frac{k-k_i}{2k} + \mathscr{O}(\delta)\right) \\ &= \begin{cases} x_i \frac{q_{i|i}}{N} - x_i \frac{q_{i|i}[(k-1)q_{i|i}+1]}{kN} + \mathscr{O}(\delta), & i=l, \\ x_i \frac{q_{l|i}}{N} - x_i \frac{(k-1)q_{l|j}q_{i|j}}{kN} + \mathscr{O}(\delta), & i\neq l. \end{cases} \\ &= \frac{x_i}{kN} \begin{cases} kq_{i|i} - q_{i|i}[(k-1)q_{i|i}+1] + \mathscr{O}(\delta), & i=l, \\ kq_{l|i} - (k-1)q_{l|i}q_{i|i} + \mathscr{O}(\delta), & i\neq l. \end{cases} \end{aligned} \tag{S197}$$

In Eq. (S197), $\mathscr{P}(i \leftarrow j)$ refers to Eq. (S10). Again, we only take the first-order Taylor expansion, because the first-order term would be non-zero as we will see in Eq. (S198), thus not necessary to study the second-order term.

5.1.3 Separation of different time scales

We combine the increase and decrease of il-edges given by Eqs. (S195) and (S197), and consider that a full MC step contains N elementary steps. In this way, we have the master equation of the change in the il-edge frequency:

$$\begin{aligned} \dot{p}_{il} &= N \times \left(\sum_{\sum_{i'=1}^{n} k_{i'}=k} \frac{2k_l}{kN} \mathscr{P}\left(\Delta p_{il} = \frac{2k_l}{kN}\right) + \sum_{\sum_{i'=1}^{n} k_{i'}=k} \left(-\frac{2k_l}{kN}\right) \mathscr{P}\left(\Delta p_{il} = -\frac{2k_l}{kN}\right) \right) \\ &= \frac{x_i}{k} \begin{cases} \sum_{j=1,j\neq i}^{n} q_{j|i}[(k-1)q_{i|j}+1] - kq_{i|i} + q_{i|i}[(k-1)q_{i|i}+1] + \mathscr{O}(\delta), & i=l, \\ \sum_{j=1,j\neq i}^{n} (k-1)q_{l|j}q_{j|i} - kq_{l|i} + (k-1)q_{l|i}q_{i|i} + \mathscr{O}(\delta), & i\neq l. \end{cases} \end{aligned}$$

$$
= \frac{x_i}{k} \begin{cases} \sum_{j=1}^{n} q_{j|i} + (k-1) \sum_{j=1}^{n} q_{l|j} q_{j|i} - k q_{l|i} + \mathscr{O}(\delta), & i = l, \\ (k-1) \sum_{j=1}^{n} q_{l|j} q_{j|i} - k q_{l|i} + \mathscr{O}(\delta), & i \neq l. \end{cases}
$$

$$
= \frac{x_i}{k} \left(\theta_{il} + (k-1) \sum_{j=1}^{n} q_{l|j} q_{j|i} - k q_{l|i} \right) + \mathscr{O}(\delta), \tag{S198}
$$

where

$$
\theta_{il} = \begin{cases} 1, & i = l, \\ 0, & i \neq l. \end{cases} \tag{S199}
$$

Previously, we conclude from Eq. (S1) in Supplementary Note 1.1 that the system can be expressed by x_i and $q_{j|i}$, eliminating the need to use p_{ij}. Therefore, we consider $q_{l|i} = p_{il}/x_i$ and write $\dot{q}_{l|i}$ by $\dot{p}_{il}$ as follows.

$$
\dot{q}_{l|i} = \frac{\mathrm{d}}{\mathrm{d}t} \left(\frac{p_{il}}{x_i} \right) = \frac{\dot{p}_{il} x_i - \dot{x}_i p_{il}}{x_i{}^2} = \frac{\dot{p}_{il}}{x_i} = \frac{1}{k} \left(\theta_{il} + (k-1) \sum_{j=1}^{n} q_{l|j} q_{j|i} - k q_{l|i} \right) + \mathscr{O}(\delta). \tag{S200}
$$

In Eq. (S200), we only take the first-order Taylor expansion, as the first-order term is non-zero and one can ignore the remaining higher order infinitesimals. It is noted that we have taken $\dot{x}_i = 0$ in the calculation, because as seen in Eq. (S12), $\dot{x}_i$ is zero in its δ^0 term and only becomes non-zero since the δ^1 term, which is the ignored higher order infinitesimals when calculating $\dot{q}_{l|i}$.

Here, an important characteristic arises: the time scales of the changes in $\dot{x}_i$ and $\dot{q}_{l|i}$ are different. Comparing Eqs. (S12) and (S200), we observe that the change in x_i happens at the δ^1 scale, while the change in $q_{l|i}$ happens at the δ^0 scale. In other words, the change of $q_{l|i}$ is much faster than in x_i.

Therefore, we can study the dynamics of x_i on the basis of $q_{l|i}$ achieving equilibrium (rapidly). That is, we can solve $\dot{q}_{l|i} = 0$, which is

$$
\theta_{il} + (k-1) \sum_{j=1}^{n} q_{l|j} q_{j|i} - k q_{l|i} = 0, \tag{S201}
$$

and represent all $q_{l|i}$ quantities by x_i quantities. Considering $x_i q_{l|i} = x_l q_{i|l}$, the solution of Eq. (S201) is

$$
q_{l|i} = \frac{(k-2)x_l + \theta_{il}}{k-1}, \tag{S202}
$$

which is Eq. (S13) presented in Supplementary Note 2.3, with the consideration of $\theta_{il} = 1$ if $i = l$ and $\theta_{il} = 0$ otherwise.

5.2 Death-birth

Similarly, we supplement edge dynamics under death-birth to explain the origin of Eq. (S178) presented in Supplementary Note 4.3.

5.2.1 The increase of il-edges

The number of il-edges in the system increases by k_l when a j-player is replaced by an i-player ($j \neq i$). The frequency of il-edges, p_{il}, is increased by $2k_l/(kN)$. For a possible neighbor configuration $\mathbf{k}$ of the j-player, we go through all $j \neq i$, obtaining the probability that the configuration $\mathbf{k}$ is found around the j-player and thus the frequency of il-edges increases by $2k_l/(kN)$:

$$
\mathscr{P}\left(\Delta p_{il} = \frac{2k_l}{kN}\right) = \sum_{j=1, j\neq i}^{n} x_j \frac{k!}{\prod_{i'=1}^{n} k_{i'}!} \left(\prod_{i'=1}^{n} q_{i'|j}{}^{k_{i'}} \right) \mathscr{P}(j \leftarrow i). \tag{S203}
$$

Then, we go through all possible configurations $\mathbf{k}$. The expected change in the frequency of il-edges is calculated as:

$$
\sum_{\sum_{i'=1}^{n} k_{i'} = k} \frac{2k_l}{kN} \mathscr{P}\left(\Delta p_{il} = \frac{2k_l}{kN}\right) = \sum_{\sum_{i'=1}^{n} k_{i'} = k} \frac{2k_l}{kN} \sum_{j=1, j\neq i}^{n} x_j \frac{k!}{\prod_{i'=1}^{n} k_{i'}!} \left(\prod_{i'=1}^{n} q_{i'|j}{}^{k_{i'}} \right) \mathscr{P}(j \leftarrow i)
$$

$$= \sum_{\sum_{i'=1}^{n} k_{i'}=k} \frac{2k_l}{kN} \sum_{j=1, j\neq i}^{n} x_j \frac{k!}{\prod_{i'=1}^{n} k_{i'}!} \left(\prod_{i'=1}^{n} q_{i'|j}{}^{k_{i'}} \right) \left(\frac{k_i}{k} + \mathscr{O}(\delta) \right)$$

$$= \frac{2x_i}{kN} \begin{cases} \sum_{j=1, j\neq i}^{n} q_{j|i}[(k-1)q_{i|j}+1] + \mathscr{O}(\delta), & i = l, \\ \sum_{j=1, j\neq i}^{n} (k-1)q_{l|j}q_{j|i} + \mathscr{O}(\delta), & i \neq l. \end{cases} \tag{S204}$$

In Eq. (S204), $\mathscr{P}(j \leftarrow i)$ refers to Eq. (S173). We only take the first-order Taylor expansion, because the first-order term would be non-zero as we will see in Eq. (S207).

5.2.2 The decrease of il-edges

The number of il-edges in the system decreases by k_l when an i-player is replaced by a j-player ($j \neq i$). The frequency of il-edges p_{il} is decreased by $2k_l/(kN)$. For a possible neighbor configuration $\mathbf{k}$ of the i-player, we go through all possible $j \neq i$, obtaining the probability that the configuration $\mathbf{k}$ is found around the i-player and thus the frequency of il-edges decreases by $2k_l/(kN)$:

$$\mathscr{P}\left(\Delta p_{il} = -\frac{2k_l}{kN}\right) = x_i \frac{k!}{\prod_{i'=1}^{n} k_{i'}!} \left(\prod_{i'=1}^{n} q_{i'|i}{}^{k_{i'}} \right) [1 - \mathscr{P}(i \leftarrow i)]. \tag{S205}$$

Then, we go through all possible configurations $\mathbf{k}$. The expected change in the frequency of il-edges is calculated as:

$$\sum_{\sum_{i'=1}^{n} k_{i'}=k} \left(-\frac{2k_l}{kN}\right) \mathscr{P}\left(\Delta p_{il} = -\frac{2k_l}{kN}\right) = \sum_{\sum_{i'=1}^{n} k_{i'}=k} \left(-\frac{2k_l}{kN}\right) x_i \frac{k!}{\prod_{i'=1}^{n} k_{i'}!} \left(\prod_{i'=1}^{n} q_{i'|i}{}^{k_{i'}} \right) [1 - \mathscr{P}(i \leftarrow i)]$$

$$= \sum_{\sum_{i'=1}^{n} k_{i'}=k} \left(-\frac{2k_l}{kN}\right) x_i \frac{k!}{\prod_{i'=1}^{n} k_{i'}!} \left(\prod_{i'=1}^{n} q_{i'|i}{}^{k_{i'}} \right) \left(1 - \frac{k_i}{k} + \mathscr{O}(\delta)\right)$$

$$= \frac{2x_i}{kN} \begin{cases} kq_{i|i} - q_{i|i}[(k-1)q_{i|i}+1] + \mathscr{O}(\delta), & i = l, \\ kq_{l|i} - (k-1)q_{l|i}q_{i|i} + \mathscr{O}(\delta), & i \neq l. \end{cases} \tag{S206}$$

In Eq. (S206), $[1 - \mathscr{P}(i \leftarrow i)]$ refers to Eq. (S175). Again, we only take the first-order Taylor expansion, because the first-order term would be non-zero as we will see in Eq. (S207).

5.2.3 Separation of different time scales

We combine the increase and decrease of il-edges given by Eqs. (S204) and (S206), and consider that a full MC step contains N elementary steps. In this way, we have the master equation of the change in the il-edge frequency under death-birth:

$$\dot{p}_{il} = N \times \left(\sum_{\sum_{i'=1}^{n} k_{i'}=k} \frac{2k_l}{kN} \mathscr{P}\left(\Delta p_{il} = \frac{2k_l}{kN}\right) + \sum_{\sum_{i'=1}^{n} k_{i'}=k} \left(-\frac{2k_l}{kN}\right) \mathscr{P}\left(\Delta p_{il} = -\frac{2k_l}{kN}\right) \right)$$

$$= \frac{2x_i}{k} \left(\theta_{il} + (k-1) \sum_{j=1}^{n} q_{l|j}q_{j|i} - kq_{l|i} \right) + \mathscr{O}(\delta), \tag{S207}$$

where $\theta_{il} = 1$ if $i = l$ and $\theta_{il} = 0$ otherwise.

According to Eq. (S1), we consider $q_{l|i} = p_{il}/x_i$ and write $\dot{q}_{l|i}$ by $\dot{p}_{il}$ as follows.

$$\dot{q}_{l|i} = \frac{\mathrm{d}}{\mathrm{d}t}\left(\frac{p_{il}}{x_i}\right) = \frac{2}{k}\left(\theta_{il} + (k-1) \sum_{j=1}^{n} q_{l|j}q_{j|i} - kq_{l|i} \right) + \mathscr{O}(\delta). \tag{S208}$$

In Eq. (S208), we only take the first-order Taylor expansion, as the first-order term is non-zero and one can ignore the remaining higher order infinitesimals. Comparing Eqs. (S177) and (S208), we observe that the change in x_i happens at the δ^1 scale, while the change in $q_{l|i}$ happens at the δ^0 scale. The change of $q_{l|i}$ are much faster than x_i.

Therefore, we can study the dynamics of x_i on the basis of $q_{l|i}$ achieving equilibrium (rapidly). That is, we can solve $\dot{q}_{l|i} = 0$, which is $\theta_{il} + (k-1)\sum_{j=1}^{n} q_{l|j}q_{j|i} - kq_{l|i} = 0$, and represent all $q_{l|i}$ quantities by x_i quantities. Considering $x_i q_{l|i} = x_l q_{i|l}$, the solution is

$$q_{l|i} = \frac{(k-2)x_l + \theta_{il}}{k-1}, \tag{S209}$$

which is Eq. (S178) presented in Supplementary Note 4.3, with the consideration of $\theta_{il} = 1$ if $i = l$ and $\theta_{il} = 0$ otherwise.

Supplementary Note 6: Theorem on computation and operators

A summation relation central to the theoretical derivation in this work is given by Theorem 1.

Theorem 1. *For any real number z_j, where $j = 1, 2, \ldots, n$, satisfying $0 \le z_j \le 1$ and $\sum_{j=1}^n z_j = 1$, and any scalar multivariate function $g(\mathbf{k})$ of $\mathbf{k}$, we have the following relation:*

$$\sum_{\sum_{j=1}^n k_j = k} \frac{k!}{\prod_{j=1}^n k_j!} \left(\prod_{j=1}^n z_j{}^{k_j} \right) k_i g(\mathbf{k}) = k z_i \sum_{\sum_{j=1}^n k_j = k-1} \frac{(k-1)!}{\prod_{j=1}^n k_j!} \left(\prod_{j=1}^n z_j{}^{k_j} \right) g(\mathbf{k}_{+i}). \tag{S210}$$

Proof. Recall the definitions given in the main text or Supplementary Note 1: $\mathbf{k} = (k_1, k_2, \ldots, k_n)$, where $\sum_{j=1}^n k_j = k$; and $\mathbf{k}_{+i} = (k_1, k_2, \ldots, k_i + 1, \ldots, k_n)$, where $\sum_{j=1}^n k_j = k - 1$. In this way, we have

$$\begin{aligned} \sum_{\sum_{j=1}^n k_j = k} \frac{k!}{\prod_{j=1}^n k_j!} \left(\prod_{j=1}^n z_j{}^{k_j} \right) k_i g(\mathbf{k}) &= k z_i \sum_{\sum_{j=1}^n k_j = k, k_i \neq 0} \frac{(k-1)!}{(k_i - 1)! \prod_{j=1, j\neq i}^n k_j!} \left(z_i{}^{k_i - 1} \prod_{j=1, j\neq i}^n z_j{}^{k_j} \right) g(\mathbf{k}) \\ &= k z_i \sum_{\sum_{j=1}^n k_j = k-1} \frac{(k-1)!}{\prod_{j=1}^n k_j!} \left(\prod_{j=1}^n z_j{}^{k_j} \right) g(\mathbf{k}_{+i}). \end{aligned} \tag{S211}$$

The first step of Eq. (S211) uses $k! = k(k-1)!$, $z_i{}^{k_i} = z_i z_i{}^{k_i - 1}$, $k_i/(k_i!) = 1/[(k_i - 1)!]$ and considers that the entire formula equals to zero when $k_i = 0$. The second step of Eq. (S211) is a straightforward application of the $\mathbf{k}_{+i}$ concept. □

Theorem 1 gives a general relation which helps us eliminate k_i inside summations at the cost of transforming $g(\mathbf{k})$ into $g(\mathbf{k}_{+i})$. For convenience of our readers, there are several useful applications of Theorem 1.

For example, taking $g(\mathbf{k}) = 1$, we have

$$\sum_{\sum_{j=1}^n k_j = k} \frac{k!}{\prod_{j=1}^n k_j!} \left(\prod_{j=1}^n z_j{}^{k_j} \right) k_i = k z_i. \tag{S212}$$

Another example is $g(\mathbf{k}) = k_l$. If $l = i$, we have

$$\sum_{\sum_{j=1}^n k_j = k} \frac{k!}{\prod_{j=1}^n k_j!} \left(\prod_{j=1}^n z_j{}^{k_j} \right) k_i k_l = k z_i \sum_{\sum_{j=1}^n k_j = k-1} \frac{(k-1)!}{\prod_{j=1}^n k_j!} \left(\prod_{j=1}^n z_j{}^{k_j} \right) (k_l + 1) = k z_i [(k-1) z_l + 1], \tag{S213}$$

and if $l \neq i$, it becomes

$$\sum_{\sum_{j=1}^n k_j = k} \frac{k!}{\prod_{j=1}^n k_j!} \left(\prod_{j=1}^n z_j{}^{k_j} \right) k_i k_l = k z_i \sum_{\sum_{j=1}^n k_j = k-1} \frac{(k-1)!}{\prod_{j=1}^n k_j!} \left(\prod_{j=1}^n z_j{}^{k_j} \right) k_l = k(k-1) z_i z_l. \tag{S214}$$

Finally, a potentially misleading example is $g(\mathbf{k}) = k'_l$, where $k'_l \in \mathbf{k}' \neq \mathbf{k}$ (recall that $\mathbf{k}'$ is an independent variable of $\mathbf{k}$). In this way, elements in $\mathbf{k}'$ are essentially constants when studying $g(\mathbf{k})$,

$$\sum_{\sum_{j=1}^n k_j = k} \frac{k!}{\prod_{j=1}^n k_j!} \left(\prod_{j=1}^n z_j{}^{k_j} \right) k_i k'_l = k z_i k'_l. \tag{S215}$$

In this work, we have created many operators to convey various concepts. The main relations between these operators are summarized in Theorems 2–5.

Theorem 2. *The following equation holds:*

$$\langle \pi_{i|j}^{\mathbf{k}_{+i}} \rangle = \langle \pi_i^{\mathbf{k}_{+j}} \rangle. \tag{S216}$$

Intuitively speaking, these two concepts are equivalent: (1) The expected accumulated payoff of an i-player neighboring a j-player, where the j-player has at least one i-player neighbor. (2) The expected accumulated payoff of an i-player, where the i-player has at least one j-player neighbor.

Proof. Let us expand $\pi_{i|j}^{\mathbf{k}_{+i}}$ according to Eq. (S5),

$$\pi_{i|j}^{\mathbf{k}_{+i}} = a_{i|\mathbf{k}_{+j}} + \sum_{\sum_{l=1}^{n} k'_l = k-1} \frac{(k-1)!}{\prod_{l=1}^{n} k'_l!} \left(\prod_{l=1}^{n} q_{l|i}{}^{k'_l} \right) \left(a_{i|\mathbf{k}'_{+j}} + \sum_{l=1}^{n} k'_l \sum_{\sum_{\ell=1}^{n} k''_\ell = k-1} \frac{(k-1)!}{\prod_{\ell=1}^{n} k''_\ell!} \left(\prod_{\ell=1}^{n} q_{\ell|l}{}^{k''_\ell} \right) a_{i|\mathbf{k}''_{+l}} \right). \tag{S217}$$

By substituting Eq. (S217) into Eq. (S16), we calculate

$$\begin{aligned}
\langle \pi_{i|j}^{\mathbf{k}_{+i}} \rangle = & \sum_{\sum_{i'=1}^{n} k_{i'} = k-1} \frac{(k-1)!}{\prod_{i'=1}^{n} k_{i'}!} \left(\prod_{i'=1}^{n} q_{i'|j}{}^{k_{i'}} \right) a_{i|\mathbf{k}_{+j}} \\
& + \sum_{\sum_{i'=1}^{n} k_{i'} = k-1} \frac{(k-1)!}{\prod_{i'=1}^{n} k_{i'}!} \left(\prod_{i'=1}^{n} q_{i'|j}{}^{k_{i'}} \right) \sum_{\sum_{l=1}^{n} k'_l = k-1} \frac{(k-1)!}{\prod_{l=1}^{n} k'_l!} \left(\prod_{l=1}^{n} q_{l|i}{}^{k'_l} \right) \Big[a_{i|\mathbf{k}'_{+j}} \\
& + \sum_{l=1}^{n} k'_l \sum_{\sum_{\ell=1}^{n} k''_\ell = k-1} \frac{(k-1)!}{\prod_{\ell=1}^{n} k''_\ell!} \left(\prod_{\ell=1}^{n} q_{\ell|l}{}^{k''_\ell} \right) a_{i|\mathbf{k}''_{+l}} \Big] \\
= & \sum_{\sum_{i'=1}^{n} k_{i'} = k-1} \frac{(k-1)!}{\prod_{i'=1}^{n} k_{i'}!} \left(\prod_{i'=1}^{n} q_{i'|j}{}^{k_{i'}} \right) a_{i|\mathbf{k}_{+j}} + \sum_{\sum_{l=1}^{n} k'_l = k-1} \frac{(k-1)!}{\prod_{l=1}^{n} k'_l!} \left(\prod_{l=1}^{n} q_{l|i}{}^{k'_l} \right) a_{i|\mathbf{k}'_{+j}} \\
& + \sum_{\sum_{l=1}^{n} k'_l = k-1} \frac{(k-1)!}{\prod_{l=1}^{n} k'_l!} \left(\prod_{l=1}^{n} q_{l|i}{}^{k'_l} \right) \sum_{l=1}^{n} k'_l \sum_{\sum_{\ell=1}^{n} k''_\ell = k-1} \frac{(k-1)!}{\prod_{\ell=1}^{n} k''_\ell!} \left(\prod_{\ell=1}^{n} q_{\ell|l}{}^{k''_\ell} \right) a_{i|\mathbf{k}''_{+l}}.
\end{aligned} \tag{S218}$$

Similarly, we expand $\pi_i^{\mathbf{k}_{+j}}$ according to Eq. (S3),

$$\pi_i^{\mathbf{k}_{+j}} = a_{i|\mathbf{k}_{+j}} + \sum_{l=1}^{n} k_l \sum_{\sum_{\ell=1}^{n} k'_\ell = k-1} \frac{(k-1)!}{\prod_{\ell=1}^{n} k'_\ell!} \left(\prod_{\ell=1}^{n} q_{\ell|l}{}^{k'_\ell} \right) a_{i|\mathbf{k}'_{+l}}. \tag{S219}$$

By substituting Eq. (S219) into Eq. (S17), we calculate

$$\begin{aligned}
\langle \pi_i^{\mathbf{k}_{+j}} \rangle = & \sum_{\sum_{i'=1}^{n} k_{i'} = k-1} \frac{(k-1)!}{\prod_{i'=1}^{n} k_{i'}!} \left(\prod_{i'=1}^{n} q_{i'|i}{}^{k_{i'}} \right) \pi_i^{\mathbf{k}_{+j}} \\
= & \sum_{\sum_{i'=1}^{n} k_{i'} = k-1} \frac{(k-1)!}{\prod_{i'=1}^{n} k_{i'}!} \left(\prod_{i'=1}^{n} q_{i'|i}{}^{k_{i'}} \right) a_{i|\mathbf{k}_{+j}} + \sum_{\sum_{\ell=1}^{n} k'_\ell = k-1} \frac{(k-1)!}{\prod_{\ell=1}^{n} k'_\ell!} \left(\prod_{\ell=1}^{n} q_{\ell|j}{}^{k'_\ell} \right) a_{i|\mathbf{k}'_{+j}} \\
& + \sum_{\sum_{i'=1}^{n} k_{i'} = k-1} \frac{(k-1)!}{\prod_{i'=1}^{n} k_{i'}!} \left(\prod_{i'=1}^{n} q_{i'|i}{}^{k_{i'}} \right) \sum_{l=1}^{n} k_l \sum_{\sum_{\ell=1}^{n} k'_\ell = k-1} \frac{(k-1)!}{\prod_{\ell=1}^{n} k'_\ell!} \left(\prod_{\ell=1}^{n} q_{\ell|l}{}^{k'_\ell} \right) a_{i|\mathbf{k}'_{+l}}.
\end{aligned} \tag{S220}$$

Comparing Eqs. (S218) and (S220) and realizing the equivalence of auxiliary variables, we have $\langle \pi_{i|j}^{\mathbf{k}_{+i}} \rangle = \langle \pi_i^{\mathbf{k}_{+j}} \rangle$. □

Theorem 3. *The following equation holds:*

$$\sum_{j=1}^{n} q_{j|i} \langle \pi_{i|j}^{\mathbf{k}_{+i}} \rangle = \langle \pi_i^{\mathbf{k}} \rangle. \tag{S221}$$

Intuitive interpretation: (1) the expected accumulated payoff of an i-player over all possible neighboring j-players found near an i-player, where the j-player has at least one i-player neighbor, is equivalent to (2) the expected accumulated payoff of an i-player.

Proof. Further developing Eq. (S218) results in

$$\sum_{j=1}^{n} q_{j|i} \langle \pi_{i|j}^{\mathbf{k}_{+i}} \rangle = \sum_{j=1}^{n} q_{j|i} \sum_{\sum_{i'=1}^{n} k_{i'} = k-1} \frac{(k-1)!}{\prod_{i'=1}^{n} k_{i'}!} \left(\prod_{i'=1}^{n} q_{i'|j}{}^{k_{i'}} \right) a_{i|\mathbf{k}_{+j}} + \sum_{j=1}^{n} q_{j|i} \sum_{\sum_{l=1}^{n} k'_l = k-1} \frac{(k-1)!}{\prod_{l=1}^{n} k'_l!} \left(\prod_{l=1}^{n} q_{l|i}{}^{k'_l} \right) a_{i|\mathbf{k}'_{+j}}$$

$$+\sum_{j=1}^{n} q_{j|i} \sum_{\sum_{l=1}^{n} k_l'=k-1} \frac{(k-1)!}{\prod_{l=1}^{n} k_l'!} \left(\prod_{l=1}^{n} q_{l|i}{}^{k_l'}\right) \sum_{l=1}^{n} k_l' \sum_{\sum_{\ell=1}^{n} k_\ell''=k-1} \frac{(k-1)!}{\prod_{\ell=1}^{n} k_\ell''!} \left(\prod_{\ell=1}^{n} q_{\ell|l}{}^{k_\ell''}\right) a_{i|\mathbf{k}''_{+l}}$$

$$=\sum_{j=1}^{n} q_{j|i} \sum_{\sum_{i'=1}^{n} k_{i'}=k-1} \frac{(k-1)!}{\prod_{i'=1}^{n} k_{i'}!} \left(\prod_{i'=1}^{n} q_{i'|j}{}^{k_{i'}}\right) a_{i|\mathbf{k}_{+j}} + \sum_{j=1}^{n} \frac{k_j}{k} \sum_{\sum_{l=1}^{n} k_l'=k} \frac{k!}{\prod_{l=1}^{n} k_l'!} \left(\prod_{l=1}^{n} q_{l|i}{}^{k_l'}\right) a_{i|\mathbf{k}'}$$

$$+(k-1) \sum_{\sum_{l=1}^{n} k_l'=k-2} \frac{(k-2)!}{\prod_{l=1}^{n} k_l'!} \left(\prod_{l=1}^{n} q_{l|i}{}^{k_l'}\right) \sum_{l=1}^{n} q_{l|i} \sum_{\sum_{\ell=1}^{n} k_\ell''=k-1} \frac{(k-1)!}{\prod_{\ell=1}^{n} k_\ell''!} \left(\prod_{\ell=1}^{n} q_{\ell|l}{}^{k_\ell''}\right) a_{i|\mathbf{k}''_{+l}}$$

$$=\sum_{j=1}^{n} q_{j|i} \sum_{\sum_{i'=1}^{n} k_{i'}=k-1} \frac{(k-1)!}{\prod_{i'=1}^{n} k_{i'}!} \left(\prod_{i'=1}^{n} q_{i'|j}{}^{k_{i'}}\right) a_{i|\mathbf{k}_{+j}} + \sum_{\sum_{l=1}^{n} k_l'=k} \frac{k!}{\prod_{l=1}^{n} k_l'!} \left(\prod_{l=1}^{n} q_{l|i}{}^{k_l'}\right) a_{i|\mathbf{k}'}$$

$$+(k-1) \sum_{l=1}^{n} q_{l|i} \sum_{\sum_{\ell=1}^{n} k_\ell''=k-1} \frac{(k-1)!}{\prod_{\ell=1}^{n} k_\ell''!} \left(\prod_{\ell=1}^{n} q_{\ell|l}{}^{k_\ell''}\right) a_{i|\mathbf{k}''_{+l}}$$

$$=\sum_{\sum_{l=1}^{n} k_l'=k} \frac{k!}{\prod_{l=1}^{n} k_l'!} \left(\prod_{l=1}^{n} q_{l|i}{}^{k_l'}\right) a_{i|\mathbf{k}'} + k \sum_{l=1}^{n} q_{l|i} \sum_{\sum_{\ell=1}^{n} k_\ell''=k-1} \frac{(k-1)!}{\prod_{\ell=1}^{n} k_\ell''!} \left(\prod_{\ell=1}^{n} q_{\ell|l}{}^{k_\ell''}\right) a_{i|\mathbf{k}''_{+l}}. \tag{S222}$$

On the other hand, we apply the expression of $\pi_i^{\mathbf{k}}$ in Eq. (S3) to Eq. (S17) and calculate

$$\langle \pi_i^{\mathbf{k}} \rangle = \sum_{\sum_{i'=1}^{n} k_{i'}=k} \frac{k!}{\prod_{i'=1}^{n} k_{i'}!} \left(\prod_{i'=1}^{n} q_{i'|i}{}^{k_{i'}}\right) \pi_i^{\mathbf{k}}$$

$$= \sum_{\sum_{i'=1}^{n} k_{i'}=k} \frac{k!}{\prod_{i'=1}^{n} k_{i'}!} \left(\prod_{i'=1}^{n} q_{i'|i}{}^{k_{i'}}\right) a_{i|\mathbf{k}}$$

$$+ \sum_{\sum_{i'=1}^{n} k_{i'}=k} \frac{k!}{\prod_{i'=1}^{n} k_{i'}!} \left(\prod_{i'=1}^{n} q_{i'|i}{}^{k_{i'}}\right) \sum_{l=1}^{n} k_l \sum_{\sum_{\ell=1}^{n} k_\ell'=k-1} \frac{(k-1)!}{\prod_{\ell=1}^{n} k_\ell'!} \left(\prod_{\ell=1}^{n} q_{\ell|l}{}^{k_\ell'}\right) a_{i|\mathbf{k}'_{+l}}$$

$$= \sum_{\sum_{i'=1}^{n} k_{i'}=k} \frac{k!}{\prod_{i'=1}^{n} k_{i'}!} \left(\prod_{i'=1}^{n} q_{i'|i}{}^{k_{i'}}\right) a_{i|\mathbf{k}} + k \sum_{l=1}^{n} q_{l|i} \sum_{\sum_{\ell=1}^{n} k_\ell'=k-1} \frac{(k-1)!}{\prod_{\ell=1}^{n} k_\ell'!} \left(\prod_{\ell=1}^{n} q_{\ell|l}{}^{k_\ell'}\right) a_{i|\mathbf{k}'_{+l}}. \tag{S223}$$

Comparing Eqs. (S222) and (S223) and noting that $\mathbf{k}'$, $\mathbf{k}''_{+l}$, $\mathbf{k}$, and $\mathbf{k}'_{+l}$ are calculated separately here (i.e., we can treat $\mathbf{k}'$, $\mathbf{k}$ as $\mathbf{k}$ and treat $\mathbf{k}''_{+l}$, $\mathbf{k}'_{+l}$ as $\mathbf{k}_{+l}$), we have $\sum_{j=1}^{n} q_{j|i} \langle \pi_{i|j}^{\mathbf{k}_{+i}} \rangle = \langle \pi_i^{\mathbf{k}} \rangle$. □

Theorem 4. *The following equation holds:*

$$\langle a_{i|\mathbf{k}} \rangle_i = \sum_{j=1}^{n} q_{j|i} \langle a_{i|\mathbf{k}_{+j}} \rangle_i. \tag{S224}$$

Intuitive interpretation: (1) the expected single-game payoff of an i-player is equivalent to (2) the expected single-game payoff of an i-player conditioning on all possible neighboring j-players, where the i-player has at least one j-player neighbor.

Proof. According to the definition of $\langle a_{i|\mathbf{k}} \rangle_j$ in Eq. (S21), we have

$$\langle a_{i|\mathbf{k}} \rangle_i = \sum_{\sum_{i'=1}^{n} k_{i'}=k} \frac{k!}{\prod_{i'=1}^{n} k_{i'}!} \left(\prod_{i'=1}^{n} q_{i'|i}{}^{k_{i'}}\right) a_{i|\mathbf{k}}$$

$$= \sum_{j=1}^{n} \sum_{\sum_{i'=1}^{n} k_{i'}=k} \frac{k!}{\prod_{i'=1}^{n} k_{i'}!} \left(\prod_{i'=1}^{n} q_{i'|i}{}^{k_{i'}}\right) \frac{k_j}{k} a_{i|\mathbf{k}}$$

$$= \sum_{j=1}^{n} q_{j|i} \sum_{\sum_{i'=1}^{n} k_{i'}=k-1} \frac{(k-1)!}{\prod_{i'=1}^{n} k_{i'}!} \left(\prod_{i'=1}^{n} q_{i'|i}{}^{k_{i'}}\right) a_{i|\mathbf{k}_{+j}}$$

$$= \sum_{j=1}^{n} q_{j|i} \langle a_{i|\mathbf{k}_{+j}} \rangle_i, \tag{S225}$$

which completes the proof. □

Theorem 5. *The following equation holds:*

$$\sum_{j=1}^{n} \sum_{i'=1}^{n} q_{i'|j} q_{j|i} \langle \pi_{i|j}^{\mathbf{k}_{+i,+i'}} \rangle = \langle \pi_i^{\mathbf{k}} \rangle. \tag{S226}$$

Intuitive interpretation: (1) the expected accumulated payoff of an i-player over all possible neighboring j-players found near an i-player and all possible neighboring i′-players found near an j-player, where the j-player has at least one i-player neighbor and one i′-player neighbor, is equivalent to (2) the expected accumulated payoff of an i-player.

Proof. According to Theorem 3, we have $\langle \pi_i^{\mathbf{k}} \rangle = \sum_{j=1}^{n} q_{j|i} \langle \pi_{i|j}^{\mathbf{k}_{+i}} \rangle$. Furthermore, we calculate

$$\begin{aligned}
\langle \pi_{i|j}^{\mathbf{k}_{+i}} \rangle &= \sum_{\sum_{i'=1}^{n} k_{i'} = k-1} \frac{(k-1)!}{\prod_{i'=1}^{n} k_{i'}!} \left(\prod_{i'=1}^{n} {q_{i'|j}}^{k_{i'}} \right) \pi_{i|j}^{\mathbf{k}_{+i}} \\
&= \sum_{\sum_{i'=1}^{n} k_{i'} = k-1} \frac{(k-1)!}{\prod_{i'=1}^{n} k_{i'}!} \left(\prod_{i'=1}^{n} {q_{i'|j}}^{k_{i'}} \right) \sum_{i'=1}^{n} \frac{k_{i'}}{k-1} \pi_{i|j}^{\mathbf{k}_{+i}} \\
&= \sum_{\sum_{i'=1}^{n} k_{i'} = k-2} \frac{(k-2)!}{\prod_{i'=1}^{n} k_{i'}!} \left(\prod_{i'=1}^{n} {q_{i'|j}}^{k_{i'}} \right) \sum_{i'=1}^{n} q_{i'|j} \pi_{i|j}^{\mathbf{k}_{+i,+i'}} \\
&= \sum_{i'=1}^{n} q_{i'|j} \langle \pi_{i|j}^{\mathbf{k}_{+i,+i'}} \rangle.
\end{aligned} \tag{S227}$$

Applying Eq. (S227) to Theorem 3, we obtain $\langle \pi_i^{\mathbf{k}} \rangle = \sum_{j=1}^{n} \sum_{i'=1}^{n} q_{i'|j} q_{j|i} \langle \pi_{i|j}^{\mathbf{k}_{+i,+i'}} \rangle$, which completes the proof. □

References

1. Ohtsuki, H., Hauert, C., Lieberman, E. & Nowak, M. A. A simple rule for the evolution of cooperation on graphs and social networks. *Nature* **441**, 502–505, DOI:10.1038/nature04605 (2006).
2. Ohtsuki, H. & Nowak, M. A. The replicator equation on graphs. *J. Theor. Biol.* **243**, 86–97, DOI:10.1016/j.jtbi.2006.06.004 (2006).
3. Li, A., Broom, M., Du, J. & Wang, L. Evolutionary dynamics of general group interactions in structured populations. *Phys. Rev. E* **93**, 022407, DOI:10.1103/PhysRevE.93.022407 (2016).
4. Gutowitz, H. A., Victor, J. D. & Knight, B. W. Local structure theory for cellular automata. *Phys. D* **28**, 18–48, DOI:10.1016/0167-2789(87)90120-5 (1987).
5. Szabó, G., Szolnoki, A. & Bodócs, L. Correlations induced by transport in one-dimensional lattice gas. *Phys. Rev. A* **44**, 6375, DOI:10.1103/PhysRevA.44.6375 (1991).
6. McAvoy, A., Allen, B. & Nowak, M. A. Social goods dilemmas in heterogeneous societies. *Nat. Hum. Behav.* **4**, 819–831, DOI:10.1038/s41562-020-0881-2 (2020).
7. Wang, C. & Szolnoki, A. Inertia in spatial public goods games under weak selection. *Appl. Math. Comput.* **449**, 127941, DOI:10.1016/j.amc.2023.127941 (2023).
8. Szabó, G. & Tőke, C. Evolutionary prisoner's dilemma game on a square lattice. *Phys. Rev. E* **58**, 69, DOI:10.1103/PhysRevE.58.69 (1998).
9. Allen, B. & Nowak, M. A. Games on graphs. *EMS Surv. Math. Sci.* **1**, 113–151, DOI:10.4171/EMSS/3 (2014).
10. Allen, B. *et al.* Evolutionary dynamics on any population structure. *Nature* **544**, 227–230, DOI:10.1038/nature21723 (2017).
11. Zhang, W. & Brandes, U. Is cooperation sustained under increased mixing in evolutionary public goods games on networks? *Appl. Math. Comput.* **438**, 127604, DOI:10.1016/j.amc.2022.127604 (2023).
12. Li, A., Wu, B. & Wang, L. Cooperation with both synergistic and local interactions can be worse than each alone. *Sci. Reports* **4**, 5536, DOI:10.1038/srep05536 (2014).
13. Su, Q., Li, A., Wang, L. & Eugene Stanley, H. Spatial reciprocity in the evolution of cooperation. *Proc. Royal Soc. B* **286**, 20190041, DOI:10.1098/rspb.2019.0041 (2019).
14. Wang, C., Zhu, W. & Szolnoki, A. When greediness and self-confidence meet in a social dilemma. *Phys. A* **625**, 129033, DOI:10.1016/j.physa.2023.129033 (2023).
15. Szolnoki, A. & Chen, X. Tactical cooperation of defectors in a multi-stage public goods game. *Chaos, Solitons & Fractals* **155**, 111696, DOI:10.1016/j.chaos.2021.111696 (2022).
16. Helbing, D., Szolnoki, A., Perc, M. & Szabó, G. Evolutionary establishment of moral and double moral standards through spatial interactions. *PLoS Comput. Biol.* **6**, e1000758, DOI:10.1371/journal.pcbi.1000758 (2010).
17. Szolnoki, A., Szabó, G. & Perc, M. Phase diagrams for the spatial public goods game with pool punishment. *Phys. Rev. E* **83**, 036101, DOI:10.1103/PhysRevE.83.036101 (2011).
18. Szolnoki, A. & Perc, M. Reward and cooperation in the spatial public goods game. *Europhys. Lett.* **92**, 38003, DOI:10.1209/0295-5075/92/38003 (2010).
19. Perc, M., Gómez-Gardenes, J., Szolnoki, A., Floría, L. M. & Moreno, Y. Evolutionary dynamics of group interactions on structured populations: a review. *J. Royal Soc. Interface* **10**, 20120997, DOI:10.1098/rsif.2012.0997 (2013).
